# Activation of an endogenous retrovirus-associated long non-coding RNA in human adenocarcinoma

**DOI:** 10.1186/s13073-015-0142-6

**Published:** 2015-03-05

**Authors:** Ewan A Gibb, René L Warren, Gavin W Wilson, Scott D Brown, Gordon A Robertson, Gregg B Morin, Robert A Holt

**Affiliations:** Genome Sciences Centre, British Columbia Cancer Agency, 675 West 10th Ave, Vancouver, British Columbia V5Z 1L3 Canada; Department of Medical Genetics, University of British Columbia, Vancouver, British Columbia V6T 1Z4 Canada; Informatics and Biocomputing Platform, Ontario Institute for Cancer Research, Toronto, Ontario M5G 0A3 Canada; Department of Molecular Genetics, University of Toronto, Toronto, Ontario M5S 1A8 Canada; Genome Science and Technology Program, University of British Columbia, Vancouver, British Columbia V6T 1Z4 Canada; Department of Molecular Biology and Biochemistry, Simon Fraser University, Burnaby, British Columbia V5A 1S6 Canada

## Abstract

**Background:**

Long non-coding RNAs (lncRNAs) are emerging as molecules that significantly impact many cellular processes and have been associated with almost every human cancer. Compared to protein-coding genes, lncRNA genes are often associated with transposable elements, particularly with endogenous retroviral elements (ERVs). ERVs can have potentially deleterious effects on genome structure and function, so these elements are typically silenced in normal somatic tissues, albeit with varying efficiency. The aberrant regulation of ERVs associated with lncRNAs (ERV-lncRNAs), coupled with the diverse range of lncRNA functions, creates significant potential for ERV-lncRNAs to impact cancer biology.

**Methods:**

We used RNA-seq analysis to identify and profile the expression of a novel lncRNA in six large cohorts, including over 7,500 samples from The Cancer Genome Atlas (TCGA).

**Results:**

We identified the tumor-specific expression of a novel lncRNA that we have named Endogenous retroViral-associated ADenocarcinoma RNA or ‘*EVADR*’, by analyzing RNA-seq data derived from colorectal tumors and matched normal control tissues. Subsequent analysis of TCGA RNA-seq data revealed the striking association of *EVADR* with adenocarcinomas, which are tumors of glandular origin. Moderate to high levels of *EVADR* were detected in 25 to 53% of colon, rectal, lung, pancreas and stomach adenocarcinomas (mean = 30 to 144 FPKM), and *EVADR* expression correlated with decreased patient survival (Cox regression; hazard ratio = 1.47, 95% confidence interval = 1.06 to 2.04, *P* = 0.02). In tumor sites of non-glandular origin, *EVADR* expression was detectable at only very low levels and in less than 10% of patients. For *EVADR*, a MER48 ERV element provides an active promoter to drive its transcription. Genome-wide, MER48 insertions are associated with nine lncRNAs, but none of the MER48-associated lncRNAs other than *EVADR* were consistently expressed in adenocarcinomas, demonstrating the specific activation of *EVADR*. The sequence and structure of the *EVADR* locus is highly conserved among Old World monkeys and apes but not New World monkeys or prosimians, where the MER48 insertion is absent. Conservation of the *EVADR* locus suggests a functional role for this novel lncRNA in humans and our closest primate relatives.

**Conclusions:**

Our results describe the specific activation of a highly conserved ERV-lncRNA in numerous cancers of glandular origin, a finding with diagnostic, prognostic and therapeutic implications.

**Electronic supplementary material:**

The online version of this article (doi:10.1186/s13073-015-0142-6) contains supplementary material, which is available to authorized users.

## Background

The mammalian transcriptome is pervasively transcribed [1-4]. Comprehensive transcriptome sequencing surveys have revealed many classes of widely expressed non-coding RNAs, including long non-coding RNAs (lncRNAs) [5]. These novel genes encode mRNA-like transcripts that are by definition at least 200 nucleotides in length and have no apparent protein coding capacity, but are otherwise subject to normal mRNA processing, including 5′ capping, splicing and polyadenylation [6]. Many lncRNAs demonstrate exquisite cellular-, tissue- or developmental stage-specific expression patterns [7-9]. lncRNAs have a range of demonstrated functions, including chromatin remodeling [10], alternative splicing [11] and mRNA degradation [12], and their dysregulation has been linked to many disorders, including cancer [13-15].

Compared with protein-coding genes, lncRNA genes tend to associate with transposable elements, particularly with endogenous retroviruses (ERVs) [16-18]. The majority of ERVs are genomic relics of exogenous retrovirus insertions which, although typically degenerated, may retain active promoter and polyadenylation signals encoded within their flanking long terminal repeats (LTRs) [19,20]. Through these active regulatory elements, both ERVs and other retrotransposons contribute significantly to the regulation of gene expression [21,22]. However, unregulated ERV LTRs can promote aberrant transcription and are therefore typically silenced in adult tissues by epigenetic mechanisms including, but not limited to, DNA methylation [23,24]. In cancer, silenced ERVs may be released from normal cellular regulation, resulting in a general increase in ERV-mediated transcription [25-27]. Given the frequency and scattered genomic distribution of ERVs and the influence of their LTRs on the expression of neighboring genes, ERVs have high potential to promote oncogene expression or to alter host gene expression networks to favor tumor development [25]. Similarly, changes in physiological or cellular conditions that promote class-specific ERV LTR expression could promote the activation of lncRNAs associated with that class, as has been observed for HERV-H LTRs in embryonic stem cells [18,28]. Considering the range of biological functions described for lncRNAs and their role in gene regulation, ERV-mediated lncRNA activation has considerable potential to impact cellular biology.

To further characterize the relationship between aberrant lncRNA expression and cancer, we evaluated RNA-seq data from colorectal adenocarcinoma and matched normal control tissues from 65 subjects for lncRNA expression. We identified a 394-nucleotide MER48 LTR ERV-associated lncRNA ‘*EVADR*’ as being robustly expressed in tumor samples, with virtually no expression in corresponding normal tissues. We then profiled the *EVADR* lncRNA across 25 different cancer types, determined the promoter activity of the MER48 LTR *in vitro*, mapped the genome-wide MER48 LTR expression, and surveyed the conservation of the *EVADR* gene locus across 13 primates. Here we report the identification and initial characterization of *EVADR*.

## Methods

### Cancer, normal tissue and cell line transcriptome datasets

All sequence files were obtained with permission and stored in a secure file system. The sequence data used in this study are listed in Table 1.

**Table 1:** **RNA**-**seq datasets used in this study**

**Server**	**Dataset ID**	**Description**	**Download host**
SRA	SRP010181	Colorectal cancer tumor matched normal libraries (n = 130)	[29]
CGhub	Various	Twenty-five different cancers and normal tissues (n = 7,677) from TCGA	[30]
GEO	GSE40419	Lung adenocarcinoma tumor matched normal libraries (n = 144)	[31]
GEO	GSE30611	Human Bodymap 2.0 representing 16 normal human tissues	[31]
ENCODE	Various	Fastq files from 15 different cell lines, replicates downloaded where available	[32]
SRA	SRP008280	Triplicate control K562 cell RNA-seq	[29]

GEO, Gene Expression Omnibus; SRA, Sequence Read Archive; TCGA The Cancer Genome Atlas.

### Ethics

The research described herein conformed to the Helsinki Declaration. All clinical specimens were obtained previously [33] with informed consent by the BC Cancer Agency Tumor Tissue Repository (BCCA-TTR), which operates as a dedicated biobank with approval from the University of British Columbia-British Columbia Cancer Agency Research Ethics Board (BCCA REB; certificate #H09-01268).

### RNA-seq read mapping

Raw sequence reads were aligned to the human reference genome and transcriptome (hg19, Ensembl v.70) using STAR v.2.3.0e [34] or TopHat v.2.0.6 [35]. STAR was run with the following parameters: minimum/maximum intron size set to 30 and 500,000, respectively, noncanonical, unannotated junctions were removed, maximum tolerated mismatches was set to 10, and the outSAMstrandField intronMotif option was enabled. For TopHat reads aligned to human ribosomal RNA sequences (18S, 28S, 5S) were removed, and the remaining reads were aligned to human gene annotations (hg19, Ensembl v.70) using default command line options except the minimum isoform fraction option was set to 0. The Cuffdiff command included with Cufflinks v.2.0.2 [36] was used to calculate the fragments per kilobase of exon per million fragments mapped (FPKM) with upper quartile normalization, fragment bias correction, and multiread correction enabled. All other options were set to default.

### Targeted read assembly (TASR)

Targeted read assembly was performed using TASR v.1.5.1 with the following parameters enabled: -m 20 -o 2 -i 1 [37]. Briefly, the 397-nucleotide *EVADR* cDNA sequence (ENST00000418403) was used as the target for TASR, which extracted overlapping *k-mers* (-k 15) from this sequence as seeds. These were used to recruit *EVADR* RNA-seq reads from The Cancer Genome Atlas (TCGA) bam files, and the set of reads recruited from each sample was independently assembled *de novo.* Sequence contigs of 200 nucleotides and larger were aligned to the *EVADR* reference sequence with BLAST (v.2.2.22; parameters -a 8 -F F -p blastn -m 7), keeping only assembled transcripts with 90% or higher sequence identity [38]. The number of sequence reads assembled was tallied in each TCGA sample and RPK (reads per kilobase) and RPKMS (reads per kilobase per million sequenced) values were calculated [39].

### RT-PCR

RNA and cDNA were generated as described [40]. In this study, cDNA from tumor or normal tissues was used as a template for PCR using primers oHL_0005 and oHL_0006 (Table 2). The reaction was performed for 35 cycles with *Taq* DNA polymerase (NEB, Ontario, Canada) in supplied buffer and amplicons were resolved on a 1% agarose gel.

**Table 2:** **Primer sequences**

**Oligo**	**Purpose**	**Sequence (5′-3′)**
oHL_0005	Forward RT-PCR primers for *EVADR* transcript	TGATGCCATTTTCAGCCTCAG
oHL_0006	Reverse RT-PCR primers for *EVADR* transcript	TGGCCGCTCAGATTCTCTATC
oHL_0011	Clone MER48 LTR upstream of *EVADR*. XhoI/HindIII	GGCTCGAGTAAGGGAATGAATAACTCCG
oHL_0012	Clone MER48 LTR upstream of *EVADR*. XhoI/HindIII	GGCTCGAGATATGTACCCTGTGAAGACC
oHL_0015	Cloning small fragment of MER48 element	GGCTCGAGGTGCTGATGCCATTTTCAGCC
oHL_0013	Clone MER48 LTR upstream of *EVADR*. XhoI/HindIII	GGAACCTTACATGCTGTTTTAATGAGCG
oHL_0016	Same as oHL_0011 but reversed restriction site. XhoI	CCAAGGTTTAAGGGAATGAATAACTCCG
oHL_0017	Same as oHL_0012 but reversed restriction site. HindIII	GGCTCGAGACATGCTGTTTTAATGAGCG
oHL_0014	Same primer as oHL_0006 but for cloning 5′RACE products	CCGGATCCTGGCCGCTCAGATTCTCTATC

### Cell culture

K562 and SW480 cells were cultured in Dulbecco's modified Eagle's medium (DMEM) supplemented with 10% fetal bovine serum and penicillin (100 units/ml)-streptomycin (100 μg/ml) (Life Technologies, Ontario, Canada). Cells were maintained at 37°C in a 5% CO_2_ incubator.

### Plasmids

The MER48 promoter deletion plasmid constructs were generated by first amplifying the MER48 element specific to *EVADR* using primers oHL_0011 and oHL_0005. The subsequent amplicon was gel purified, diluted 1:100 and used as a template for amplifying each truncated version of the MER48 element: MER1F, oHL_0011 and oHL_0013; MER2F, oHL_0012 and oHL_0013; MER3F, oHL_0015 and oHL_0013; and MER_FLIP, oHL_0016 and oHL_0013. Amplicons were digested with XhoI and HindlII, gel purified and ligated into the pGL4 vector (Promega, Wisconsin, United States).

### Reporter assays

K562 cells were suspended in Electroporation Buffer (20 mM HEPES, 137 mM NaCl, 5 mM KCl, 0.7 mM Na_2_HPO_4_, 6 mM dextrose, 50 mM trehalose, 1% DMSO) with 30 μg plasmid and electroporated at 200 V, 975 μF capacitance. SW480 cells were grown to 60% confluency and then reverse transfected using Lipofectamine 2000 (Life Technologies, Ontario, Canada) according to the manufacturer’s instructions. The cells were harvested 24 h after electroporation (for K562) or 48 h after transfection (for SW480), and firefly and *Renilla* luciferase activities were analyzed with the Dual-Luciferase® Reporter Assay System, according to the manufacturer’s instructions (Promega, Wisconsin, United States). All experiments were repeated in triplicate.

### RNA ligase-mediated rapid amplification of cDNA ends

5′ Rapid amplification of cDNA ends (RACE) was performed with a RLM-RACE kit (Life Technologies, Ontario Canada) according to the manufacturer’s instructions using total RNA isolated from K562 cells and the reverse primer oHL_0014.

### MER48 expression patterns from K562 cell line data

A list of 201 reliable MER48 coordinates was obtained from Dfam [41]. This list was used to count reads aligning to MER48 elements from two replicate K562 transcriptome .bam files obtained from ENCODE using readcount v0.01 with all options set to default.

### Statistical tests

All statistical tests were performed using R v.3.1.0.

#### Tumor and normal expression comparisons

For each of the colorectal and lung cancer datasets, we compared the difference between mean tumour expression values and mean normal tissue expression values using a two-sample paired *t*-test.

#### STAR/Cufflinks and TASR expression correlations

The Pearson correlation coefficient was used to quantify the strength of the correlations between the STAR/Cufflinks- and TASR-generated expression levels.

#### *EVADR* tumor site association

All patients for each cancer type were classified according to whether *EVADR* was detectable (>0 RPKMS). Next, we counted the total number of patients in the two groups (adenocarcinoma and non-adenocarcinoma), with and without detectable *EVADR* expression. Using these counts, we performed a Chi-squared test to test the significance of the observed association of *EVADR* expression and adenocarcinoma.

#### Reporter assays

The dual luciferase promoter mapping data were subjected to *t*-tests with unequal variance followed by Bonferroni correction, comparing the mean relative luciferase units (RLU) of the MER1F construct with the RLUs of each of the MER2F, MER3F and MER_FLIP constructs, in each of the K562 and SW480 cell lines.

#### Correlation of MER48-lncRNA expression

For each cancer type we performed an ANOVA test to test the null hypothesis that the expression of all MER48-lncRNAs were the same. A significant result from ANOVA was obtained for each of the five cancer types, which prompted a subsequent Tukey’s honest significant difference *post hoc* test to identify which MER48-lncRNAs were differentially expressed in each cancer type.

#### Structural conservation

Using the program Molecular Evolutionary Genetics Analysis (MEGA) v.6 [42], we inferred the evolutionary history of the primates using the maximum parsimony method and *EVADR* cDNA alignments. The resultant tree (Figure S1 in Additional file 1) was used to count the number of nucleotide changes in context of *EVADR* evolution in primates. To test the differences in the number of variable positions in base-paired sequences (structured regions) compared with non-structured sequences, we used a Chi-squared test to compare the count of sites in structured and non-structured sequence classes lacking perfect sequence conservation across all studied species. To test whether the observed nucleotide substitutions in structured regions were more likely to maintain base pairing than expected by chance, we used a Chi-squared test to compare the observed distribution with the distribution expected by random substitutions. To create this null distribution, we tallied for GC, GU and AU base pairs all possible random nucleotide substitutions (eight per pair, including single nucleotide deletions). Of the 24 possible combinations, four nucleotides are expected to still base pair after random mutation.

### Sequence identity calculations

Genomic alignments were generated using ClustalW and imported into Jalview2 [43] for manual curation. The polished alignments were exported as a ClustalW alignment file and uploaded to the SIAS [44] website to determine the sequence identity of *EVADR* between primates. We included parameters to take gaps into account and used the BLOSUM62 matrix; all other settings were set to default. The tree was generated using the UCSC genome tool phlyoGif [45,46].

### Coding potential analysis

To confirm that *EVADR* was non-coding, we used the online coding potential calculator tool (CPC) to calculate *EVADR*’s coding potential [47]. As a positive control, we included the cancer-associated lncRNA *UCA1*. The results of this analysis were both transcripts were considered to be non-coding, with a CPC score of -1.29 for *EVADR* and -1.14 for *UCA1*, where values between -1 and 1 are marked as ‘weak non-coding’ or ‘weak coding’, respectively. Non-coding RNAs score below -1 and protein coding genes score above 1.

Using the online tool ORF Finder [48], we identified three putative open reading frames (ORFs) within *EVADR*, including one ORF with a non-AUG start codon (Figure S2 in Additional file 1). To determine whether these ORFs were actively translated, we queried several K562 proteomic datasets from studies that specifically evaluated the presence of small peptides and found no evidence for translation of the *EVADR* ORFs [49-51]. These findings further support the classification of *EVADR* as a lncRNA with little to no apparent translation.

### Heatmaps

The MER48-lncRNA heatmaps were clustered using centroid linkage and Euclidean distance in custom Python scripts.

### Genome mining MER48-associated lncRNAs

Dfam was used to identify MER48 elements within 500 bp or 50 bp of a predicted lncRNA transcriptional start site using Ensembl v.70 annotations. We identified nine candidates using 500 bp and eight using 50 bp; in both cases *EVADR* was included. Manual examination of the excluded lncRNA ENSG00000265374 revealed the MER48 formed part of the exon of the lncRNA, but the transcriptional start site was upstream of the repeat element. We included this lncRNA in our analyses.

## Results

### Identification of a highly upregulated long non-coding RNA in colorectal carcinoma

**Figure 1:**
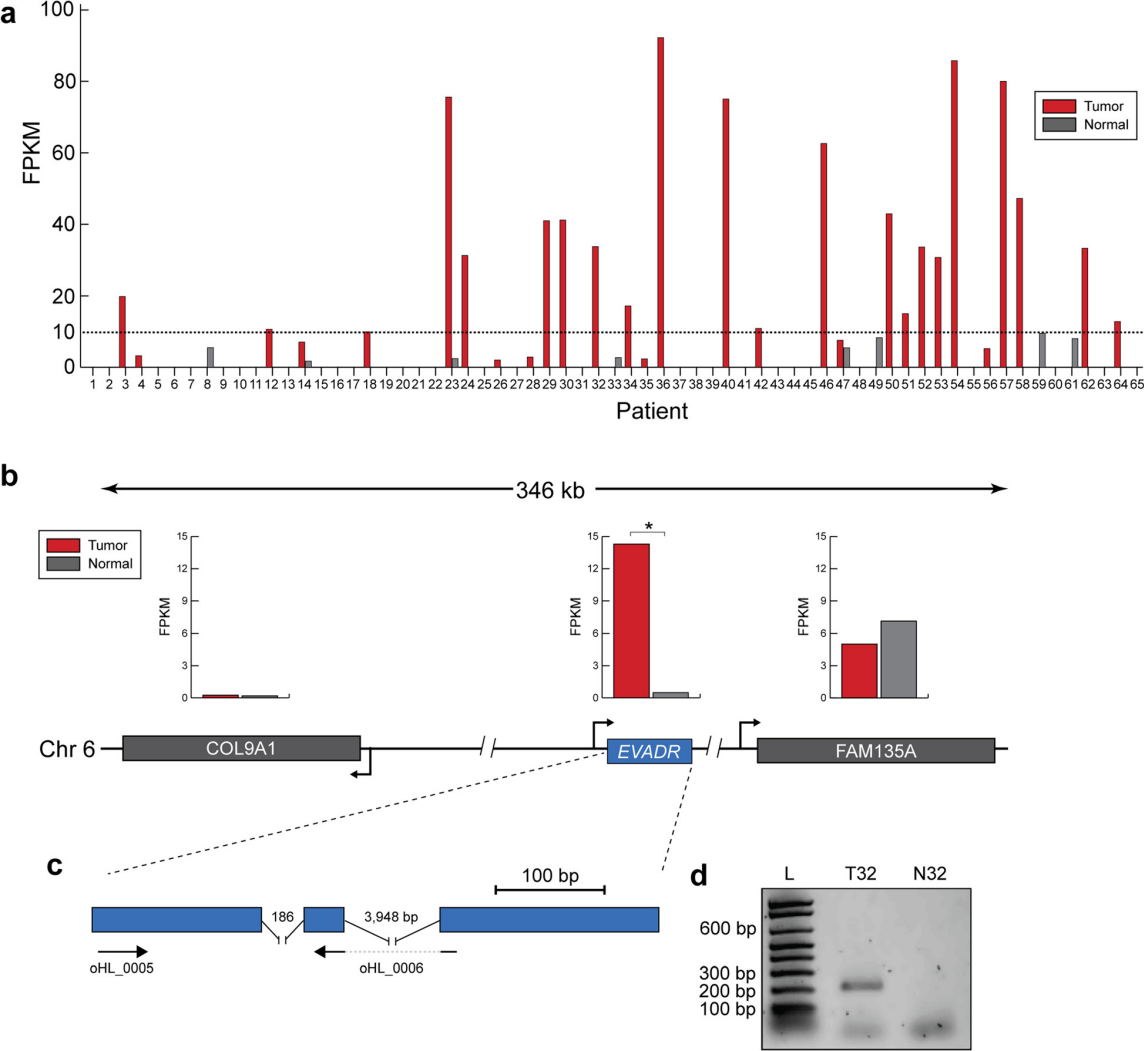
**A long non-coding RNA is highly activated in colorectal tumors. (a)** Expression of *EVADR* in tumor and adjacent normal tissue from 65 patients with colorectal carcinoma. For each subject (x-axis) tumor is shown in red and normal tissue in grey. The dashed line indicates an arbitrary minimum expression threshold of 10 FPKM. A total of 21 patients demonstrated robust (>10 FPKM) levels of *EVADR* expression. Normal colon tissue was generally not found to express *EVADR* or it was expressed at low levels (<10 FPKM). **(b)** Schematic representation of the chromosomal location of the *EVADR* gene locus. Arrowheads indicate the orientation of transcription. Bar plots indicate mean expression levels for respective genes across all 65 tumor (*COL91A*, 0.25 ± 1.11 FPKM; *EVADR*, 14.4 ± 24.5 FPKM; *FAM135A*, 7.2 ± 3 FPKM (mean ± SD)) and normal samples (*COL91A*, 0.19 ± 0.53 FPKM; *EVADR*, 0.50 ± 1.52 FPKM; *FAM135A*, 5 ± 2.6 FPKM (mean ± SD)). *P < 0.00001; paired *t*-test. **(c)** Exon structure and primer locations for the lncRNA *EVADR*. **(d)** Representative gel image showing *EVADR* transcript levels in colorectal tumor (T32) and matched normal (N32) tissue samples for patient 32, measured by RT-PCR. The letter L indicates the molecular ladder.

To identify lncRNAs associated with colorectal cancer, we performed whole transcriptome sequence analysis on 65 tumor and matched normal colorectal poly(A)-selected RNA-seq libraries generated in a previously described study [33]. From these data we identified two Ensembl-curated yet uncharacterized lncRNAs, ENSG00000222041 and ENSG00000237643, which were strongly upregulated in colorectal tumors compared with matched normal control tissues. We did not characterize ENSG00000222041 further because its differential expression was less substantial than that of ENSG00000237643, and because it had a complex alternative splicing pattern that complicated further analysis. Further scrutiny of ENSG00000237643 (which we named *EVADR* for Endogenous retroViral-associated ADenocarcinoma RNA) revealed significantly increased expression of this lncRNA in tumors (14.4 ± 24.5 FPKM (mean ± standard deviation (SD))) compared with matched normal control tissues, where it was typically not expressed (0.50 ± 1.52 FPKM (mean ± SD)) (Figure 1a; *P* = 2.8e-05; *t*-test). Genomic analysis revealed a predicted 397-nucleotide lncRNA with three exons and a single transcript isoform located on chromosome 6, 91.8 kb downstream of *Col9A1* and 13.5 kb upstream of *FAM135A* (Figure 1b). Despite high expression of *EVADR* in tumor samples, we did not observe differential expression of genes flanking *EVADR* (Figure 1b). We designed primers to amplify a 200 bp region of the mature *EVADR* transcript and validated the expression of this lncRNA in the colorectal tumor and normal tissues using RT-PCR (Figure 1c,d; Additional file 2). To corroborate our observation that *EVADR* was not expressed in non-malignant tissue, we measured *EVADR* expression in 16 normal adult human tissues finding only weak expression in lung and prostate tissues (Figure S3A in Additional file 1). Expanding these analyses to 15 cell lines, we found that *EVADR* was highly expressed (122 FPKM) in the chronic myeloid leukemia K562 cell line, but its expression was extremely low (<1 FPKM) in the others, including in the H1-HESC embryonic stem cell line (Figure S3B in Additional file 1), where it was 0.04 FPKM. These data demonstrate that *EVADR* is selectively expressed.

### Pan-cancer analysis reveals high activation of *EVADR* in human adenocarcinoma

**Figure 2:**
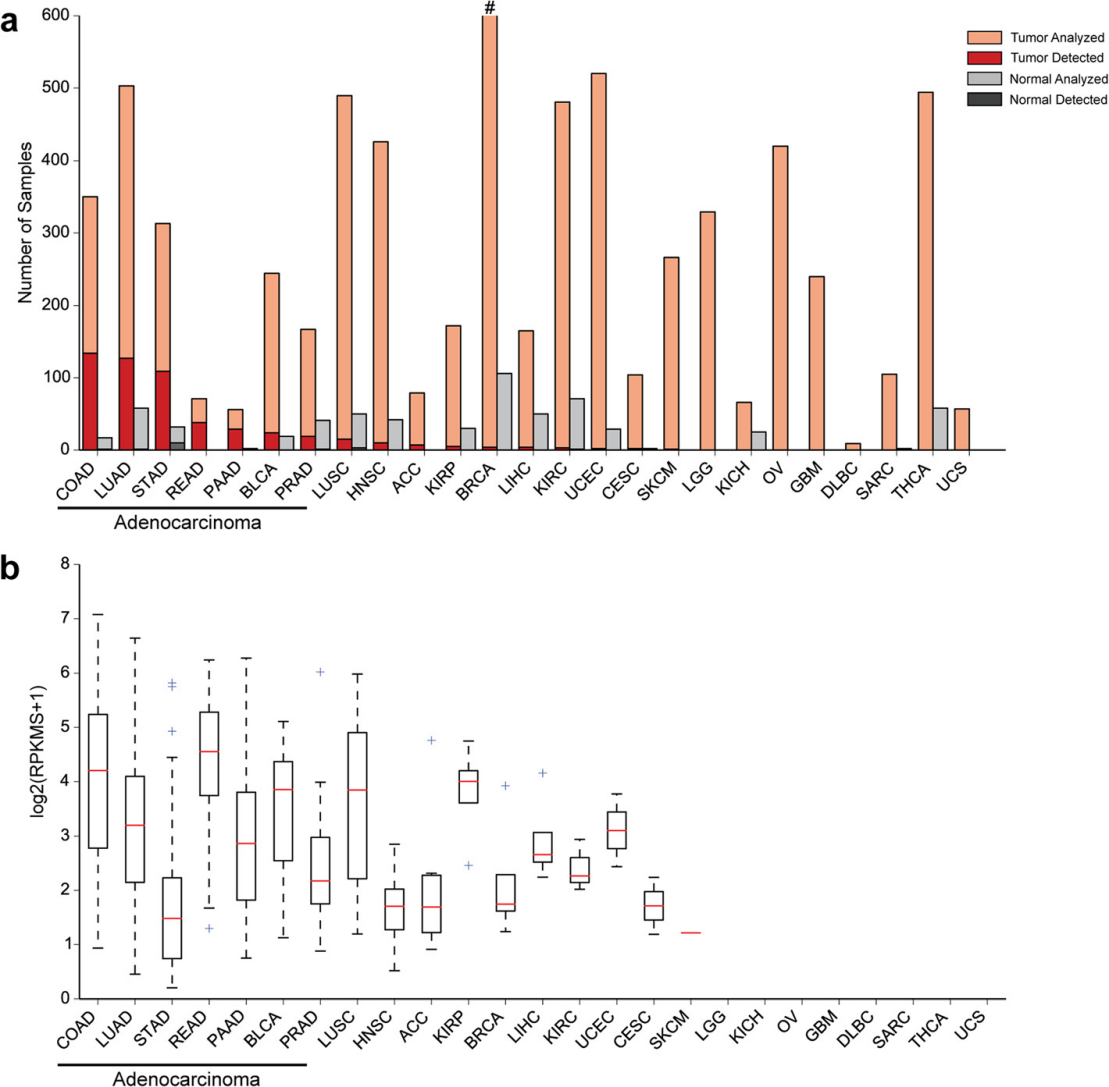
***EVADR*** **is robustly expressed in adenocarcinomas. (a)** *EVADR* expression in 25 TCGA cancer types and corresponding normal tissues. Light orange indicates the tumors analyzed and light grey indicates normal samples analyzed. The hashtag (#) indicates that 916 BRCA samples were analyzed. Dark red indicates the number of adenocarcinoma samples in which *EVADR* expression was detected, while dark grey indicates the number of normal samples in which *EVADR* was detected. **(b)** *EVADR* expression as log_2_(RPKMS + 1), determined for tumors using TASR. Medians are indicated by red lines, upper and lower quartiles by the boxes, and outliers by blue crosses. COAD, colon adenocarcinoma; LUAD, lung adenocarcinoma; STAD, stomach adenocarcinoma; READ, rectum adenocarcinoma; PAAD, pancreatic adenocarcinoma; BLCA, bladder urothelial carcinoma; PRAD, prostate adenocarcinoma; LUSC, lung squamous cell carcinoma; HNSC, head and neck squamous cell carcinoma; ACC, adrenocortical carcinoma; KIRP, kidney renal papillary cell carcinoma; BRCA, breast invasive carcinoma; LIHC, liver hepatocellular carcinoma; KIRC, kidney renal clear cell carcinoma; UCEC, uterine corpus endometrial carcinoma; CESC, cervical squamous cell carcinoma and endocervical adenocarcinoma; SKCM, skin cutaneous melanoma; LGG, brain lower grade glioma; KICH, kidney chromophobe; OV, ovarian serous cystadenocarcinoma; GBM, glioblastoma multiforme; DLBC, lymphoid neoplasm diffuse large B-cell lymphoma; SARC, sarcoma; THCA, thyroid carcinoma; UCS, uterine carcinosarcoma.

The striking expression pattern observed for *EVADR* in colorectal carcinoma prompted us to perform additional analysis across a broad panel of human cancers. We queried *EVADR* expression in all available tumor (n = 7,043) and normal (n = 634) RNA-seq libraries from TCGA Research Network project. Because full transcriptome assembly requires significant compute resources we opted to analyze TCGA RNA-seq data specifically for *EVADR* expression using the targeted *de novo* assembler TASR [37], which is much faster in situations where transcriptome-wide information is not required. To convert TASR-derived assemblies to expression values, we tallied assembled sequence reads and calculated the RPKMS. To determine the concordance between Cufflinks-derived FPKMs and TASR-derived RPKMS we compared *EVADR* expression across 181 colon adenocarcinoma (COAD) samples and observed a Pearson correlation of r = 0.93. Next, we used TASR to quantify *EVADR* expression in 7,677 tumor and normal TCGA RNA-seq datasets (Figure 2a). Consistent with our initial observation that *EVADR* was specifically upregulated in colorectal tumors, we found that this lncRNA was detected in TCGA colon and rectal tumor datasets at high (mean 25.3 ± 22.7 RPKMS (mean ± SD)) expression levels (Figure 2a,b). Strikingly, we also found high *EVADR* expression in numerous lung (LUAD), pancreatic (PAAD), and stomach (STAD) adenocarcinomas (12.3 ± 13.8, 12.1 ± 17.2, and 3.7 ± 7.7 RPKMS, respectively; Figure 2a,b; Figure S4 in Additional file 1). We found that while 481 out of 1,223 adenocarcinomas expressed *EVADR* at detectable levels, only 50 out of 5,289 non-adenocarcinomas expressed *EVADR*. We performed a Pearson’s Chi-squared test comparing these two groups and found that *EVADR* was significantly (*P* < 2.2e-16) associated with adenocarcinomas, but not with the other tumor types. Having confirmed high *EVADR* expression in the colorectal tumors in two independent datasets, we also validated the cancer association of *EVADR* in a non-colorectal tumor and matched normal control tissue dataset. We processed an independent lung adenocarcinoma transcriptome dataset [52] using Cufflinks [36], and observed high *EVADR* expression in the tumors (9.84 ± 24.7 FPKM (mean ± SD)) and weak or undetectable expression in normal tissues (mean 0.44 ± 0.55 FPKM (mean ± SD)) (*P* = 0.002; *t*-test; Figure S5 in Additional file 1). These data were consistent with the high *EVADR* expression in lung adenocarcinoma observed in the TCGA samples.

### Clinical features of *EVADR* track with poor prognosis in adenocarcinomas

**Figure 3:**
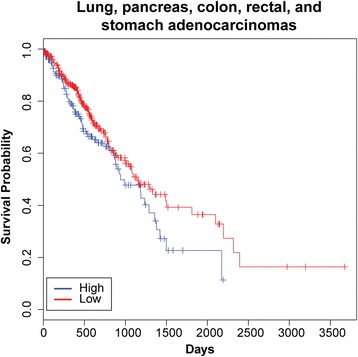
**Overall survival decreases for adenocarcinoma patients expressing** ***EVADR*** **.** Kaplan-Meier curves were constructed using a univariate Cox analysis, stratifying patients based on *EVADR* levels in tumor, with patients expressing high (>5.6 FPKM) levels of *EVADR* showing decreased survival when compared with those with low (<5.6 FPKM) expression (hazard ratio = 1.47, 95% confidence interval = 1.06 to 2.04, *P* = 0.02). Tick marks on the graph denote the last time survival status was known for living patients.

Having observed specific and high-level expression of *EVADR* in the tumors of patients with lung, colon, rectal, stomach and pancreatic adenocarcinoma, we sought to identify any possible clinical relevance of the association of *EVADR* with these tumor types. To explore this possibility, we selected TCGA datasets for these five adenocarcinoma tumor types because of their large patient cohorts and the availability of corresponding clinical data. Using a Cox proportional-hazard model that accounted for known prognostic factors (age, gender, type of adenocarcinoma, and tumor stage) as well as *EVADR* expression (>5.6 FPKM; threshold determined using Cutoff Finder [53]), we saw a significantly (*P* < 0.02) decreased overall survival for patients with high *EVADR* expression (Figure 3). These data associate elevated *EVADR* expression with lower overall survival for the five TCGA tumor types investigated.

### An ERV LTR contributes a functional promoter to *EVADR*

**Figure 4:**
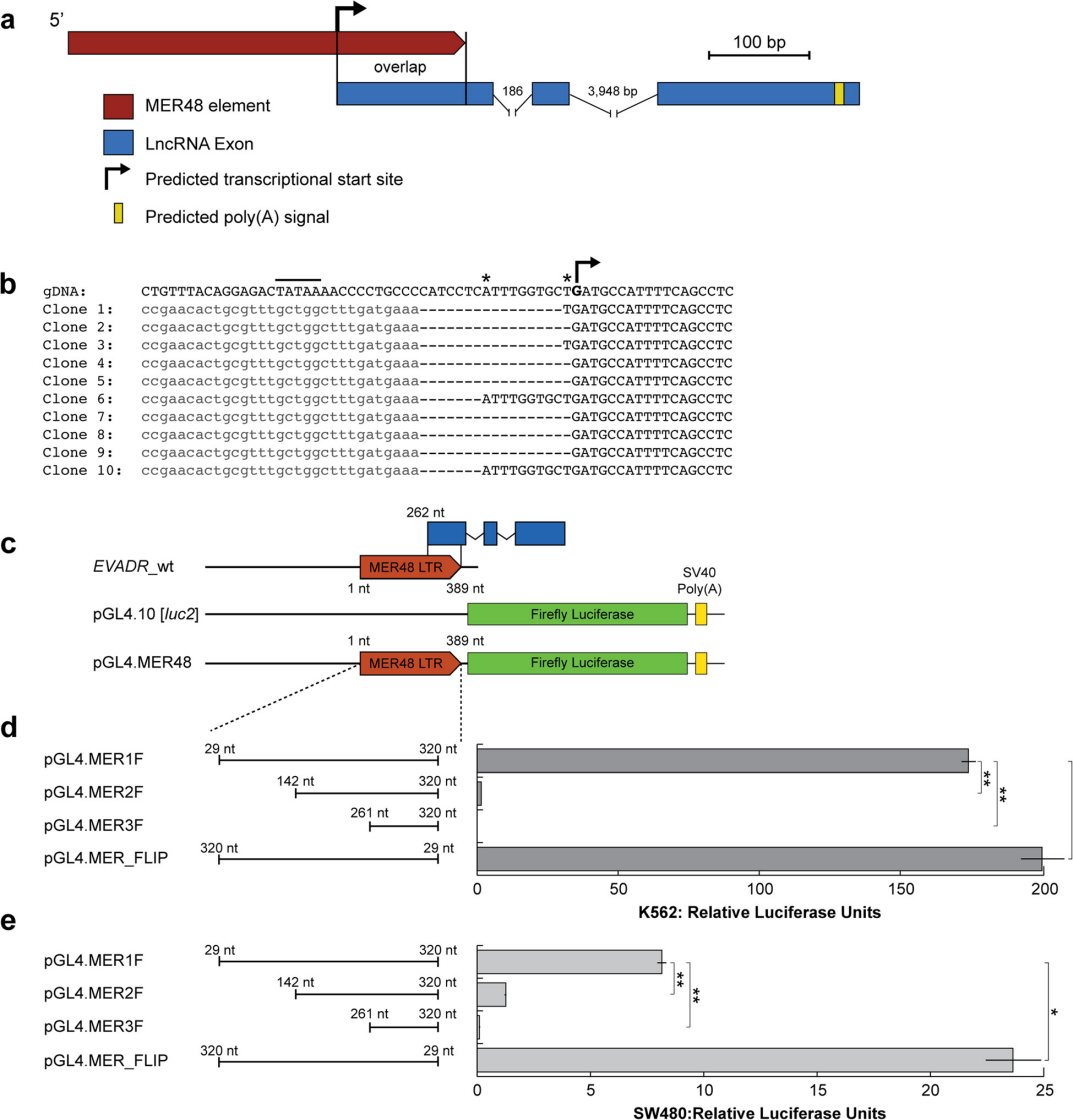
**The lncRNA** ***EVADR*** **is partially derived from a MER48 ERV element. (a)** Gene structure indicating the MER48 element overlapping with the 5′ termini of *EVADR* (red); lncRNA exons are shown in blue, predicted poly(A) signal in yellow and the promoter by a bent arrowhead. **(b)** Partial sequences of 10 clones from 5′ RNA ligase-mediated RACE analysis of MER48 initiated *EVADR* transcripts aligned to human genomic DNA. The predicted TATA box is indicated by a line, the minor transcriptional start sites by an asterisk, and the predominant initiating nucleotide is bolded and indicated by a bent arrow. The RNA adaptor sequences are light grey and in lower case. **(c)** Promoter deletion experimental design showing truncations of the MER48 element. The MER48 LTR is indicated by a red arrow, the luciferase ORF by green rectangles, and *EVADR* is indicated by blue rectangles. **(d)** Results of the promoter analysis in K562 cells. **(e)** Results of the promoter analysis in SW480 cells. *Adjusted *P* < 0.05; **adjusted *P* < 0.005; two-sample *t*-test with Bonferroni correction.

Detailed sequence analysis of the *EVADR* genomic locus revealed sequence identity with a MER48 LTR, which is an endogenous retroviral element of the ERV1 family (Figure 4a) [54]. The MER48 LTR contributes 127 nucleotides to the primary sequence of the 5′ exon of *EVADR*, and also encodes numerous transcription factor binding sites and a putative TATA box, suggesting a possible role for these regulatory sequences in the transcriptional activation of *EVADR* in adenocarcinoma. As K562 cells strongly express *EVADR*, we selected these cells for 5′-RACE [55] and mapped three distinct 5′ transcript termini to the *EVADR* MER48 LTR, each downstream of the predicted TATA box (Figure 4b). These data refine the length of the predominant *EVADR* transcript from the predicted value of 397 nucleotides to the confirmed value of 394 nucleotides. To experimentally test the capacity of the MER48 LTR in driving downstream transcription, we generated a series of truncated MER48 constructs and measured promoter activity, in triplicate, using a dual luciferase assay (Figure 4c). While full-length MER48 is active in both K562 and SW480 cells (MER1F; K562, 73.7 ± 5.1 RLU (mean ± SD); SW480 8.14 ± 0.40 RLU), each subsequent 5′ truncation dramatically reduced luciferase activity (MER2F; K562, mean 1.45 ± 0.14 RLU; SW480, 1.26 ± 0.08 RLU), with negligible promoter activity observed for MER48 sequence overlapping with the 5′ exon of *EVADR* (MER3F; K562, 0.08 ± 0.01 RLU; SW480, 0.10 ± 0.01 RLU) (Figure 4d,e). Surprisingly, we observed strong luciferase activity when the *EVADR* MER48 LTR was in the opposite orientation (MER_FLIP; K562, 198 ± 15.4 RLU; SW480, 23.6 ± 2.4 RLU), indicating the MER48 LTR can function as a bidirectional promoter (Figure 4d,e; MER_FLIP). Despite the high transcriptional activity observed from the MER_FLIP plasmid constructs, we found no evidence supporting bidirectional transcription *in vivo* (Figure S6 in Additional file 1). These results demonstrate that the MER48 LTR not only contributes to the 5′ exon of *EVADR*, but also provides a promoter sequence capable of driving the transcription of this lncRNA.

### *EVADR* is selectively upregulated in adenocarcinomas

**Figure 5:**
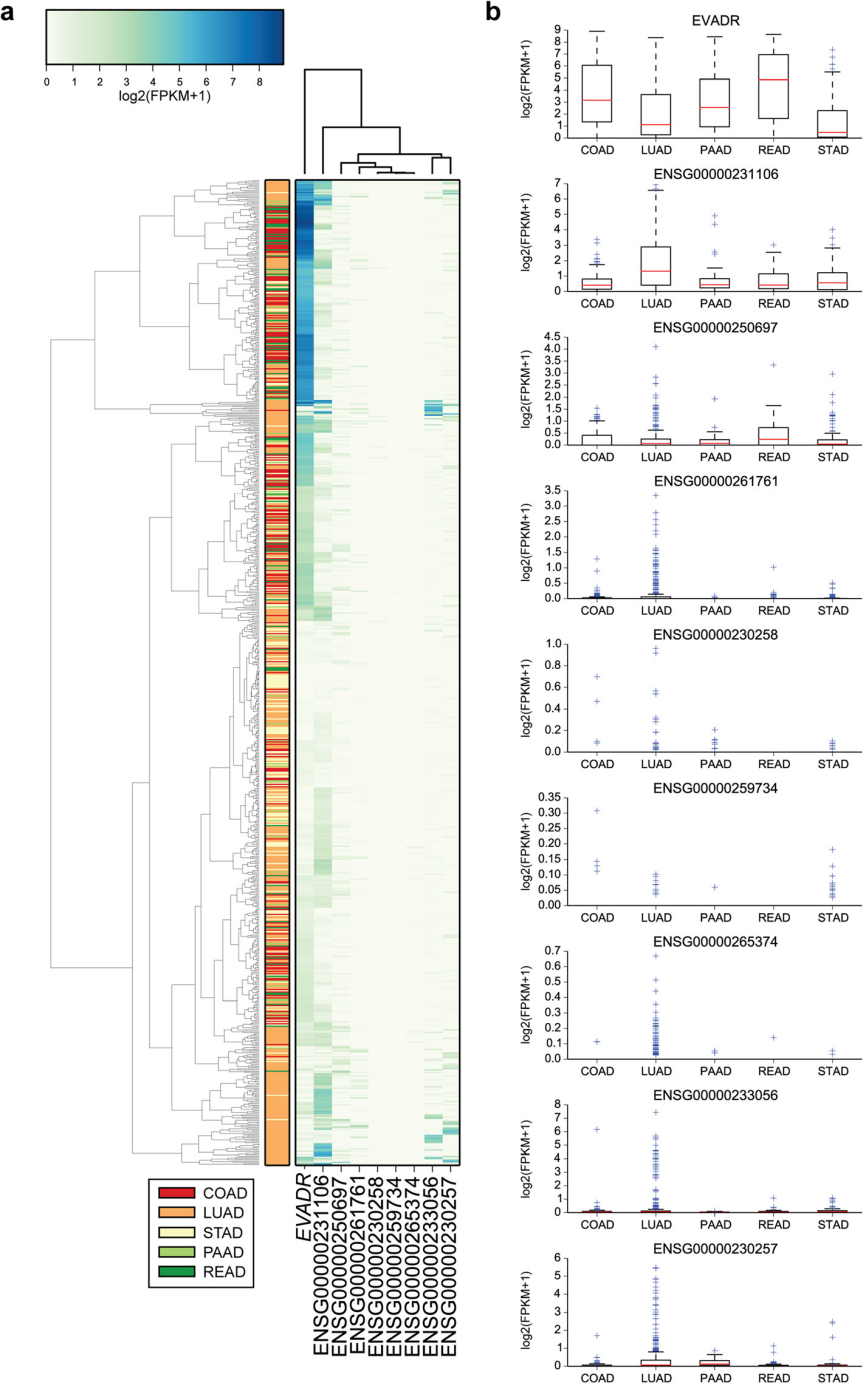
**MER48 activation is specific, rather than general, in adenocarcinoma tumors. (a)** Clustered heatmap showing the expression of nine MER48-associated lncRNAs in a panel of colon (COAD; n = 181), rectal (READ; n = 66), pancreatic (PAAD; n = 52), lung (LUAD; n = 372) and stomach (STAD; n = 136) adenocarcinomas. **(b)** Expression levels for the same nine MER48-lncRNAs. The y-axis is log_2_(FPKM + 1) for each set. Medians are indicated by red lines, upper and lower quartiles by boxes, and outliers by blue crosses.

**Figure 6:**
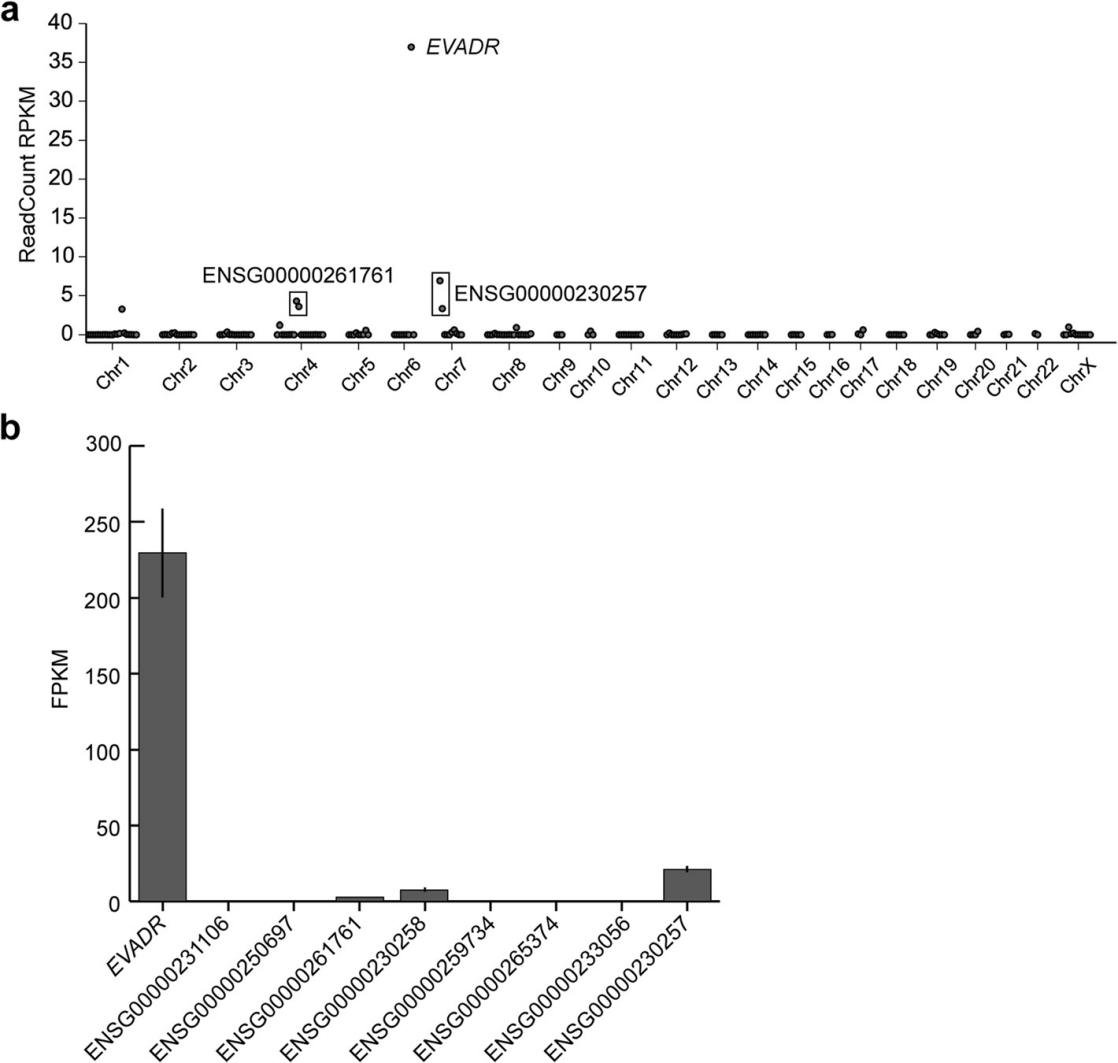
**MER48 LTRs are not globally active in K562 cells. (a)** Scatterplot of expression for 201 reliable MER48 elements in K562, with *EVADR* being the highest expressed MER48 element. Plotted values are the average of two experiments. **(b)** Expression of the nine MER48-lncRNAs in K562s in a validation dataset. Values are the average of three experiments. The ENSG00000230257 is driven by a MER48 element flanking a HERVH48 insertion. The ENSG00000261761 lncRNA MER48 is split by an Alu insertion. The lncRNA ENSG00000230258 is associated with an unreliable MER48 in Dfam [41] and was not, therefore, part of the list used for the scatterplot and does not appear in these data.

A MER48 LTR is found within 500 bp of the annotated transcriptional start sites of nine Ensembl annotated lncRNAs. To determine if *EVADR* expression is due to a general activation of MER48 LTR elements in adenocarcinomas, we queried the expression of the other eight non-*EVADR* MER48-associated lncRNAs in colon, rectal, pancreas, stomach and lung tumors using Cufflinks [36]. These tumor types were selected because they included large numbers of patients that expressed *EVADR* at high levels. *EVADR* clustered independently of other MER48-lncRNAs and of tumor type (Figure 5a). Next, we determined the distribution of lncRNA expression for all MER48-lncRNAs in each of the five different types of adenocarcinoma, and for each type we observed significantly elevated expression of *EVADR* relative to all other MER48-lncRNAs (*P* < 2e-16 for each cancer type; ANOVA with Tukey’s honest significant difference *post hoc* test; Figure 5b). Additionally, in lung adenocarcinoma, ENSG00000231106 is also expressed differently from all other MER48-lncRNAs (including *EVADR*). Finally, to determine whether MER48 LTRs were universally active, we queried the expression of 201 Dfam-curated MER48 LTRs in K562 cells and found that the MER48 LTR associated with *EVADR* is specifically and highly activated, while the remaining MER48 elements were inactive or showed only minimal expression (Figure 6a). To validate these data, we examined an additional K562 RNA-seq dataset to quantify the MER48-lncRNAs using Cufflinks [36] (Figure 6b). The highest expressed MER48-lncRNA in this dataset was *EVADR* (230 FPKM), consistent with the read count-derived data in Figure 6a. The lncRNA ENSG00000230257 also showed moderately increased expression (21 FPKM) in K562 cells, while the expression of the other seven lncRNAs was low or undetectable (Figure 6b). Collectively, these results demonstrate that *EVADR* is specifically activated and not due to global MER48 activation.

### *EVADR* is a highly conserved, primate-specific lncRNA

**Figure 7:**
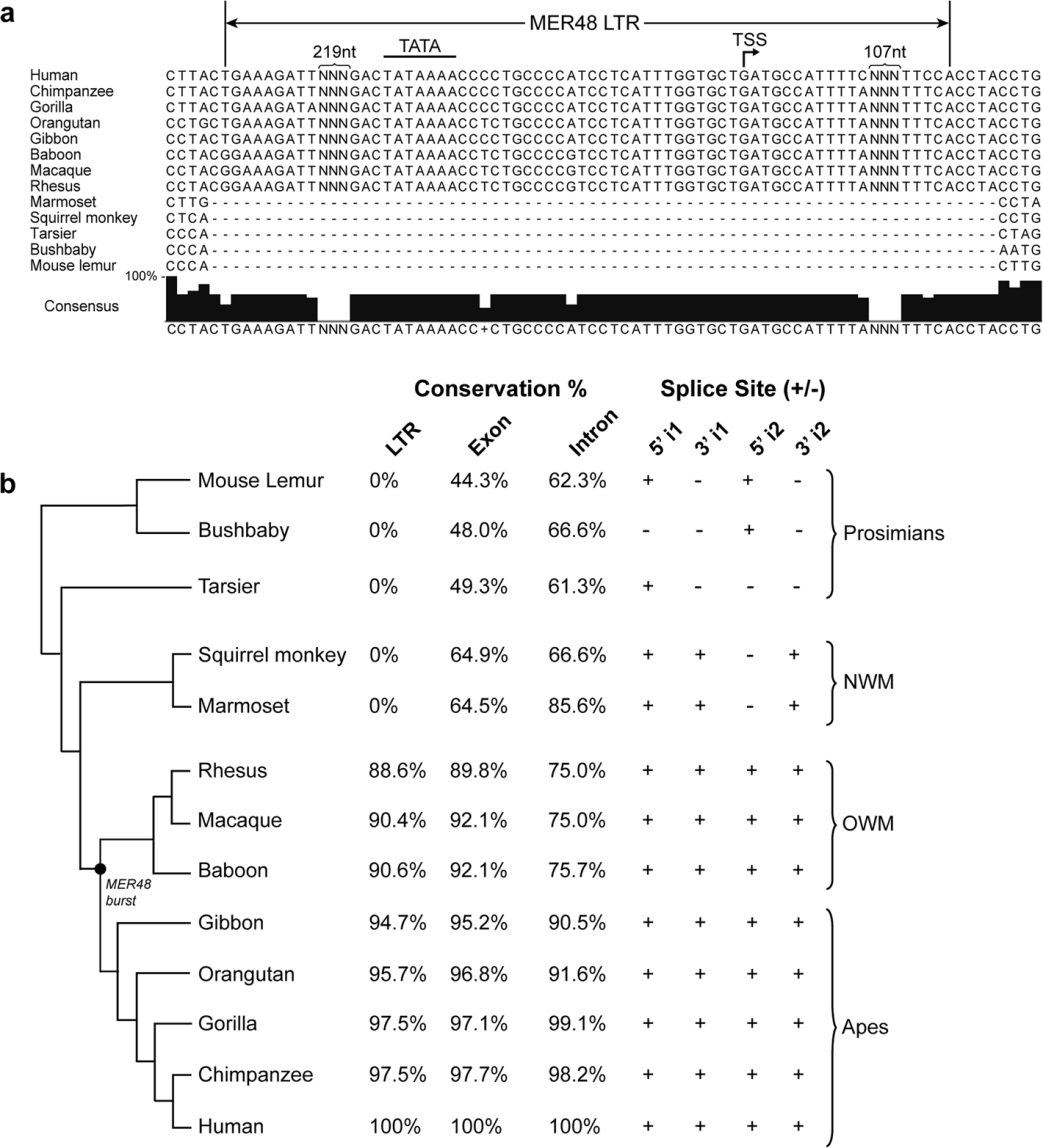
**Sequence conservation of the** ***EVADR*** **lncRNA in primates. (a)** Partial sequence alignment of the *EVADR* MER48 and flanking sequence in 13 primates showing lack of MER48 LTR in NWMs and prosimians. The major experimentally determined transcriptional start site (TSS) is indicated by a bent arrow, while the predicted TATA box is indicated by a line. Due to space constraints, some sequence has been removed and is indicated by NNN and curly brackets. **(b)** Sequence identity of the *EVADR* MER48 LTR and *EVADR* in 13 primate species determined with SIAS (Methods) on ClustalW aligned sequences. The first and second introns are indicated by an i1 and i2, respectively. The tree was generated using the UCSC genome tool phlyoGif [45,46]. The black dot indicates a burst of MER48 insertion as determined by the GEnome-wide Browser for RETroelement (GEBRET) webtool [56]. For GEBRET output and complete sequence alignments see Figure S9 in Additional file 1 and ClustalW alignments in Additional file 3, respectively.

**Figure 8:**
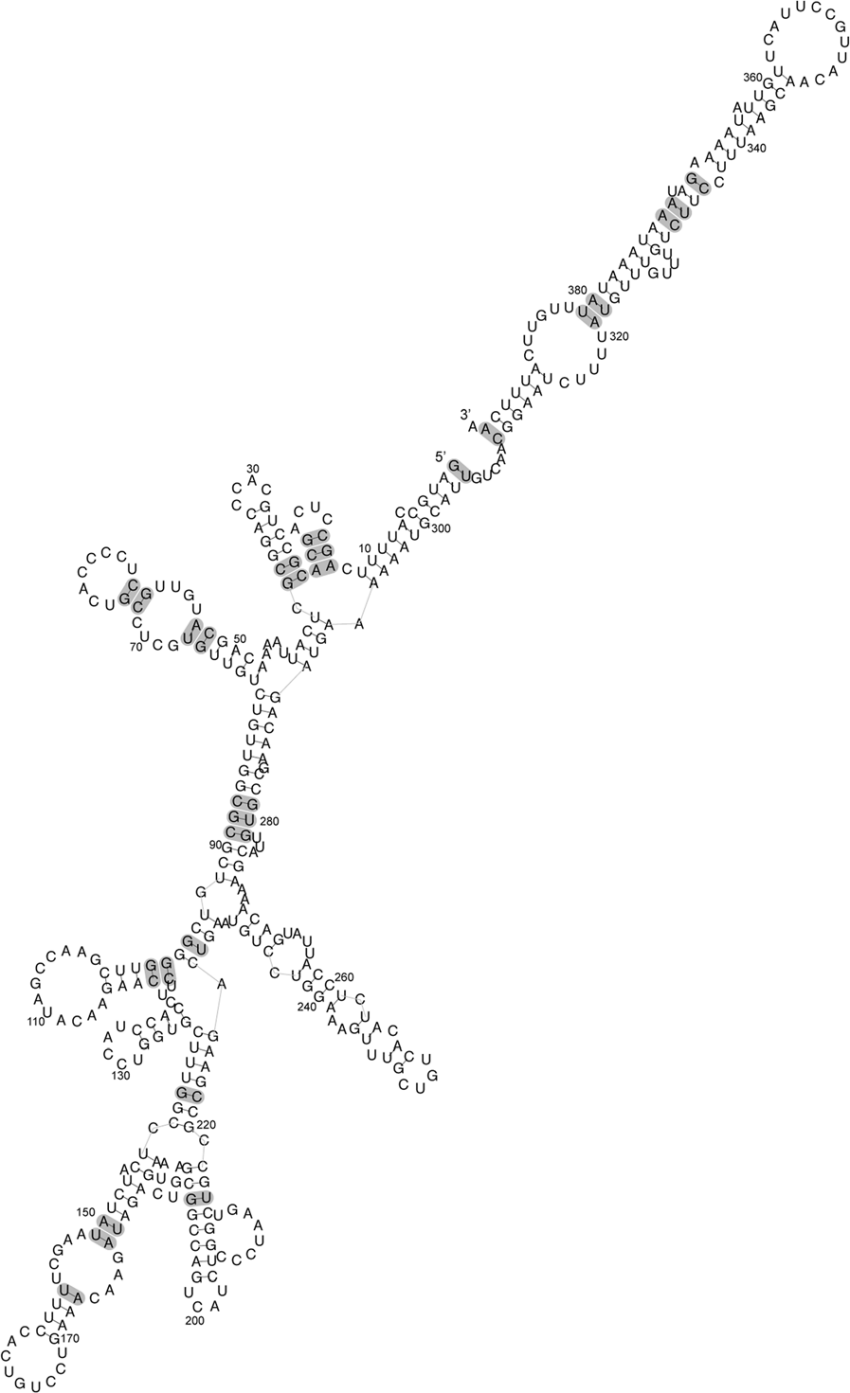
**Human** ***EVADR*** **secondary structure.** RNAalifold was used to predict a secondary structure based on a consensus fold for apes and OWMs [57]. Shading indicates the 27 base pair positions with nucleotide substitutions. The *EVADR* alignments showing all nucleotide substitutions and a chart showing only the base pair substitutions are shown in Figures S7 and S8 in Additional file 1, respectively.

Comparative sequence analysis of the *EVADR* MER48 LTR revealed high (>90%) sequence identity to the *EVADR* MER48 LTR in Old World monkeys (OWMs) and apes. Interestingly, however, the *EVADR* LTR is absent in more distant primates, including New World monkeys (NWMs) and prosimians (Figure 7a,b). The full 394 bp *EVADR* lncRNA gene demonstrated high sequence identity in apes and OWMs, particularly in the exons, with the introns showing decreasing identity with increasing phylogenetic distance (Figure 7b; Additional file 3). Both *EVADR* introns (i1 and i2) retained conserved GT/AG splice junctions in the apes and OWMs, but these junctions were not consistently conserved in NWMs or prosimians. The high nucleotide conservation for *EVADR* corresponds to an evolutionarily conserved secondary structure in OWMs and apes (Figure 8), with significantly more variable nucleotide positions occurring in unpaired or loop positions (47/152) than in structured regions (39/242 of paired nucleotide positions) (*P* = 0.0008; Chi-squared test; Figures S7 and S8 in Additional file 1). The 39 substitutions in structured regions affected a total of 32 base pairs at 27 locations, where 19 out of 39 substitutions were compensatory, maintaining base pairing and 20 out of 39 were non-compensatory, disrupting base pairing (Figure S8 in Additional file 1). Given 4 out of 24 random possible nucleotide changes in GC, GU and AU base pairs would maintain base pairing, we find that nucleotide substitutions in stems are significantly more likely to be compensatory (19/39 versus 7/39; observed versus expected) than non-compensatory (20/39 versus 33/39; observed versus expected) base pairs in stem regions (*P* = 7.8e-08, Chi-squared test). These results show the *EVADR* lncRNA has strong conservation in primates closest to humans, at both the primary and secondary structural levels.

## Discussion

We report the tumor-specific activation of *EVADR*, a novel lncRNA gene which is associated with a MER48 LTR. This lncRNA demonstrates nominal to low expression in normal tissue, but is significantly upregulated in cancer, particularly in colon, rectal, lung, stomach and pancreas adenocarcinomas.

Approximately 8% of the human genome is composed of ERV elements [58-60], with a number of ERV1, ERVL-MaLR, ERVL and ERVK elements overlapping a substantial number of lncRNAs [18]. The MER48 LTR is a member of the ERV1 class of retroviral elements, and is likely derived from an unknown retrovirus distantly related to RTVL-H2 [54]. Like other ERV elements, the MER48 LTRs are prominent in the human genome with over 200 non-redundant insertions [41], overlapping nine different lncRNA genes (this study). With at least one MER48 repeat found inserted into an Alu-J element, the MER48 repeat can be dated to at most 80 million years before present [54,61], which is at least 10 million years after the estimated time of divergence of rodents and primates [62]. In apes and OWMs, the MER48 LTR and corresponding *EVADR* lncRNA are highly conserved. While MER48 elements are present in NWMs and prosimians we could not identify a MER48 insertion at the expected *EVADR* locus in these groups (Figure 7). These observations suggest an active MER48 element inserted at the *EVADR* locus sometime after the split between NWMs and OWMs, but before the split between OWM and apes. As the MER48 LTR provides a promoter and contributes sequence to the *EVADR* exons, it appears the apes and OWMs have co-opted a MER48 insertion to generate the lncRNA *EVADR* and this arrangement has been maintained evolutionarily*.*

Unlike most lncRNAs, which show modest conservation [5,63-65], the human *EVADR* lncRNA has remarkably high sequence identity among non-human primates, similar to the constrained exonic sequences of protein coding genes. Analysis of the *EVADR* consensus structure reveals the majority of the evolutionary structural base pair substitutions are compensatory, which strongly suggests the RNA secondary structure is critical for *EVADR* function (Figure 8). Further supporting strong purifying selection for *EVADR*, we find the splice junctions of both introns to be conserved in apes and OWMs, but not in NWMs or prosimians. These findings are consistent with previous reports describing conserved splice junctions for lncRNAs [66,67]. Many lncRNAs do not have high sequence or structural conservation and yet can have biological functions [64,68,69]. However, the high level of sequence conservation observed for *EVADR* is atypical; this suggests the biological function of *EVADR* may also be manifested through its RNA structural features rather than its primary sequence. Collectively, the constraint on the *EVADR* exons, splice junctions and promoter sequence, coupled with the restricted expression patterns, suggest this gene may play an important, yet undetermined role in primate biology.

The *EVADR* MER48 LTR not only contributes sequence to the 5′ exon of the lncRNA, but also a functional promoter that may direct aberrant expression of *EVADR* in cancer. We quantified *EVADR* expression across 25 different TCGA tumor types to find this lncRNA is strongly associated with colon, rectal, lung, stomach and pancreas adenocarcinomas, but not with corresponding normal tissues. In general, the eight other MER48-lncRNAs have low expression in these same tumors and do not correlate with *EVADR* tumor expression profiles. Thus, relative to other MER48-lncRNAs, there is specific activation of *EVADR* in these adenocarcinomas. There is a possibility that *EVADR* has a non-adenylated form which would not be detectable in TCGA datasets since they are derived from poly(A)+ RNA. Two other studies have found ERV LTR-mediated activation of gene expression in cancer but both of these studies described protein coding genes that employ an ERV LTR as an alternative promoter. The first study reported activation of the colony stimulating factor 1 receptor (*CSF1R*) gene in Hodgkin’s lymphoma [70] and the second reported activation of the fatty acid binding protein 7 (*FABP7*) gene in diffuse large B-cell lymphoma [71]. In cancer cell lines there are a number of examples of the recently described very long intergenic non-coding RNAs (vlincRNAs) displaying transcriptional activation mediated by a ERV [72]. While it is known that ERV LTRs can drive aberrant expression of protein-coding genes, our study expands this to show they can modulate the expression of lncRNAs in human cancer in a highly specific manner.

The adenocarcinoma-specific expression of *EVADR* suggests this lncRNA may have a distinct function in tissues and tumors of a glandular origin. Consistent with this observation, many lncRNAs have been reported to demonstrate tissue- or cell type-specific expression [7,8]. More recently, another class of ERV-associated lncRNAs, HERVH-lncRNAs, have been reported to be specifically active in embryonic stem cells, where they are associated with pluripotency [18,73,74]. However, HERV-H LTRs also demonstrate stem cell-specific expression [28], which may suggest the HERVH-lncRNAs are activated, at least in part, by virtue of their association with the HERV-LTR. We found no evidence for *EVADR* expression in stem cells in our analysis of ENCODE cell line RNA-seq data (Figure S3B in Additional file 1), but it is possible *EVADR* may be active in early developmental processes. In a manner similar to the HERVH-lncRNAs, the MER48 LTRs may provide a regulatory mechanism to drive tissue- or tumor-specific lncRNA expression.

## Conclusions

The conservation of *EVADR* in recent primate evolution and the MER48-mediated activation of *EVADR* in adenocarcinoma highlight the need for further studies to elucidate the normal function of *EVADR* and its relevance to cancer biology. Ongoing experiments will identify *EVADR*’s protein and RNA interacting partners, chromatin binding sites, and effects on gene expression, and *EVADR* knock-in mice will likely be useful for elucidating *EVADR*’s biological and oncogenic phenotypes. It is also possible that the activation of *EVADR* does not contribute to oncogenesis, but rather is a consequence of changes to the transcriptional regulatory environment typical of cancer cells. Regardless of a biological function, the specificity of *EVADR* activation in adenocarcinomas coupled with the poorer survival probability that tracks with elevated *EVADR* expression suggest that further characterization of *EVADR* as a candidate adenocarcinoma biomarker is warranted.

## Additional files

Additional file 1: Figures S1-S9. Supplementary figures. Additional file 2: Table S1. EVADR expression and PCR validation in the colorectal cancer dataset (SRP010181). Additional file 3:
**ClustalW alignments. This file contains the ClustalW alignments for the** ***EVADR*** **cDNA, introns, and MER48 LTR for 13 primates.**

## Abbreviations

ERV: endogenous retrovirus
EVADR: Endogenous retroViral-associated ADenocarcinoma RNA
FPKM: fragments per kilobase of exon per million fragments mapped
lncRNA: long non-coding RNA
LTR: long terminal repeat
NWM: New World monkey
ORF: open reading frame
OWM: Old World monkey
RACE: rapid amplification of cDNA ends
RLU: relative luciferase units
RPK: reads per kilobase
RPKMS: reads per kilobase per million sequenced
SD: standard deviation
