# Supplementary material for: Activation of an endogenous retrovirus-associated long non-coding RNA in human adenocarcinoma
Source: Genome Med. 2015 Mar 5;7(1):22. doi: 10.1186/s13073-015-0142-6 (PMC4375928; doi:10.1186/s13073-015-0142-6)
Supplement: Additional file 1: Figures S1-S9. — Supplementary figures. [file 13073_2015_142_MOESM1_ESM.pdf]

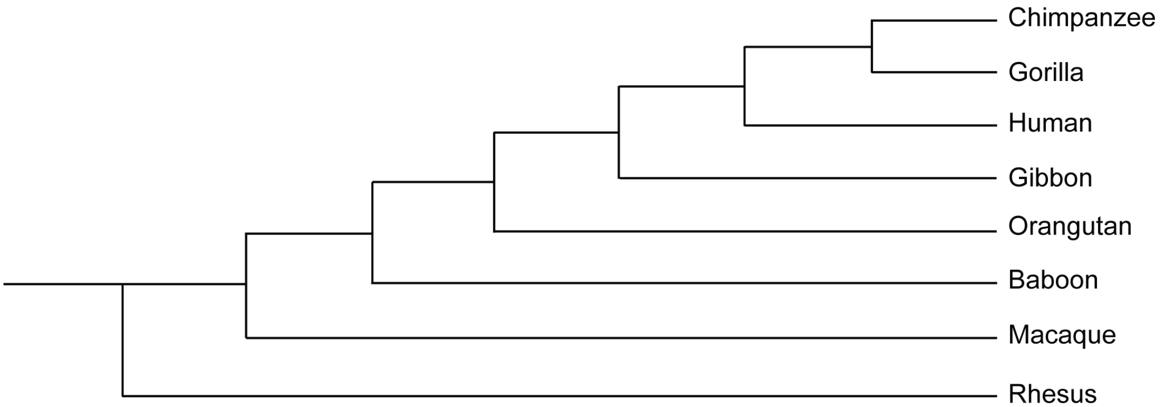

Figure S1: Maximum Parsimony analysis of primates based on EVADR cDNA alignments. The evolutionary history was inferred using the Maximum Parsimony method. The most parsimonious tree with length = 117 is shown. The consistency index is (0.73), the retention index is (0.80), and the composite index is 0.67 (0.58) for all sites and parsimony-informative sites (in parentheses). Evolutionary analyses were conducted in MEGA6 [42].

```
ORF Frame +1; 36aa (232-342)

  232 atgtcctggaaagtttgctgtcacatctccattatgacaaaagca
      M  S  W  K  V  C  C  H  I  S  I  M  T  K  A
  277 ttgtgccgaacagatgaaaaaatgcattgtcaacggaatctttta
      L  C  R  T  D  E  K  M  H  C  Q  R  N  L  L
  322 tgtttgtttgtcttccttttaa 342
      C  L  F  V  F  L  *
```

```
ORF Frame +2; 42aa (53-181)

   53 atgttgctccccactgcctcgtgttgtctgttggcgcgctgtcgg
      M  L  L  P  T  A  S  C  C  L  L  A  R  C  R
   98 ggttcgaaccgatacaagaaccttccacctacctggtgctttggc
      G  S  N  R  Y  K  N  L  P  P  T  W  C  F  G
  143 ctcatctataagcttttccactgtcctgaaacaagatag 181
      L  I  Y  K  L  F  H  C  P  E  T  R  *
```

```
ORF Frame +3; 96aa (3-293)

    3 tgccattttcagcctcagcacgcctgcacccaggcgctcattaaa
      C  H  F  Q  P  Q  H  A  C  T  Q  A  L  I  K
   48 acagcatgttgctccccactgcctcgtgttgtctgttggcgcgct
      T  A  C  C  S  P  L  P  R  V  V  C  W  R  A
   93 gtcggggttcgaaccgatacaagaaccttccacctacctggtgct
      V  G  V  R  T  D  T  R  T  F  H  L  P  G  A
  138 ttggcctcatctataagcttttccactgtcctgaaacaagataga
      L  A  S  S  I  S  F  S  T  V  L  K  Q  D  R
  183 gaatctgagcggccagtcatctgccctaagtgctgccgccgaaga
      E  S  E  R  P  V  I  C  P  K  C  C  R  R  R
  228 ctgaatgtcctggaaagtttgctgtcacatctccattatgacaaa
      L  N  V  L  E  S  L  L  S  H  L  H  Y  D  K
  273 agcattgtgccgaacagatga 293
      S  I  V  P  N  R  *
```

Figure S2: Predicted ORFs in the *EVADR* lncRNA. The ORFs were identified using the online tool ORF finder and the 394-nt *EVADR* cDNA sequence. Coordinates are relative to the initiating nucleotide of the *EVADR* RNA.

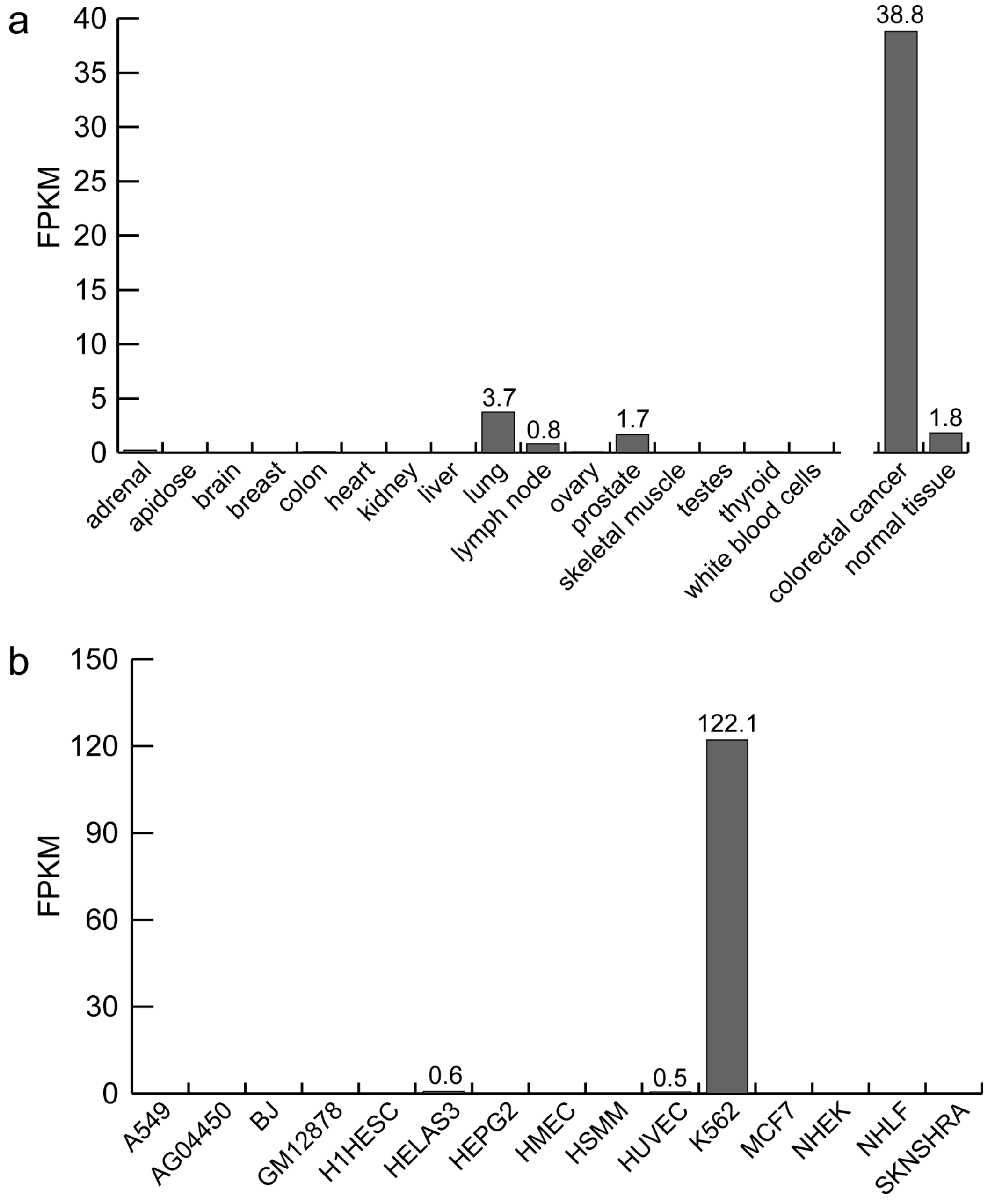

Figure S3: *EVADR* is rarely expressed in normal tissues or cell lines. (a) *EVADR* expression in Human bodymap 2.0. At the right, the mean expression (Tophat / Cufflinks) of *EVADR* from the colorectal tumor and matched normal discovery dataset (SRP010181) is shown for comparison. (b) *EVADR* expression in ENCODE cell lines. These data were analyzed using Cufflinks [36]. Expression values of 0.5 FPKM or greater are indicated.

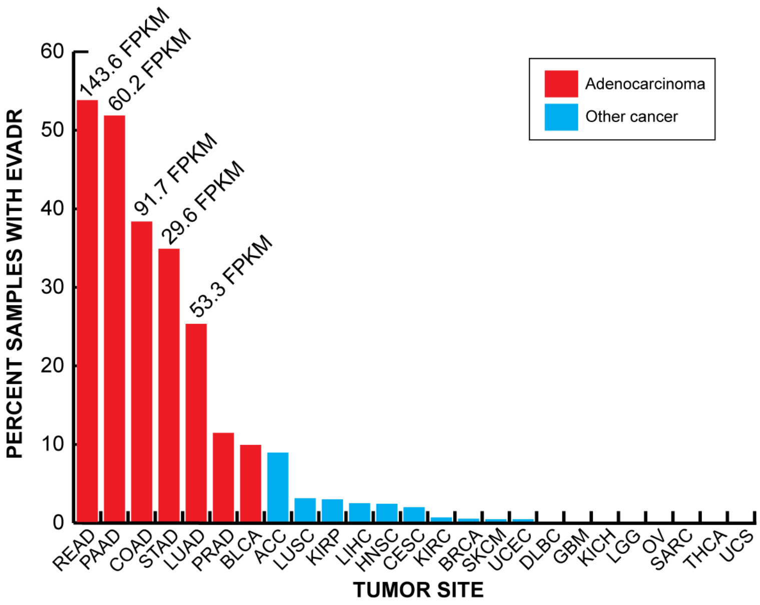

Figure S4: *EVADR* is highly expressed in adenocarcinomas. (a) Percent of TCGA tumors with detectable *EVADR* using the targeted read assembler TASR [37]. For tumor sites where full transcriptome assembly of the TCGA data was performed the FPKM values are indicated.

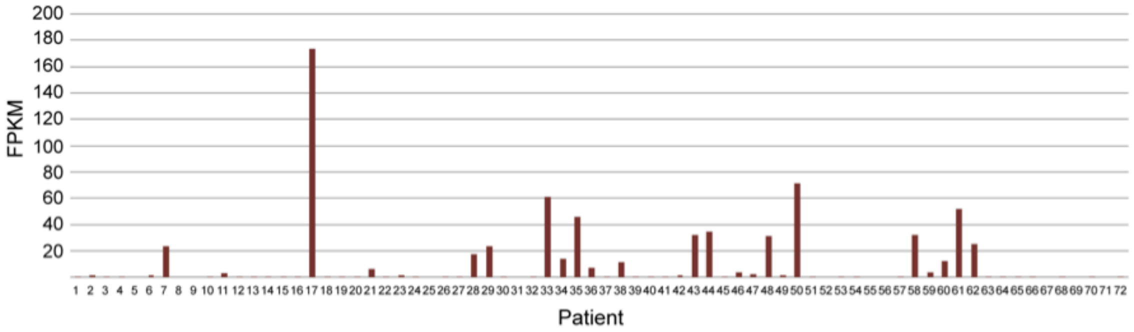

Figure S5: *EVADR* expression in tumors from 72 patients with lung adenocarcinoma (GSE40419). For each subject (x-axis) the tumor expression (mean 9.8 ± 24.7 FPKM (mean ± SD)) is shown in red. In normal lung tissue *EVADR* was generally found not to be expressed or expressed at low levels (0.44 ± 0.55 FPKM (mean ± SD)).

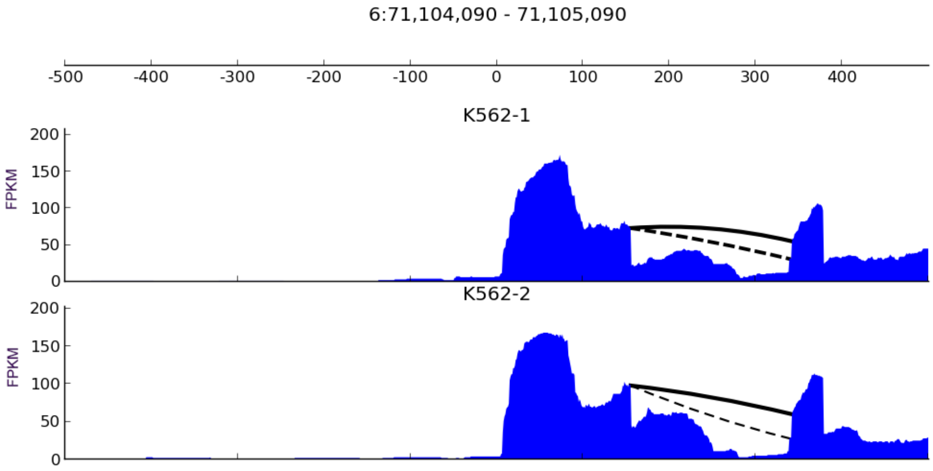

Figure S6: MER48-promoted *EVADR* transcription is unidirectional *in vivo* (K562 cells). Shown is a Sashimi [75] plot generated using the Integrative Genomics Viewer (IGV) and duplicate ENCODE K562 cell line datasets. The x-axis is the nucleotide count relative to the experimentally determined transcriptional start for *EVADR*, indicated by a 0. The lines indicate splice junction reads.

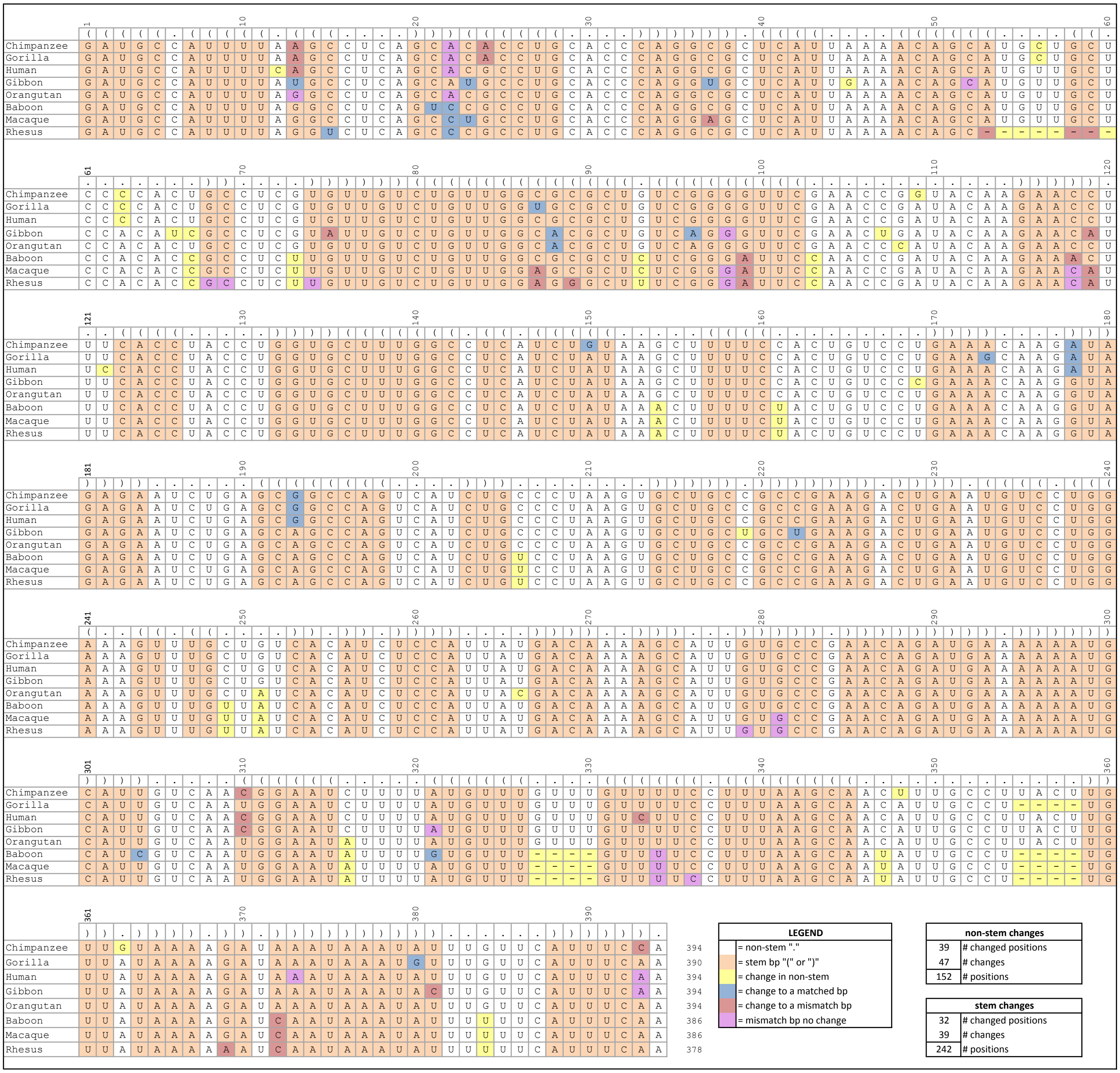

Figure S7: Sequence alignment for EVADR in eight OWM and apes. Orange indicates stem bp, white a non-stem nucleotide, yellow a change in a non-stem, blue a change to a matched bp, red a change to a mismatched bp and purple a mismatch bp with no change. The brackets "(" and ")" indicated paired nucleotides, the dots "." indicate unpaired nucleotides. The nucleotide position is indicated numerically at the top of each row.

position	1	303	13	22	14	21	15	20	23	38	24	37	52	75	53	74	58	69	59	68	87	281	88	280	89	279	96	229
	(	)	(	)	(	)	(	)	(	)	(	)	(	)	(	)	(	)	(	)	(	)	(	)	(	)	(	)
Chimpanzee	G	U	A	A	G	C	C	G	C	G	A	C	C	G	A	U	G	C	C	G	C	G	G	U	C	G	G	U
Gorilla	G	U	A	A	G	C	C	G	C	G	A	C	C	G	A	U	G	C	C	G	U	G	G	U	C	G	G	U
Human	G	U	A	A	G	C	C	G	C	G	G	C	C	G	A	U	G	C	C	G	C	G	G	U	C	G	G	U
Gibbon	G	U	U	A	G	C	C	G	U	G	G	U	C	A	A	U	G	C	C	G	C	G	A	U	C	G	A	U
Orangutan	G	U	G	A	G	C	C	G	C	G	G	C	C	G	A	U	G	C	C	G	C	G	A	U	C	G	A	U
Baboon	G	C	G	C	G	U	C	G	C	G	G	C	C	G	A	U	G	C	C	G	C	G	G	U	C	G	G	U
Macaque	G	U	G	C	G	C	C	G	U	G	G	A	C	G	A	U	G	C	C	G	A	G	G	U	C	G	G	U
Rhesus	G	U	G	C	G	C	U	G	C	G	G	C	C	G	-	U	-	C	-	G	A	G	G	U	G	G	G	U

position	98	119	99	118	140	222	150	180	151	179	157	173	193	216	310	393	321	381	322	380	333	373	334	372	336	369
	(	)	(	)	(	)	(	)	(	)	(	)	(	)	(	)	(	)	(	)	(	)	(	)	(	)
Chimpanzee	G	C	G	C	G	C	G	U	U	A	U	A	G	U	C	C	A	U	U	A	U	A	U	A	C	G
Gorilla	G	C	G	C	G	C	A	U	U	A	U	G	G	U	U	A	A	U	U	G	U	A	U	A	C	G
Human	G	C	G	C	G	C	A	U	U	A	U	A	G	U	C	A	A	U	U	A	C	A	U	A	C	G
Gibbon	G	A	G	C	G	U	A	U	U	G	U	A	A	U	C	A	A	C	U	A	U	A	U	A	C	G
Orangutan	G	C	G	C	G	C	A	U	U	G	U	A	A	U	U	A	A	U	U	A	U	A	U	A	C	G
Baboon	G	C	A	A	G	C	A	U	U	G	U	A	A	U	U	A	G	U	U	A	U	A	U	C	C	G
Macaque	G	A	A	C	G	C	A	U	U	G	U	A	A	U	U	A	A	U	U	A	U	A	U	C	C	G
Rhesus	G	A	A	C	G	C	A	U	U	G	U	A	A	U	U	A	A	U	U	A	U	A	U	C	C	A

LEGEND	
(blue)	= change to a matched bp
(red)	= change to a mismatch bp
(purple)	= mismatch bp no change

CHANGES	
32	# changed positions
39	# changes
19	# changed to a matched bp
20	# changed to a mismatch bp

Figure S8: Nucleotide changes in the stems of EVADR in OWM and apes. For the eight primates, colors are consistent with Supplementary Figure 6. Orange indicates stem bp, blue a change to a matched bp, red a change to a mismatched bp and purple a mismatch bp with no change. The indicated paired nucleotides, the dots "." indicate unpaired nucleotides. The paired nucleotides are indicated numerically at the top of each row and by brackets "(" and ")".

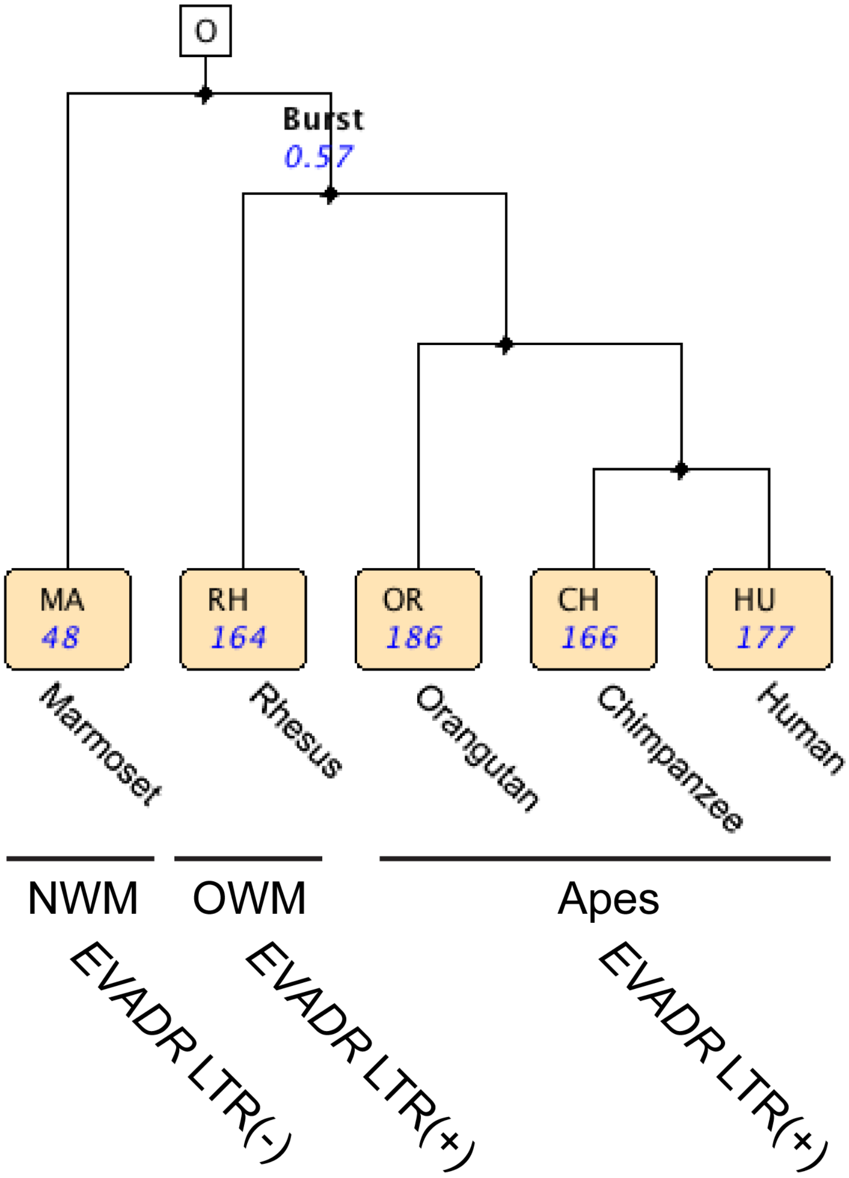

Figure S9: MER48 elements underwent a burst of insertion events after the split between NWM and OWM. Shown is a screen shot from the GEBRET (http://neobio.cs.pusan.ac.kr/~gebre/) [56] output for MER48 with the eFISH option and no filters applied. The number is the number of MER48 elements reported for each species.
