# Supplementary material for: Activation of an endogenous retrovirus-associated long non-coding RNA in human adenocarcinoma
Source: Genome Med. 2015 Mar 5;7(1):22. doi: 10.1186/s13073-015-0142-6 (PMC4375928; doi:10.1186/s13073-015-0142-6)
Supplement: Additional file 2: Table S1. — EVADR expression and PCR validation in the colorectal cancer dataset (SRP010181). [file 13073_2015_142_MOESM2_ESM.pdf]

Table S1: EVADR expression and PCR validation in the colorectal cancer dataset (SRP010181).

Patient	Normal FPKM	Normal PCR	EVADR FPKM	EVADR PCR
**1**	0	negative	0	negative
**2**	0	negative	0	negative
**3**	0	negative	19.8408	positive
**4**	0	negative	3.2411	positive
**5**	0	negative	0	negative
**6**	0	negative	0	negative
**7**	0	negative	0	negative
**8**	4.0475	positive	0	negative
**9**	0	negative	0	negative
**10**	0	negative	0	negative
**11**	0	negative	0	negative
**12**	0	negative	10.6298	positive
**13**	0	negative	0	negative
**14**	1.2541	negative	7.0695	positive
**15**	0	negative	0	negative
**16**	0	negative	0	negative
**17**	0	negative	0	negative
**18**	0	negative	9.9498	positive
**19**	0	negative	0	negative
**20**	0	negative	0	negative
**21**	0	negative	0	negative
**22**	0	negative	0	negative
**23**	1.8255	negative	75.6693	positive
**24**	0	negative	31.242	positive
**25**	0	negative	0	negative
**26**	0	negative	2.0184	positive
**27**	0	negative	0	negative
**28**	0	negative	2.8828	negative
**29**	0	negative	40.9878	positive
**30**	0	negative	41.1789	positive
**31**	0	negative	0	negative
**32**	0	negative	33.7404	positive
**33**	2.0162	no data	0	negative
**34**	0	negative	17.178	positive
**35**	0	negative	2.3271	weak positive
**36**	0	negative	92.2369	positive
**37**	0	negative	0	negative
**38**	0	negative	0	negative
**39**	0	negative	0	negative
**40**	0	negative	75.1172	positive
**41**	0	negative	0	negative
**42**	0	negative	10.8802	positive
**43**	0	negative	0	negative
**44**	0	negative	0	negative
**45**	0	negative	0	negative
**46**	0	negative	62.6437	positive
**47**	4.0243	negative	7.5601	positive
**48**	0	negative	0	negative
**49**	6.1227	positive	0	negative
**50**	0	negative	42.9171	positive
**51**	0	negative	15.0372	negative

**52**	0	negative	33.6155	positive
**53**	0	negative	30.7091	positive
**54**	0	negative	85.7923	positive
**55**	0	negative	0	negative
**56**	0	negative	5.229	weak positive
**57**	0	negative	80.0007	positive
**58**	0	negative	47.2241	positive
**59**	7.0481	positive	0	negative
**60**	0	negative	0	negative
**61**	5.9652	weak positive	0	negative
**62**	0	negative	33.2578	positive
**63**	0	negative	0	negative
**64**	0	negative	12.8129	negative
**65**	0	negative	0	positive
