# Supplementary material for: Activation of an endogenous retrovirus-associated long non-coding RNA in human adenocarcinoma
Source: Genome Med. 2015 Mar 5;7(1):22. doi: 10.1186/s13073-015-0142-6 (PMC4375928; doi:10.1186/s13073-015-0142-6)
Supplement: Additional file 3: — ClustalW alignments. This file contains the ClustalW alignments for the EVADR cDNA, introns, and MER48 LTR for 13 primates. [file 13073_2015_142_MOESM3_ESM.pdf]

Supplementary File 1: Full sequence alignments for EVADR locus.

CLUSTAL alignment for MER48 LTR at EVADR locus

Human MER48 LTR:
Position: chr6:71104329-71104716
Band: 6q13

```
Human           CTTACTGAAAGATTCTCCCC-GGGGCCTGAAAGTTTAAGGGAATGAATAACTCCGCCCTC
Chimpanzee      CTTACTGAAAGATTCTC-CC-GGAGCCTGAAAGCTTAAGGGAATGAATAACTCCGCCCTC
Gorilla         CTTACTGAAAGATACTCCCC-GGGGCCTGAAAGCTTAAGGGAATGAATAACTCCGCCCTC
Orangutan       CCTGCTGAAAGATTCTCACC-GGGGCCTGAAAGCTTAAGAGAATGAATAACTCCGCCCTC
Gibbon          CCTACTGAAAGATTCTCCCT-GGGGCCTGAAAGCTTAAGGGAATGAATAACTCCGCCCTC
Baboon          CCTACGGAAAGATTCTCCCCAGGGGCCTGAAAGCTTAAGGGAATGAGTAACTCCTCCCTC
Macaque         CCTACGGAAAGATTCTCCCCAGGG-CCTGAAAGCTTAAGGGAATGAATAACTCCTCCCTC
Rhesus          CCTACGGAAAGATTCTCCCCAGGG-CCTGAAAGCTTAAGGGAATGAATAACTCCTCCCTC
Marmoset        CTTG--------------------------------------------------------
Squirrel_monkey CTCA--------------------------------------------------------
Tarsier         CCCA--------------------------------------------------------
Bushbaby        CCCA--------------------------------------------------------
Mouse_lemur     CCCA--------------------------------------------------------

Human           CTCAGGCCCAGTCCCAAGGTGCAAGGCCACTTGTGCTAGCAGCATGCGTCAGCAAGATAG
Chimpanzee      CTCAGGCCCAGTCCCAAGGTGCAAGGCCACTTGTGCTAGCAGCATGCGTCAGCAAGATAG
Gorilla         CTCAGGCCCAGTCCCAAGGTGCAAGGCCACTTGTGCTAGCAGCATGCGTCAGCAAGATAG
Orangutan       CTCAGGCCCAGTCCCAAGGTGCAAGGCCACTTGTGCTAGCAGCATGCATCAGCAAGATAG
Gibbon          CTCAGGCCCAGTCCCAAGGTGCAAGGCCACTTGTGCTAGCAGCATGCGTCAGCAAGATAG
Baboon          CTCAGGCCCAGTCCTCAGGCACAAAGCCACATGCGCTAGCAGTGTGCATCAGCAAGATAG
Macaque         CTCAGGCCCAGTCCTCAGGCGCAAAGCCACATGCGCTAGCAGTGTGCATCAGCAAGATAG
Rhesus          CTCAGGCCCAGTCCTCAGGCGCAAAGCCACATGCGCTAGCAGTGTGCATTAGCAAGATAG
Marmoset        ------------------------------------------------------------
Squirrel_monkey ------------------------------------------------------------
Tarsier         ------------------------------------------------------------
Bushbaby        ------------------------------------------------------------
Mouse_lemur     ------------------------------------------------------------

Human           CAGAAGCAGGAAGAGAGCTGGCCGGAAGATATGTACCCTGTGAAGACCGAGAGAGAGGCC
Chimpanzee      CAGAAGCAGGAAGAGAGCTGGCCGGAACATATGTACCCTGTGAAGACCGAGAGAGAGGCC
Gorilla         CAGAAGCAGGAAGAGAGCTGGCCGGAAGATATGTACCCTGTGAAGACCGAGAGAGAGGCC
Orangutan       CAGAAGCAGGAAGAGAGCTGGCCGGAACATATATACCCTGTGAAGAC--AGAGAGAGGCC
Gibbon          CAGAAGCAGGAAGAGAGCTGGCCAGAAGATATGTACCATGTGAAGACCGAGAGAGAGGCC
Baboon          CAGAAGCAGGAAGAGAGTTAGCCCAAAGACATGTACCCTGTGAAGACCCAGAGAGAGGCC
Macaque         CAGAAGCAGGAAGAGAGTTAGCCCGAAGACATGTACCCTGTGAAGACCCAGAGAGAGGCG
Rhesus          CAGAAGCAGGAAGAGAGTTAGCCCGAAGACATGTACCCTGTGAAGACCCAGAGAGAGGCC
Marmoset        ------------------------------------------------------------
Squirrel_monkey ------------------------------------------------------------
Tarsier         ------------------------------------------------------------
Bushbaby        ------------------------------------------------------------
Mouse_lemur     ------------------------------------------------------------

Human           GTCCAGGTACCACGTAGCAGTTACATCAGACTGAGACACTTCCTGTTTACAGGAGACTAT
Chimpanzee      GTCCAGGTACCGCGTAGCAGTTACATCAGACTGAGACACTTCCTGTTTACAGGAGACTAT
Gorilla         GTCCAGGTACCGCGTAGCAGTTATATCAGACTGAGACACTTCGTGTTTACAGGAGACTAT
Orangutan       ATCCAGGTACCACGTAGCAGTTACATCAGACTGAGACACTTCCTGTTTACAGGAGACTAT
Gibbon          ATCCAGGTACCGCGTAGCAGTTACATCAGACTGAGGCACTTCCTGTTTACAGGAGACTAT
Baboon          GTCCAGGTACCACGTAGCAGTTACATAAGACTGAGACATTTCCTGTTTACAGGAGACTAT
Macaque         GTCCACGTACCACGTAGCAGTTACATCAGACTGAGACATTTCCTGTTTACAGGAGACTAT
Rhesus          GTCCAGGTACCACGTAGCAGTTACATCAGACTGAGACATTTCCTGTTTACAGGAGACTAT
Marmoset        ------------------------------------------------------------
Squirrel_monkey ------------------------------------------------------------
Tarsier         ------------------------------------------------------------
Bushbaby        ------------------------------------------------------------
Mouse_lemur     ------------------------------------------------------------

Human           AAAACCCCTGCCCCATCCTCATTTGGTGCTGATGCCATTTTCAGCCTCAGCACGCCTGCA
Chimpanzee      AAAACCCCTGCCCCATCCTCATTTGGTGCTGATGCCATTTTAAGCCTCAGCACACCTGCA
Gorilla         AAAACCCCTGCCCCATCCTCATTTGGTGCTGATGCCATTTTAAGCCTCAGCACACCTGCA
Orangutan       AAAACCTCTGCCCCATCCTCATTTGGTGCTGATGCCATTTTAGGCCTCAGCACGCCTGCA
Gibbon          AAAACCCCTGCCCCATCCTCATTTGGTGCTGATGCCATTTTATGCCTCAGCATGCCTGCA
Baboon          AAAACCTCTGCCCCGTCCTCATTTGGTGCTGATGCCATTTTAGGCCTCAGTCCGCCTGCA
Macaque         AAAACCTCTGCCCCGTCCTCATTTGGTGCTGATGCCATTTTAGGCCTCAGCCTGCCTGCA
```

```
Rhesus         AAAACCTCTGCCCCGTCCTCATTTGGTGCTGATGCCATTTTAGGTCTCAGCCCGCCTGCA
Marmoset       ------------------------------------------------------------
Squirrel_monkey ------------------------------------------------------------
Tarsier        ------------------------------------------------------------
Bushbaby       ------------------------------------------------------------
Mouse_lemur    ------------------------------------------------------------

Human          CCCAGGCGCTCATTAAAACAGCATGTTGCTCCCCACTGCCTCGTGTTGTCTGTTGGCGCG
Chimpanzee     CCCAGGCGCTCATTAAAACAGCATGCTGCTCCCCACTGCCTCGTGTTGTCTGTTGGCGCG
Gorilla        CCCAGGCGCTCATTAAAACAGCATGCTGCTCCCCACTGCCTCGTGTTGTCTGTTGGTGCG
Orangutan      CCCAGGCGCTCATTAAAACAGCATGTTGCTCCACACTGCCTCGTGTTGTCTGTTGGCACG
Gibbon         CCCAGGTGCTCATTGAAACAGCATGTTGCTCCACATCGCCTCGTATTGTCTGTTGGCACG
Baboon         CCCAGGCGCTCATTAAAACAGCATGTTGCTCCACACCGCCTCTTGTTGTCTGTTGGCGCG
Macaque        CCCAGGAGCTCATTAAAACAGCATGTTGCTCCACACCGCCTCTTGTTGTCTGTTGGAGCG
Rhesus         CCCAGGCGCTCATTAAAACAGC--------CCACACCGCCTCTTGTTGTCTGTTGGAGGG
Marmoset       ------------------------------------------------------------
Squirrel_monkey ------------------------------------------------------------
Tarsier        ------------------------------------------------------------
Bushbaby       ------------------------------------------------------------
Mouse_lemur    ------------------------------------------------------------

Human          CTGTCGGGGTTCGAACCGATACAAGAACCTTCCACCTACCTGGTGCTTTGGCCTCATCTA
Chimpanzee     CTGTCGGGGTTCGAACCGGTACAAGAACCTTTCACCTACCTGGTGCTTTGGCCTCATCTG
Gorilla        CTGTCGGGGTTCGAACCGATACAAGAACCTTTCACCTACCTGGTGCTTTGGCCTCATCTA
Orangutan      CTGTCAGGGTTCGAACCCATACAAGAACCTTTCACCTACCTGGTGCTTTGGCCTCATCTA
Gibbon         CTGTCAGGGTTCGAACTGATACAAGAACATTTCACCTACCTGGTGCTTTGGCCTCATCTA
Baboon         CTCTCGGGATTCCAACCGATACAAGAAACTTTCACCTACCTGGTGCTTTGGCCTCATCTA
Macaque        CTCTCGGGATTCCAACCGATACAAGAACATTTCACCTACCTGGTGCTTTGGCCTCATCTA
Rhesus         CTTTCGGGATTCCAACCGATACAAGAACATTTCACCTACCTGGTGCTTTGGCCTCATCTA
Marmoset       --------------------------------------CCTAGTGCTTCAGTCTCATCTA
Squirrel_monkey --------------------------------------CCTGGTGCTTCAGTCTCATCTA
Tarsier        --------------------------------------CTAGGCACTTTGGTTTCGTCTA
Bushbaby       --------------------------------------AATGGTGCTCTGATTTCTTCTG
Mouse_lemur    --------------------------------------CTTGGTGCTTCAGTTTCTTCTA

Human          TAAGGTAAAAGCAAAGCCATCTGTTGCTAAATATTGGAAACTCTGTGTAGCAGTGAAGAT
Chimpanzee     TAAGGTAAAAGCAAAGCCATCTGTTGCTAAATATTGGAAACTCTGTGTAGCAGTGAAGAT
Gorilla        TAAGGTAAAAGCAAAGCCATCTGTTGCTAAATATTGGAAACTCTGTGTAGCAGTGAAGAT
Orangutan      TAAGGTAAAAGCAAAGCCATCTGTTGCTAAATATTGGAAACTCTGTGTAGCAGTGAAGAT
Gibbon         TAAGGTAAAAGCAAAGCCATCTGTTGCTAAATAT-GGAAACTCTGTGTGGCAGTGAAGAT
Baboon         TAAAGTAAAAGCAAAGCCATCTGTTGTTAAATAT-GGAAACTCTGTGTAGCAGTGAAGAT
Macaque        TAAAGTAAAAGCAAAGCCATCTGTTGTTAAATAT-GGAAACTCTGTGTAGCAGTGAAGAT
Rhesus         TAAAGTAAAAGCAAAGCCATCTGTTGTTAAATAT-GGAAACTCTGTGTAGCAGTGAAGAT
Marmoset       TAACGTAAAAGCAAAGCCATCTGTGGCTACATAT-GGAAACTCTGTGTACCAGTGAAGAT
Squirrel_monkey TAAGGTAAAAGCAAAGCCATCTGTGGCTACCTAT-GGAAACTCTGTGTACAAGTGAAGAT
Tarsier        TATGGTAAAAGCAACACCATCTGCAGCTAAGTACGGGAAG-TCAGTGTGGCAGTGAGGAT
Bushbaby       TAACCTAAGAGCAAAGCCAACTGGGGCTAAATACAAGAAG-TCAGCATGGCAATGAGGAT
Mouse_lemur    TAAGGTACAAGAAAAGCCATCTGCAGCTAAGTAGGAGAAG-TCATCGTGGCAGGGAGGAT

Human          TAGAAGTAG----TTAATGATTGGAGGATGAC
Chimpanzee     TAGAAGTAG----TTAATGATAGAAGGATGAC
Gorilla        TAGAAGTAG----TTAATGATAGAAGGATGAC
Orangutan      TAGGAGTAG----TTAATGATAGAAGGATGAC
Gibbon         TAGGAGTAG----TTAATGATAGAAGGATGAC
Baboon         -AGGAGTAG----TTAATGATAGAAGGATGAC
Macaque        TAGGAGTAG----TTAATGATAGAAGGATGAC
Rhesus         TAGGAGTAG----TTAATGATAGAAGGATGAC
Marmoset       TAGGTA--------TAATGATAGAAGGATGAC
Squirrel_monkey TAGGAA--------TAATGATAGAAGGATGAC
Tarsier        CAGAAGTAG----TTAATAATAGGAGGATGGC
Bushbaby       CAGAAGTAGATAGTTAATGATAGGAAG
Mouse_lemur    CAGGAGTAG----CTAATGACAGGAGCCTGGC
```

CLUSTAL alignment for 394-nt EVADR cDNA

Human EVADR exon coords (chr6):
exon 1: 71,104,593-71,104,746
exon 2: 71,104,933-71,104,969
exon 3: 71,108,918-71,109,120

```
Human              GATGCCATTTTCAGCCTCAGCACGCCTGCACCCAGGCGCTCATTAAAACAGCATGTT
Chimpanzee         GATGCCATTTTAAGCCTCAGCACACCTGCACCCAGGCGCTCATTAAAACAGCATGCT
Gorilla            GATGCCATTTTAAGCCTCAGCACACCTGCACCCAGGCGCTCATTAAAACAGCATGCT
Orangutan          GATGCCATTTTAGGCCTCAGCACGCCTGCACCCAGGCGCTCATTAAAACAGCATGTT
Gibbon             GATGCCATTTTATGCCTCAGCATGCCTGCACCCAGGTGCTCATTGAAACAGCATGTT
Baboon             GATGCCATTTTAGGCCTCAGTCCGCCTGCACCCAGGCGCTCATTAAAACAGCATGTT
Macaque            GATGCCATTTTAGGCCTCAGCCTGCCTGCACCCAGGAGCTCATTAAAACAGCATGTT
Rhesus             GATGCCATTTTAGGTCTCAGCCCGCCTGCACCCAGGCGCTCATTAAAACAGCC----
Marmoset           ---------------------------------------------------------
Squirrel_monkey    ---------------------------------------------------------
Tarsier            ---------------------------------------------------------
Bushbaby           ---------------------------------------------------------
Lemur              ---------------------------------------------------------

Human           GCTCCCCACTGCCTCGTGTTGTCTGTTGGCGCGCTGTCGGGGTTCGAACCGATACAAGAA
Chimpanzee      GCTCCCCACTGCCTCGTGTTGTCTGTTGGCGCGCTGTCGGGGTTCGAACCGGTACAAGAA
Gorilla         GCTCCCCACTGCCTCGTGTTGTCTGTTGGTGCGCTGTCGGGGTTCGAACCGATACAAGAA
Orangutan       GCTCCACACTGCCTCGTGTTGTCTGTTGGCACGCTGTCAGGGTTCGAACCCATACAAGAA
Gibbon          GCTCCACATCGCCTCGTATTGTCTGTTGGCACGCTGTCAGGGTTCGAACTGATACAAGAA
Baboon          GCTCCACACCGCCTCTTGTTGTCTGTTGGCGCGCTCTCGGGATTCCAACCGATACAAGAA
Macaque         GCTCCACACCGCCTCTTGTTGTCTGTTGGAGCGCTCTCGGGATTCCAACCGATACAAGAA
Rhesus          ----CACACCGCCTCTTGTTGTCTGTTGGAGGGCTTTCGGGATTCCAACCGATACAAGAA
Marmoset        ------------------------------------------------------------
Squirrel_monkey ------------------------------------------------------------
Tarsier         ------------------------------------------------------------
Bushbaby        ------------------------------------------------------------
Lemur           ------------------------------------------------------------

Human           CCTTCCACCTACCTGGTGCTTTGGCCTCATCTATAAGC-TTTTCCACTGTCCTGAA----
Chimpanzee      CCTTTCACCTACCTGGTGCTTTGGCCTCATCTGTAAGC-TTTTCCACTGTCCTGAA----
Gorilla         CCTTTCACCTACCTGGTGCTTTGGCCTCATCTATAAGC-TTTTCCACTGTCCTGAA----
Orangutan       CCTTTCACCTACCTGGTGCTTTGGCCTCATCTATAAGC-TTTTCCACTGTCCTGAA----
Gibbon          CATTTCACCTACCTGGTGCTTTGGCCTCATCTATAAGC-TTTTCCACTGTCCCGAA----
Baboon          ACTTTCACCTACCTGGTGCTTTGGCCTCATCTATAAAC-TTTTCTACTGTCCTGAA----
Macaque         CATTTCACCTACCTGGTGCTTTGGCCTCATCTATAAAC-TTTTCTACTGTCCTGAA----
Rhesus          CATTTCACCTACCTGGTGCTTTGGCCTCATCTATAAAC-TTTTCTACTGTCCTGAA----
Marmoset        ----------GCCTAGTGCTTCAGTCTCATCTATAACC-TTTTCCACTGTCCTGAA----
Squirrel_monkey ----------ACCTGGTGCTTCAGTCTCATCTATAAGC-TTTTCCACTGTCCTGAA----
Tarsier         ----------ACTAGGCACTTTGGTTTCGTCTATATGCATTTTCCCGTGTCCTGAATGTA
Bushbaby        ----------AAATGGTGCTCTGATTTCTTCTGTAACC-TTTTCCAATGTCCTGAATGTA
Lemur           ----------ACTTGGTGCTTCAGTTTCTTCTATAAG--TTTACCAATGTCCTGAATGTA

Human           ---ACAAGATAG------------------AGAATCTGAGCGGCCAGTCATCTGCCCTAA
Chimpanzee      ---ACAAGATAG------------------AGAATCTGAGCGGCCAGTCATCTGCCCTAA
Gorilla         ---GCAAGATAG------------------AGAATCTGAGCGGCCAGTCATCTGCCCTAA
Orangutan       ---ACAAGGTAG------------------AGAATCTGAGCAGCCAGTCATCTGCCCTAA
Gibbon          ---ACAAGGTAG------------------AGAATCTGAGCAGCCAGTCATCTGCCCTAA
Baboon          ---ACAAGGTAG------------------AGAATCTGAGCAGCCAGTCATCTGTCCTAA
Macaque         ---ACAAGGTAG------------------AGAATCTGAGCAGCCAGTCATCTGTCCTAA
Rhesus          ---ACAAGGTAG------------------AGAATCTGAGCAGCCAGTCATCTGTCCTAA
Marmoset        ---ACAAGATAG------------------AGAATCTAAGCACCCAGTCATCTGCCCTAA
Squirrel_monkey ---ACAAGATAG------------------AGAATCTAAGCAGCCAGTCATCTGCCCTAA
Tarsier         GAAACAAGATGGTATATTGGCCTAGGATGGATAAATTGAGCAACCAGCAATCTTCCCTGA
Bushbaby        GAAACGAGATGGCAGATTGGCCCAGGATAAGGAATTTGAGCAGCCAGAGATCTGCCCTGA
Lemur           GAAACGAGATAGCAGATTGGCCCAGGATAGAGAATTTGAGCAACCAATAATCTGCCCTAA

Human           GTGCTGCCGCCGAAGACTGAATGTCCTGGAAAGTTTGCTGTCACATCTCCATTATGACAA
Chimpanzee      GTGCTGCCGCCGAAGACTGAATGTCCTGGAAAGTTTGCTGTCACATCTCCATTATGACAA
Gorilla         GTGCTGCCGCCGAAGACTGAATGTCCTGGAAAGTTTGCTGTCACATCTCCATTATGACAA
Orangutan       GTGCTGCCGCCGAAGACTGAATGTCCTGGAAAGTTTGCTATCACATCTCCATTACGACAA
Gibbon          GTGCTGCTGCTGAAGACTGAATGTCCTGGAAAGTTTGCTGTCACATCTCCATTATGACAA
Baboon          GTGCTGCCGCCGAAGACTGAATGTCCTGGAAAGTTTGTTATCACATCTCCATTATGACAA
Macaque         GTGCTGCCGCCGAAGACTGAATGTCCTGGAAAGTTTGTTATCACATCTCCATTATGACAA
Rhesus          GTGCTGCCGCCGAAGACTGAATGTCCTGGAAAGTTTGTTATCACATCTCCATTATGACAA
Marmoset        GTGCTGCCTCTGAAGACTGAATATCCTGGAATGTTTGCTATCACATCTCCATTATGACAA
Squirrel_monkey GTGCTGCCGCTGAAGACTGAATGTCCTGGAATGTTTGCTATCACAACTCCATTATGACAA
```

```
Tarsier        GTGTG-----TGAAGGCTGAATGCCCTAGAAAGTTTGCCTTCATATGTCC------ACAA
Bushbaby       GTGCCACGGCTGAAGGCTGAACGCCCTGGAGAGTTTGCC-TCACGTTCCCA-TACTACAG
Lemur          GTGCTACGGCTGAAAGCTGAATGCCCCGGAGAGTTTGCCATCCCATCGCCACTACTACAG

Human          AAGCATTGTGC---CGAACAG----------------ATGAAAAAATGCATTGTCAACGG
Chimpanzee     AAGCATTGTGC---CGAACAG----------------ATGAAAAAATGCATTGTCAACGG
Gorilla        AAGCATTGTGC---CGAACAG----------------ATGAAAAAATGCATTGTCAATGG
Orangutan      AAGCATTGTGC---CGAACAG----------------ATGAAAAAATGCATTGTCAATGG
Gibbon         AAGCATTGTGC---CGAACAG----------------ATGAAAAAATGCATTGTCAACGG
Baboon         AAGCATTGTGC---CGAACAG----------------ATGAAAAAATGCATCGTCAATGG
Macaque        AAGCATTGTGC---CGAACAG----------------ATGAAAAAATGCATTGTCAATGG
Rhesus         AAGCATTGTGC---CGAACAG----------------ATGAAAAAATGCATTGTCAATGG
Marmoset       AAGCATTGTGC---AGAACAG----------------ATGAAAAAATGCATTGTCAATGG
Squirrel_monkey AAACATTGTGC---AGTACAG----------------ATGAAAAAATGCATTGTCAATAG
Tarsier        AAGCATGGTGC---TTAAAG-----------------ATGAAACAA-ACATTGTCAATAA
Bushbaby       AAGCACTGCGC---CAAACAG---------------------------------CAACAG
Lemur          AAGCCCTTCTCTTTTAAACAGCATCGTCTTGTTTATTATAAAAGA--------TCAACAA

Human          AATCTTTTATGTTTGTTTGTCTTCCTTTAAGCAACATTGCCTTACTTGTTATAAAAGATA
Chimpanzee     AATCTTTTATGTTTGTTTGTTTTCCTTTAAGCAACTTTGCCTTACTTGTTGTAAAAGATA
Gorilla        AATCTTTTATGTTTGTTTGTTTTCCTTTAAGCAACATTGCCT----TGTTATAAAAGATA
Orangutan      AATATTTTATGTTTGTTTGTTTTCCTTTAAGCAACATTGCCTTACTTGTTATAAAAGATA
Gibbon         AATCTTTTATGTTTGTTTGTTTTCCTTTAAGCAACATTGCCTTACTTGTTATAAAAGATA
Baboon         AATATTTTGTGTTT----GTTTTCCTTTAAGCAATATTGCCT----TGTTATAAAAGATC
Macaque        AATATTTTATGTTT----GTTTTCCTTTAAGCAATATTGCCT----TGTTATAAAAGATC
Rhesus         AATATTTTATGTTT----GTTTTCCTTTAAGCAATATTGCCT----TGTTATAAAAAATC
Marmoset       AATCTTTTATATTT----GTTTTCCTTTAAGCAACATTGCCT----TGTTATAAAAGATC
Squirrel_monkey AATCTTTTACATTT----GTTTTCCTTTAAGCAACATTGCCTTGTTTGTTATAAAAGATC
Tarsier        AATC----ATTTTTGTTTGTTTTCTTTTAAACAACATCGTCTTGCTTGTTATGAAAGGTC
Bushbaby       AATCATTTTTGTTTACTTGTTCTCTTTTAAACAGCATTGCCCTATTTGCTACAAACGGTC
Lemur          --------ATATTTGCTCAT-TTCTTTTAAGCAGCATTGTCTTGTTTATTATAAAAGGTT

Human          AATAAATATTTGTTCATTTCAA
Chimpanzee     AATAAATATTTGTTCATTTCCA
Gorilla        AATAAATGTTTGTTCATTTCAA
Orangutan      AATAAATATTTGTTCATTTCAA
Gibbon         AATAAATACTTGTTCATTTCAA
Baboon         AATAAATATTTTTTCATTTCAA
Macaque        AATAAATATTTTTTCATTTCAA
Rhesus         AATAAATATTTTTTCATTTCAA
Marmoset       AATAAATATTTGTTCATTTCAA
Squirrel_monkey AATAAATATTTGTTGATTTCAA
Tarsier        AATACATATTTGTTCCTTTCCA
Bushbaby       AATAAATGTTTGTTTATTTCAG
Lemur          AATAAATATTTGTTCATTTCGA
```

CLUSTAL alignment for EVADR introns (i1 and i2 merged)

Human EVADR intron coords (chr6):
intron 1(i1): 71,104,747-71,104,932
intron 2(i2): 71,104,970-71,108,917

```
Human          GTAAAAGCAAAGCCATCTGTTGCTAAATATTGGAAACTCTGTGTAGCAGTGAAGATTAGA
Chimpanzee     GTAAAAGCAAAGCCATCTGTTGCTAAATATTGGAAACTCTGTGTAGCAGTGAAGATTAGA
Gorilla        GTAAAAGCAAAGCCATCTGTTGCTAAATATTGGAAACTCTGTGTAGCAGTGAAGATTAGA
Orangutan      GTAAAAGCAAAGCCATCTGTTGCTAAATATTGGAAACTCTGTGTAGCAGTGAAGATTAGG
Gibbon         GTAAAAGCAAAGCCATCTGTTGCTAAA-TATGGAAACTCTGTGTGGCAGTGAAGATTAGG
Baboon         GTAAAAGCAAAGCCATCTGTTGTTAA-ATATGGAAACTCTGTGTAGCAGTGAAGATA-GG
Macaque        GTAAAAGCAAAGCCATCTGTTGTTAA-ATATGGAAACTCTGTGTAGCAGTGAAGATTAGG
Rhesus         GTAAAAGCAAAGCCATCTGTTGTTAA-ATATGGAAACTCTGTGTAGCAGTGAAGATTAGG
Marmoset       GTAAAAGCAAAGCCATCTGTGGCTACA-TATGGAAACTCTGTGTACCAGTGAAGATTAGG
Squirrel_monkey GTAAAAGCAAAGCCATCTGTGGCTACC-TATGGAAACTCTGTGTACAAGTGAAGATTAGG
Tarsier        GTAAAAGCAACACCATCTGCAGCTAAG-TACGGGAAGTCAGTGTGGCAGTGAGGATCAGA
Bushbaby       CTAAGAGCAAAGCCAACTGGGGCTAAATACAAGA-AGTCAGCATGGCAATGAGGATCAGA
Lemur          GTACAAGAAAAGCCATCTGCAGCTAAG-TAGGAGAAGTCATCGTGGCAGGGAGGATCAGG

Human          AGT--------AGTTAATGATTGGAGGA-------TGACCCCT--CTCAAAAAA-----A
Chimpanzee     AGT--------AGTTAATGATAGAAGGA-------TGACCCCT--CTCAAAAAA------
Gorilla        AGT--------AGTTAATGATAGAAGGA-------TGACCCCT--CTCAAAAAA-----A
Orangutan      AGT--------AGTTAATGATAGAAGGA-------TGACCCCT--CTCAAAAAA---AAA
Gibbon         AGT--------AGTTAATGATAGAAGGA-------TGACCCCT--CTCAAAAAA---AAA
```

```
Baboon          AGT--------AGTTAATGATAGAAGGATGACCCCTCTCAAAAAAA-A--AAAA---AGA
Macaque         AGT--------AGTTAATGATAGAAGGATGACCCCTCTCAAAAAAAAA--AAAA---AGA
Rhesus          AGT--------AGTTAATGATAGAAGGATGACCCCTCTCAAAAAAAAAAAAAAA---AGA
Marmoset        TAT--------AATGATAGAAGGATGACCAGAGGTGACCCCTC--CCCCC-GAA---AAA
Squirrel_monkey AAT--------AATGATAGAAGGATGACCAGAGGTGACCCCTC--CCCCCCCAA---AAA
Tarsier         AGTAGTTAATAATAGGAGGATGGCTAGAGGTGTCCTCTCTCAAAAGAA---------AAA
Bushbaby        AGTAGATAGTTAATGATAGGAAGATAGCCAGAGGTGTTCCCCCCCTCCACCCCTGCCAAA
Lemur           AGTAGCTAATGACAGGAGCCTGGCTGAGGTGTCCCTCCCCCCAAAAAA---------ACA

Human           -A------------------AAAAAAGTTACATTGAGGGCCCCGCAGACATTGAATGAAA
Chimpanzee      -A------------------AAAAAAGTTACATTGAGGGCCCAGCAGACATTGAATGAAA
Gorilla         AA------------------AAAAAAGTTACATTGACGGCCCAGCAGACATTGAATGAAA
Orangutan       AA------------------AAAAAAGTTACATTGAGGGCCCAGCAGACATTGAATGAAA
Gibbon          AA------------------AAAAAAATTACATTGAGGGCCCAGCAGACATTGAATGAAA
Baboon          AA------------------AAAAAAGTTACATTGAGGGCCCAGCAGACATTGAATGAAA
Macaque         AA------------------AAAAAAGTTACATTGAGGGCCCAGCAGACATTGAATGAAA
Rhesus          AA------------------AAAAAAGTTACATTGAGGGCCCAGCAGACATTGAATGAAA
Marmoset        AT------------------TGTAATTACATATTGAGGGCCCAGCAGACATTGAATGAAG
Squirrel_monkey AT------------------TGTAATTACACATTGAGGGCCCAGCAGACATTGAATGAAG
Tarsier         A---------------GTTTTTTGAAACGATATTGAGGTACCAACAGACATTGAATGAAA
Bushbaby        ACAGAAATCAAACAAACAAAAAAACACCTTTTTTTAAAGCCATATAGAGAGCTC---ATA
Lemur           A---------------CTTTT-TTAAGCCACATAGAGTGCTCAGTAAACATTTAATGAAA

Human           AATA---------TGAACTCAAGTCAAAAAATTGTTTTCGCA-TTTTTTTCCAGCAGGTA
Chimpanzee      AATA---------TGAACTCAAGTCAAAAAATTGTTTTTGCA-TTTTTTTCCAGCAGGTA
Gorilla         AATA---------TGAACTCAAGTAAAAAAACTGTTTTTGCA-TTTTTTTCCAGCAGGTA
Orangutan       AATA---------TGAACTCAAGTCAAAAAATTGTTTTTGCA-TTTTTTTCCAGCAGGTA
Gibbon          GATA---------TGAACTCAAGTCAAAAAATTGTTTTTGCA-ATTTTTTCCAGCAGGTA
Baboon          AATA---------TGAACTCAAGTTAAAAAATTTT--TTGCAT-TTTTTTCCAGCAGGTA
Macaque         AATA---------TGAACTCAAGTTAAAAATTTTT--TTGCAT-TTTTTTCCAGCAGGTA
Rhesus          AATA---------CGAACTCAAGTTAAAAATTTTT--TTGCATTTTTTTTCCAGCAGGTA
Marmoset        ACTA---------TAAACTCAAGTCAAAAAATTTTTCTGCATTTTTTTTGCCAGCAGATA
Squirrel_monkey GCTA---------TAAACTCAAGTCAAAAAATTTTTCAGCATTTT--TTTCCAGCAGGCA
Tarsier         ACTATGAACTTGTAGTATTGCAAGTCAAAAATTGCTTTTGCATTTTTCTTTCAGCAAGCA
Bushbaby        AACA---------TTTAATGAAAAGT-AAGAACTGCTTTTTCTTTTTTCTCCAGCAAGTA
Lemur           TATGTGAACTGCTTTTGCACTTTTTCCCAGCAA-CTTT----ACCAATGTCCTGAAGGTA

Human           AGATATGATCTGAAAAATGTTTGACATGGCTGCTTTTGCCATAAAAACAAAGTAGAAAAT
Chimpanzee      AGATATGGTATGAAAAATGTTTGACATGGCTGCTTTTGCCATAAAATCAAAGTAGAAAAT
Gorilla         AGATATGGTATGAAAAATGTTTGACATGGCTGCTTTTGCCATAAAAACATAGTAGAAAAT
Orangutan       AGATATGGTATGAAAAATGTTTGACATGGCTGCTTTTGCCATAAAAACAAAGTAGAAAAT
Gibbon          AGATATGGTATGAAAAATGTTTGACATGGCTGCTTTTGCCATAAAAACAAAGTAGAAAAT
Baboon          AGATATGGCATGAAAAATGTTTGACATGGCTACTTTTGCCGTAAACAGAAAGTAGAAAAT
Macaque         AGATATGGCATGAAAAATGTTTGACATGGCTACTTTTGCCGTAAACAGAAAGTAGAAAAT
Rhesus          AGATATGGCATGAAAAATGTTTGACATGGCTACTTTTGCCGTAAACAGAAAGTAGAAAAT
Marmoset        AGATATGGCATGAAAAATGTTTAACATAGCTACTTTTGCCATAG-AACAAAGAAGAAAAT
Squirrel_monkey ATATATGGCATGAAAAATGTTTGACATGGCTACTTTTGCCATAG-AACAAAGAAGAAAAT
Tarsier         AGATATGGCATGAAAAAATTGTACCATGGCTACTGTTGCCTTAAAACA-GAGTAGAAAAT
Bushbaby        AGATGCGGCCTGAAAAATTTTTAACATGGCAATTTTTGCCATAACACA-AAGTAGAAAAC
Lemur           AGACAAGGCATGAAAAAATTTTAACATGACTGCTTTTGCCATAAAACA-AACTAGAAAAT

Human           AATTAAATGTAG-TCCATTTCTAGAGTTACCT-AAGTGTAGTGTTGGCTTATAAGACAGA
Chimpanzee      AATTAAATGTAG-TCCATTTCTAGAGTTACCTGAAGTGTAGTGTTGGCTTATAAGACAGA
Gorilla         AATTAAATGTAG-TCCATTTCTAGAGTTACCTGAAGTGTAGTGTTGGCTTATAAGACAGA
Orangutan       AATTAAATGTAG-TCCATTTCTAGAGTTACCTGAAGTGTAGTGTTGGCTTATAAGACAGA
Gibbon          AATTAAATGTAG-TCCATTTCTAGAGTTACCTAAAGTGTAGTGTTGGCTTATAAGACAGA
Baboon          AATTAAAAGTAGTTCCATTTCTAGCGTTACCTAAAGTGCAGTGTTGCCTTATAAGACAGA
Macaque         AATTAAAAGTAGTTCCACTTCTAGCGTTACCTAAAGTGCAGTGTTGCCTTATAAGACAGA
Rhesus          AATTAAAAGTAGTTCCATTTCTAGCGTTACCTAAAGTGCAGTGTTGCCTTATAAGACAGA
Marmoset        AATTAAATGTAA-TCCATTTCAAGAGTTACCACAAGTGTAGTGTTGGCTTATAAGACAGA
Squirrel_monkey AATTAAATGTAG-TCCATTTCAAGAGTTACCACAAGTGTAGTGTTGGCTTATAAGACGGA
Tarsier         AATTAAATGTAG-T---ATACAAGACTTATCTAAAGTGTAGTGCTGGCTTGTGAGACGGA
Bushbaby        AATTAAA----------TGCAGTACATTACCTAACGTGTAGCATTGGCTTATGAGACAGA
Lemur           AATTAAATGTAG-TCCATTTCAAGAGTCACCTAAAGTGTAGTGTTGGCTTATGAGACAGA

Human           ATACCTGGACCCTGCCTCATGTGTGCCTATTGTTTGCTGAAAGGATTTTAATCCTATAAT
Chimpanzee      ATACCTGGACCTTGACTCATGTGTGCCTATTGTTTGCTGAAAGGATTTTAATCCTATAAT
Gorilla         ATACCTGGACCTTGCCTCATGTGTGCCTATTGTTTGCTGAAAGGATTTTAATCCTATAAT
Orangutan       ATACCTGGACCCTGCCTCATGTGTGCCTATTGTTTGCTGAAAGGATTTTAATCCTATAAT
Gibbon          ATACCTGGACCCTGCCTCATGTGTGCCTATTGTTTGCTGAAAGGATTTTAATCCTATAAT
Baboon          ATACCTGGACCCTGCCTCATGTGTGCCTATTGTTTGCTGAACGGATTTTAATCCTATAAT
Macaque         ATACCTGGACCCTGCCTCATGTGTGCCTATTGTTTGCTGAACGGATTTTAATCCTATAAT
Rhesus          ATACCTGGACCCTGCCTCATGTGTGCCTATTGTTTGCTGAACGGATTTTAATCCTATAAT
```

```
Marmoset        ATACCTGGACCCTGTCTCATGTGTGCCTGTCGTTTGCTGAAAGTATTTTAATCCTATAAT
Squirrel_monkey ATACCTGGACCCTGTCTCATGTGTGCCTATTGTTTGCAGAAAGGATTTTAATCCTATAAT
Tarsier         ATACCTGGAACCTGCATCA--GATCTCTATTGTTTCCTGAAAGGATTTTAATCCTATAAT
Bushbaby        ATACCTGGACCCTGCCTAA--TGTCCCTATTGTTGGCTGAAAAGTTTTTAATCCAATAAT
Lemur           ATACCTGGACCCTGCCTAA--CGCCCCTATTGTTTGCTGAAAGGTTTTTAATCCTATAAT

Human           AAAATCTCTCACAACATGCCATGGAAAGTATTTCCTAGAATTCTTAAAAACTAATGAGAA
Chimpanzee      AAAATCTCTCACAACATGCCATGGAAAGTATTTCCTAGAATTCTTAAAAACTAATGAGAA
Gorilla         AAAATCTCTCACAACATGCCATGGAAAGTATTTCCTAGAATTCTTAAAAACTAATGAGAA
Orangutan       AAAATCTCTCACAACATGCCATGGAAAGTATTTCCTAGAATTCTTAAAAACTAATGAGAA
Gibbon          AAAATCTCTCACAACATGCCATGGAAAGTATTTCCTAGAATTCTTAAAAACTAATGAGAA
Baboon          AAAATCTCTCACAACATGCCATGGAAAGTATTTCCTAGAATTCTTAAAAACTAATGAGAA
Macaque         AAAATCTCTCACAACATGCCATGGAAAGTATTTCCTAGAATTCTTAAAAACTAATGAGAA
Rhesus          AAAATCTCTCACAACATGCCATGGAAAGTATTTCCTAGAATTCTTAAAAACTAATGAGAA
Marmoset        AAAATCTCTCAAAACGTGCCATGGAAAGTATTTCCTAGAATTCTTAAAAACTAGTAAGAA
Squirrel_monkey AAAATCTCTCAAAACGTGCCATGGAAAGGATTTCCTAGAATTCTTAAAAACTAATGAGAA
Tarsier         AAAATCTCTCACAGCATATGATGGAAAGTATTTCCTAGAATTTTTAAACACTAATGAGAA
Bushbaby        GAAATCTCTTACAGCATACTATGGAAAGTATTTCCTAGAATTTTTAAAAACTAATGAGAA
Lemur           AAAATCTCTCACAGCATACTATGGAAAGTATTTCCTAGAATTTTTTAAAACTAATGAGAA

Human           GGAAGCTTGAATATGCTGATTAACATGGAAGCTTGTTACTGGAAAAGAAAAGCAAAACTG
Chimpanzee      GGAAGCTTGAATATGCTGATTAACATGGAAGCTTGTTACTGGAAAAGAAAAGCAAAACTG
Gorilla         GGAAGCTTGCATATGCTGATTAACATGGAAGCTTGTTACTGGAAAAGAAAAGCAAAACTG
Orangutan       GGAAGATTGAATATGCTCATTAACATGGAAGCTAGTTACTGGAAAAGAAAAGCAAAACTG
Gibbon          GGAAGCTTGAATATGCTGATTAACATGGAAGCTAGTTACTGGAAAAGAAAAGCAAAACTG
Baboon          GGAAGCTTGAACATGCTGATTAACATGGAAGCTAGCTACTGGAAAAGAAAAGCAAAATTG
Macaque         GGAAGCTTGAACATGCTGATTAACATAGAAGCTAGCTACTGGAAAAGAAAAGCAAAATTG
Rhesus          GGAAGCTTGAACATGCTGATTAACATAGAAGCTAGCTACTGGAAAAGAAAAGCAAAATTG
Marmoset        GGAAGCTTGAATATGCTGATTAACAGAGAAGCTAGTTACTGGAAAAGAAAAGCAAGGTTG
Squirrel_monkey GGAAGCTTGAATATGCTGATTAAAACAGAAGCTAGTTACTGGAAAAGAAAAGCAAGGTTG
Tarsier         GGAAACTTGAATATGCTGATTAACATCTAAGTTAGTTATTGGAAAAGAAAAGCAAGGTTG
Bushbaby        GGAAGCTTGAATATGCTGATTAATGTGCAAGCGAGTTATTGGACAAGAAAAGCAAGGTTG
Lemur           GGAAGCTTGAATATGCTGATTAACGTGTAAGCTAGCTACTGGAAAAGAAAAGCAAGGTTG

Human           AAAGATATTGTTTTAATATCCT------TTCAGCTACTCTAAAAGATTACATAACTGCAC
Chimpanzee      AAAGATATTGTTTTAATATCCT------TTCAGCTACTCTAAAAGATTACATAACTGCAT
Gorilla         AAAGATATTGTTTTAATATCCT------TTCAGCTACTCTAAAAGATTACATAACTGCAT
Orangutan       AAAGATATTGTTTTAATATCCT------TTCAGCTACTCTAAAAGATTGCATAACTGAAT
Gibbon          AAAGATATTGTTTTAATATCCT------TTCAGCTACTCTAAAAGATTACCTAACTGAAT
Baboon          AAAGATATTGTTTTAATATCCT------TTCAGCTACTCTAA--GATTACATAACTGAAT
Macaque         AAAGATATTGTTTTAATATCCT------TTCAGCTACTCTAA--GATTACATAACTGAAT
Rhesus          AAAGATATTGTTTTAATATCCT------TTCAGCTACTCTAA--GATTACATAACTGAAT
Marmoset        AAAGATATTG-TTTAATATCCT------TTCAGCTACTCTAAAAGATTATATAACTGAAT
Squirrel_monkey AAAGATATTG-TTTAATATCCT------TTCAGCTACTCTAAAAGATTATTTAACTGAAT
Tarsier         AAAGATTATTGTTTTAATATCC-----TTTCAGCTACTCAAAAAGATTACATAACTGACT
Bushbaby        AAAGATCATTGTTTTAATATCCTTTCTTTCAGCTACTCTGAAAAGATTACATAACTGATT
Lemur           AAAGATCATTGTTTTAATATCC-----TTTCAGCTACTCTAAAAGATTACATATCTGATT

Human           ATAGAACAAGCTATATAAAGTACAAGGCTTAGTCAAAGAAACATCAGGCTAATACCTGAG
Chimpanzee      ATAGAACAAGCTATATAAAGTACAAGGCTTAGTCAAAGAAACATCAGGCTAATACCTGAG
Gorilla         ATAGAACAAGCTATATAAAGTACAAGGCTTAGTCAAAGAAACATCAGGCTAATACCTGAG
Orangutan       ATAGAACAAGCTATATAAAGTACAAGGCTTAGTCAAAGAAACATCAGGCTAATACCTGAG
Gibbon          ATAGAACAAGCTATATAAAGTACAAGGCTGAGTCAAAGAAACATCAGGCTAATACCTGAG
Baboon          ATAGAACAAGCTATATAAAGTACAAGGCTTAGTAAAAGAAACATCAGGCTAATACCTGAG
Macaque         ATAGAACAAGCTATATAAAGTACAAGGCTTAGTAAAAGAAACATCAGGCTAATACCTGAG
Rhesus          ATAGAACAAGCTATATAAAGTACAAGGCTTAGTAAAAGAAACATCAGGCTAATACCTGAG
Marmoset        ATAGAACAAGCTATGTAAAGTACAAGGGTTAGTCAAAGAAACATCAGGCTAATACCTGAG
Squirrel_monkey ATAGAACAAGCTATAGTAAGTACAAGAGTTAGTTAAAGAAATATCAGGCTAATACCTGAG
Tarsier         ATGAAACAAGCTATATAAAGCACAAGGCTTAGTCAAAGAAATATCAGTCTAATACCTGAG
Bushbaby        ATAGAACAAGTTCTATAAAGAACAAGGCTTAGTCAAAGAAATATCAT-CTCATACCTGAG
Lemur           ATAGAACAAGTTATATAAAGTACAAGGGTTAGTCAAAGAAATATCAGTCTCATACCTGTA

Human           TTTTCATACTTAATACAAAAGTCTAAAATATTTCTTAC--CCCCGACCTGAATATGGCTT
Chimpanzee      TTTTCATACTTAATACAAAAGTCTAAAATATTTCTTAC--CCCCGACCTGAATATGGCTT
Gorilla         TTTTCATACTTAATACAAAAGTCTAAAATATTTCTTAC--CCCCGGCCTGAATATGGCTT
Orangutan       TTTTCATACTTAGTACAAAAGTCTAAAATATTTCTTAC--CCCCGACCCGAATATGGCTT
Gibbon          TTTTCATACTTAGAACAAAAGTCTAAAATATTTCTTAC--TCCCGATCTGAATATGGCTT
Baboon          TTTTCATACT-----------------------------------------TATGGCTT
Macaque         TTTTCATACT-----------------------------------------TATGGCTT
Rhesus          TTTTCATACT-----------------------------------------TATGGCTT
Marmoset        TTTTCATACTTAGTACGAAAGTCTAAAATATTTT-TGC--CCCCAACCTGAATATGGCTT
Squirrel_monkey TTTTCATACATAGTACAAAAGTCTAAAGTATTTTTTAC--CCCCAACCTGAATATGGCTT
Tarsier         TTTTCAGACTTAATATAAAAGTCTTAAAGATTTTTTTATCCCCTTCTTGAATATCACTT
```

```
Bushbaby        TTTTCCTACTTAGTATAAATGTCTAGATTTTTTTA-C--CCTCCAACTTTATATGGCTT
Lemur           TTTTCATACTTAGTATAAAAGTCTAGGAGACTTTATCCCCCACCTAAATAGTCTTAAAAT

Human           CAACTTCTCATACAATGTACTACTAGCCTAATAGATATTTTAGAATTAATTATTGCAAGT
Chimpanzee      CAACTTCTCATACAATGTACTACTAGCCTAATAGACATTTTAGAATTAATCATTGCAAGT
Gorilla         CAACTTCTCATACAATGTACTACTAGCCTAATAGACATTTTAGAATTAATTATTGCAAGT
Orangutan       AAACTTCTCATACAATGTCCTACTAGCCTAATAGACATTTTAGAATTAATTATTGCAAGT
Gibbon          AAAATTCTCATACAATGTACTACTAGCCTAATAGACATTTTAGAATTAATTATTGCAAGT
Baboon          AAACTTCTCATACAATGTACTACTAGCCTAATAGACATTTTAGAATTAATTATTGCAAGT
Macaque         AAACTTCTCATACAATGTACTACTAGCCTAATAGACATTTTAGAATTAATTATTGCAAGT
Rhesus          AAACTTCTCATACAATGTACTANNNNNNNNNNNNNNNNNNNNNNNNNNNNNNNN------
Marmoset        AAACTTTTCATACAATGTACTACTGACCTCATAGACATTTTAGAATTAATTATTGCAAGT
Squirrel_monkey AAACTTTTCATACAATGTTCTACTGGCCTAATAGACATTTTAGAATTAATTATTGCAAGT
Tarsier         AAACCTCTCATACAATGCACTACTGGCCTAATAGACATTTTATAATTAATCATTACAAGT
Bushbaby        GAAATTCCCATACAATGCTCTACTAGCAA---AGACATTTTAGAACTAATTGTTACAAGC
Lemur           TCCCATACA------TGCTTTACT--ACATATAGACATTCTAGA----------------

Human           AGTTCATGTATATAAATGCATTTTTATTCAACTTTAA-----------------------
Chimpanzee      AGTTCATGTATATAAATGCATTTTTATTCAACTTTAA-----------------------
Gorilla         AGTTCATGTATATAAATGCATTTTTATTCAACTTTAA-----------------------
Orangutan       AGTTCATGTAGATAAATGCATTTTTATTCAACTTGAA-----------------------
Gibbon          AGTTCATGTAGATAAATGCATTTTTATTCAACTTTAA-----------------------
Baboon          AGTTCATGGAGATAAGTGCATTTTTATTCAACTTTAA-----------------------
Macaque         AGTTCATGGAGATAAGTGCATTTTTATTCAACTTTAA-----------------------
Rhesus          ------------------------------------------------------------
Marmoset        AGTTCATGTAGATAAATGCATTTTTATTTAACTTTAA-----------------------
Squirrel_monkey AGTTCATGTAGATAAATGCATTTTTATTTAACTTTAA-----------------------
Tarsier         AGTTTGTGTATCTAAATGCATTT-------------------------TTGTTCAACTTT
Bushbaby        AGTTTGGGTAGATAAATGAATTTTTTTTATTTTTTGATAAATGAGTTTTGTTCAACTTT
Lemur           ------------------------------------------------------------

Human           --------GTAGT--TTGTTTCATTGTTTTGAGTATAAATAAGCAATCTCATCAATGATC
Chimpanzee      --------GTAGT--TTGTTTCATTGTTTTGAGTATAAATAAGCAATCTCATCAATGATC
Gorilla         --------GTAGT--TTGTTTCATTGTTTTGAGTATAAATAAGCAATCTCATCAATGATC
Orangutan       --------GTAGT--TTGTTTCACTGTTTTGAGTATAAATAAGCAATCTCATCAATGATC
Gibbon          --------GTAGT--TTGTTTCATTGTTTTGAGTATAAATAAGCAATCTCATCAATGATC
Baboon          --------GTAGT--CTGTTTCATTGTTTTGAGTATAAATAAGCAATCTCATCAATGATC
Macaque         --------GTAGT--TTGTTTCAT-----TGAGTATAAATAAGCAATCTCATCAATGATC
Rhesus          ----------------------------------------------------------NN
Marmoset        --------GTAGT--TTGTTTCATTGTTTTGAGTATAAATAAGCAATCTCATCAATGACC
Squirrel_monkey --------GTAGT--TTGTTTCATTGTTTTGAGTATAAATAAGCAATCTCATCAATGACC
Tarsier         GACATTAAGTTGTTTGTTGTAAGTTGTTTTGAGTGCAACTAGGCAATCTCTTCAATGATC
Bushbaby        GACATTAAGTAGTTTGTTGGAAGTTGTTTTGAGTATATTTAGGCAATCTCTTCAATGATC
Lemur           ------------------------------------------------------------

Human           ACTTCAAAGATACAGTTTATTAAATATCTTAATCATTATTCACAAAGCATTATAGGATTA
Chimpanzee      ACTTCAAAGATACAGTTTATTAAATATCTTAATCATTATTCACAAAGCATTATAGGATTA
Gorilla         ACTTCAAAGATACAGTTTATTAAATATCTTAATCATTATTCACAAAGCATTATAGGATTA
Orangutan       ACTTCAAAGATACAGTTTATTAAATATCTTAATCATTATTCACAAAGCATTATAGGATTA
Gibbon          ACTTCAAAGATACAGTTGATTAAATATCTTAATCATTATTCACAAAGCATTATAGGATTA
Baboon          ACTTCAAAGATACAGTTTATTAAATATCTTAATCATTATTCACAAAGCATTATAGGATTA
Macaque         ACTTCAAAGATACAGTTTATTAAATATCTTAATCATTATTCACAAAGCATTATAGGATTA
Rhesus          NNNNNNNNNNNNNNNNNNNNNNNNNNNCTTAATCATTATTCACAAAGCATTATAGGATTA
Marmoset        ATTTGAAAGATACAGTTTATTAAATATCTTAATCATTATTCATAAAGTATTATAGAATTA
Squirrel_monkey ATTTCAAAGATACAGTTTATTAAATATCTTAATCATTATTCATAAAGCATTATAGGATTA
Tarsier         ATTTCAAAGATATGCTTTATTAAATATTTTAATCATTATTCACAAAACAACATTGTAAGG
Bushbaby        ATTTCACAGATATAATTAGTTAAATATCTTAATAATTATTCACAAAGCAATACTATACGA
Lemur           ------------------------------------------------------------

Human           TTAGTA---AAGCTCCCTAATAGATAGTATTATTATTTTCACTCTTTCCAAGGAGTCAGT
Chimpanzee      TTAGTA---AAGCTCCCTAATAGATAGTATTATTATTTTCACTCTTTCCAAGGAGTCAGT
Gorilla         TTAGTA---AAGCTCCCTAATAGATAGTATTATTATTTTCACTCTTTCCAAGGAGTCAGT
Orangutan       TTAGTA---AAGCTCCCTAATAGATAGTATTATTATTTTCACTCTTTCCAAGGAGTCAGT
Gibbon          TTAGTA---AAGCTCCCTAATAGATAGTATATTTATTTTCACTCTTTCCAAGGAGTCAGT
Baboon          TTAGTA---AAGCTCCCTAATAGATAGTATTATTATTTTCACTCTTTCCAAGGAGTCAGT
Macaque         TTAGTA---AAGCTCTCTAATAGATAGTATCATTATTTTCACTCTTTCCAAGGAGTCAGT
Rhesus          TTAGTA---AAGCTCCCTAATAGATAGTATTATTATTTTCACTCTTTC-CAGGAGTCAGT
Marmoset        TCAGTA---AAGTTCCCTAATAGATAGTACTATTATTTTCACTCTTTCCAAGGAGTCAGT
Squirrel_monkey TCAGCA---AAGTTCCCTAATAGATAGTACTATTATTTTCACTCTTTCCAAGGAGTCAGT
Tarsier         TTAATAGTAAAGCTCCCTAATAGAGCGTATCATTACTTTCACTCTTTCCAAGCAGTCAGC
Bushbaby        TTAAAGTAAAGCTCCATAATAGA-----ATCACTACTTTACCCTTTCCAAGGAGTCAGT
Lemur           ------------------------------------------------------------
```

```
Human           TGTCAATATAAAAAAATACGTTTAGAGAATGGGTCAGTAAAGCAGCAATTTGAAT---AA
Chimpanzee      TGTTAATATTAAAAAATATGTTTAGAGAATGGGTCAGTAAAGCAGCAATTTGAAT---AA
Gorilla         TGTCAATATAAAAAAATATGTTTAGAGAATGGGTCAGTAAAGCAGCAATTTGAAT---AA
Orangutan       TGTCAATATAAAAAAATATGTTTAGAGAATGGGTCAGTAAAGCAGCGATTTGAAT---AA
Gibbon          TGTCAATATAAAAA-ATATGTTTAGAGAATGGGTCAGTAAAGCAGCGATTTGAAT---AA
Baboon          TGTCAATATAAAAA-ATATGTTTAGAGAATGGGTCAGTAAAGTAGCGATTTGAAT---AA
Macaque         TGTCAATATAAAAA-ATATGTTTAGAGAACGGGTCAGTAAAGCAGCGATTTGAAT---AA
Rhesus          TGTCAATATAAAAA-ATATGTTTAGAGAACGGGTCAGTAAAGCAGCGATTTGAAT---AA
Marmoset        CATCAATATAAAAA-ATATGTTTAGAGAATGGGTCAGTAAAGCAACAATTTGAAGGATAA
Squirrel_monkey TGTCAATATAAAAA-GTATGTTCAGAGAATGGGTCAGTAAAGCAGCAGTTTGAAGGATAA
Tarsier         TGTCAGTATAAGAAAATATGTTTAGGACATTTGTCAGTGAAGGAGGGACAAGACAAT---
Bushbaby        TGTCGACATAAACAATATGTTTAGGAAA-GGGGTT-------------------------
Lemur           ------------------------------------------------------------

Human           TGAAGGATATTTGAACGATTGAAGGATAATGAATCAAACTGAGGCAAACAAAGGAAGTGA
Chimpanzee      TGAAGGATATTTGAACGATTGAAGGATAATGAATCAAACTGAGGCAAACAAAGGAAGCGA
Gorilla         TGAAGGATATTTGAACGATTGAAGGATAATGAATCAAACTGAGGCAAACAAAGGAAGTGA
Orangutan       TGAAGGATATTTGAATGATTGAAGGATAATGAATCAAACTGAGGCAAACAAAGGAAGTGA
Gibbon          TGAAGGATATTTGAACGACTGAAGGATAATGAATCAAACTGAGGCAAACAAAGGAAGTGA
Baboon          TGAAGGATATTTGAACGATTGAAGGATAATGAATCAAACTGAGGCAAACAAAGGAAGTGA
Macaque         TGAAGGATATTTGAACAATTGAAGGATAATGAATCAAACTGAGGCAAACAAAGGAAGTGA
Rhesus          TGAAGGATATTTGAACAATTGAAGGATAATGAATCAAACTGAGGCAAACAAAGGAAGTGA
Marmoset        TGAAGAATATTTGAATGACTGAAGGATAATGAATCAAACTGAGGCAAACAAAGGAAGTGA
Squirrel_monkey TGAAGAATATTTGAATGACTGAAGGATAATGAATCAAACTGAGGCAAAC----GAAGTGA
Tarsier         -------------------TTATTGAAGAATAATTGAATGGAGGTAAACAGAGGAAATGA
Bushbaby        ---GGTGAAGACGACTTATTGAAGGATAAAGACTCAAATCGAGGCCAACACAGGAAGTGA
Lemur           ------------------------------------------------------------

Human           GAATGAAGAATAAAGTGATGTAAAGAATTAATATAAGAATAGTAAGGAGCATTTTATAAA
Chimpanzee      GAATGAAGAATAAAGTGATGTAAAGAATTAATATAAGAATAGTAAGGAGCATTTTATAAA
Gorilla         GAATGAAGAATAAAGTGATGTAAAGAATTAATATAAGAATAGTAAGGAGCATTTTATAAA
Orangutan       GAATGAAGAATAAAGTGATGTAAAGAATTAATATAAGAATAGTAAGGAGCATTTTATAAA
Gibbon          GAATGAAGAATAAAGTGATGTAAAGAATTAATATAAGAATAGTAAGGAGCATTTTATAAA
Baboon          AAATGAAGAATAAAGTGATGTAAAGAATTACTGTAAGAATAGTAAGGAGCATTTTATAAA
Macaque         AAATGAAGAATAAAGTGATGTAAAGAATTACTGTAAGAATAGTAAGGAGTATTTTATAAA
Rhesus          AAATGAAGAATAAAGTGATGTAAAGAATTACTGTAAGAATAGTAAGGAGTATTTTATAAA
Marmoset        GAATGAA--ATAAAGTAATGTCAATAATTAATATAAGAATAGTAAAGAGCATTTTATAAA
Squirrel_monkey GAATGAA--ATAAAGTAATGTCAATAATTAATATAAGAATAGTAAGGAGCATTTTATAAA
Tarsier         GAACGAAGAACAAAGCAATGTCAAGAATCAATGTAAGAACAATCAAGTGTGTTTTATAAA
Bushbaby        GAACGAAGAATCA--AGGTATAAAGAATTAATATAGTAATGGTGAGGTGCATTTTATAAA
Lemur           ------------------------------------------------------------

Human           TAAACCGTTAAAAGCAAGTTCTTCACAATAAACAAACTCTGAACAATGAGTCTATGAGGA
Chimpanzee      TAAACTGTTAAAAGCAAGTTCTTCACAATAAACAAACTCTGAACAATGAGTCTATGAGGA
Gorilla         TAAACTGTTAAAAGCAAGTTCTTCACAATAAACAAACTCTGAACAAAGAGTCTATGAGGA
Orangutan       TAAACTGTAAAAAGCAAATTCTTCACAATAAACAAACTCTGAACAATGAGTCTATGAGGA
Gibbon          TAAACTGTTAAAAGCAAGTTCTTCACAATAAACAAACTCTGAACAATGAGTCTATGAGGA
Baboon          CAAACTGTCAAAAGCAAATTCTTCACAATAAACAAACTCTGAACAATAAGTCTATGAGGA
Macaque         CAAACTGTCAAAAGCAAATTCTTCACAATAAACAAACTCTGAACAATAAGTCTATGAGGA
Rhesus          CAAACTGTCAAAAGCAAATTCTTCACAATAAACAAACTCTGAACAATAAGTCTACGAGGA
Marmoset        TAAACTGTTAAAAGTAAACTGTTCACAATAAACAAACTCTGAACAATGAGTCTGTGAGGA
Squirrel_monkey TAAACTGTTAAAATCAAACTATTCACAATAAACAAACCCTGAACAATGAGTCTGTGGGGA
Tarsier         TAAAACTTACAAGGCAACTTCTTCAGAATAAATAAGCTCTAAACAACGAGTCTATGAGGT
Bushbaby        TAAAATTTGTAAGGCAAATTTTTCAGAATAAACAAGCTCTAAACAATGAGTCTCCAAGGA
Lemur           ------------------------------------------------------------

Human           GCTGCCACTGAACTT-TAAGTTGCTCAAACACACTAACTGTGGAAG----AGTAGTACAA
Chimpanzee      GCTGCCACTGAACTT-GAAGTTGCTCAAACAC----ACTGTGGAAG----AGTAGTACAA
Gorilla         GCTGCCACTGAACTT-TAAGTTGCTCAAACACACTAACTGTGGAAG----AGTAGTACAA
Orangutan       GCTGCCACTGAACTT-GAAGTTGCTCAAACACACTAACTGTGGAAG----AGTAGTACAA
Gibbon          GATGCCACTGAACTT-TAAGTTGCTCAAACACACTAACTGTGGAAG----AGTAGTACAA
Baboon          GTTGCCACTGAACTT-TAAGTTGCTCGAACACACTAACTGTGGAAG----AGTAGTACAA
Macaque         GTTGCCACTGAACTT-TAAGTTGCTCGAACACACTAACTGTGGAAG----AGTAGTACAA
Rhesus          GTTGCCACTGAACTTTTAAGTTGCTCGAACACACTAACTGTGGAAG----AGTAGTACAA
Marmoset        GCTGCTACTGAACTT-TAAGTTGCTCAAACATACTAACTGTGGAAG----AGCAGTACAA
Squirrel_monkey GCTGCTACTGAACTT-TAAGTTGCTCAAACATACTAATTGTGGAAG----AGCAGTACAA
Tarsier         ATTGCCACTGAGCCTCAGCTCCTCACACATTG--ACACCAACCGTGGAAGAGCCGTGCTA
Bushbaby        GCTGGCACTGAACTCCCTAGTTTCTCATACGCCTCCGCTAACGATGGAAGGACTGTACAA
Lemur           ------------------------------------------------------------

Human           ATGAC---AAGTAGGCTTCCCACCAAGCAAAACTAGAAAATCAAAATTTGT-----TTCC
Chimpanzee      ATGAC---AAGTAGGCTTCCCACCAAGCAAAACTAGAAAATCAAAATTTGT-----TTCC
Gorilla         ATGAC---AAGTAGGCTTCCCACCAAGCAAAACTAGAAAATCAAAATTTGT-----TTCC
```

```
Orangutan       ATAAC---AACTAGGCTTCCCACCAAGCAAAACTAGAAAATCAAAATTTGT-----TCCC
Gibbon          ATGAC---AGGTAGGCTTCCCACCAAGCAAAACTAGAAAATCAAAATTTGT-----TTCC
Baboon          ATGAC---AAGTAGGCTTCCCACCAAGCAAAACTAGAAAATAAAAATTTGA-----TTCC
Macaque         ATGAC---AAGTAGGCTTCCCACT----------AGAAAATAAAAATTTGA-----TTCC
Rhesus          ATGAC---AAGTAGGCTTCCCACT----------AGAAAATAAAAATTTGA-----TTCC
Marmoset        ATGAC---AAGTAGGCTTCCCACCAAGCAAAACTAGAAAATCAAAATTTGT-----TTCC
Squirrel_monkey ATGAC---AAGTAGGCTTCCCACCAAGCAAAACTAGAAAATCAAAATTTGT-----TTCC
Tarsier         AT----GGCACCAGGATTCCCACCAGGCAGAGCTAGAAAATCTGTGCAGCA-----TGCT
Bushbaby        ATGACTGTTCGCAGGCTTCCCACCAGGCAAAACTCAAAAATCAATGCATTTTTTTCCAGA
Lemur           ------------------------------------------------------------

Human           AGCAGTAGCACATCTTCAAGTCCCTCCTCCTCACTTCCTCCTTTCTT---TTTTCTTTGC
Chimpanzee      AGCAGTAGTACATCTTCAAGTCCCTCCTCCTCACTTCCTCCTTCCTT---TTTTCTTTGC
Gorilla         AGCAGTAGCACATCTTCAAGTCCCTCCTCCTCACTTCCTCCTTTCTT---TTTTCTTTGC
Orangutan       AGCAGTAGCACATCTTCAAGTCCCTCCTCCTCACTTCCTCCTTTCTT---TTTTCTTTGC
Gibbon          AGCAGTAGCACATCTTCAAGTCCCTCCTCCTCACTTCCTCCTTTCTT---TTTTCTTTGC
Baboon          AGCAGTAGCACATCTTCAAGTCCCTCCTCCTCACTTCCTCCTTTCTTAGACATTCTTTGT
Macaque         AGCAGTAGCACATCTTCAAGTCCCTCCTCCTCACTTCCTCCTTTCTTAGACATTCTTTGT
Rhesus          AGCAGTAGCACATCTTCAAGTCCCTCCTCCTCACTTCCTCCTTTCTTAGACATTCTTTGT
Marmoset        AGCAGTAGTACATCTTCAAGTCCTTCCTTTTCACTTCCTTCTTTGAC---A-TTCTTTGT
Squirrel_monkey AGCAGTAGCACATCTTCAAGTCCTTCCTTTTCACTTCCTTCTTTGAC---A-GTCTTTGT
Tarsier         TTCAGCAACCCATCTTCAAGTC-CTTTGCCCTACTTCCTCCTTTGTTTGATATTCTTCCT
Bushbaby        AGCAGCAGCCTGTCTTCAAATCCCTGTGCTTTACTTCTTCCTTTCTGTGACATTCTTCCT
Lemur           ------------------------------------------------------------

Human           CAAAACAAAACAGATCTACTTAAAAGGCCAAAGGCCACTGGAGAAA--GAGTTTATGATC
Chimpanzee      CAAAACAAAACAGATCTACTTAAAAGGCCAAAGGCCACTGGAGAAA--GAGTTTATGATC
Gorilla         CAAAACAAAACAGATCTACTTAAAAGGCCAAAGGCCACTGGAGAAA--GAGTTTATGATC
Orangutan       CAAAACAAAACAGATCTACTTAAAAGGCCAAAGGCCACTGGAGAAA--GAGTTTATGATC
Gibbon          CAAAACAAAACAGATCTACTTAAAAGGCCAAAGGCCACTGGAGAAA--GAGTTTATGATC
Baboon          CAAAACAAAACAGATCTACTTAAAAGGCCAAAGGCCACTGGAGAAA--GAGTTTATGATC
Macaque         CAAAACAAAACAGATCTACTTAAAAGGCCAAAGGCCACTGGAGAAA--GAGTTTATGATC
Rhesus          CAAAACAAAACAGATCTACTTAAAAGGCCAAAGGCCACTGGAGAAA--GAGTTTATGATC
Marmoset        CAAAACAAAACATATATACTTAAAAGGTTAACGGCCACTGGAGAAA--CAGTTTATGATC
Squirrel_monkey CAAAACAAAACAGATCTACTTAAAAGTTCAAAGGCCACTGGAGAAA--TAGTTCATGATC
Tarsier         CAAAACAAAACAGATCAACAAGAAATCA--AAGACCACTCAAGAAAGGGGCATGATTATA
Bushbaby        CAAGTCAATAA---------TAAAAGGTCAAAGTTCACTCAAAAAAGTAAATTCATGCTA
Lemur           ------------------------------------------------------------

Human           ------AGACAACCCAGGTGTCCTATGTCTAAAAGGGAAATACAGGTCTTGAAATGAGAT
Chimpanzee      ------AGACAACCCAGGTGTCCTATGTCTAAAAGGGAACTACAGGTCTTGAAATGAGAT
Gorilla         ------AGACAACCCAGGTGTCCTATGTCTAAAAGGGAAATACAGGTCTTGAAATGAGAT
Orangutan       ------AGACAACCCAGGTGTCCTATGTCTAAAAGGGAAATACAGGTCTTGAAATGAGAT
Gibbon          ------AGACAACCCAGGTGTCCTATGTCTAAAACGGAAGTACCGGTCTTGAAATGAGAT
Baboon          ------AGACAACCCAGGTGTCCTGTGTCTATAAGGGAAATACAGGTCTTGAAATGAGAT
Macaque         ------AGACAACCCAGGTGTCCTGTGTCTATAAGGGAAATACAGGTCTTGAAATGAGAT
Rhesus          ------AGACAACCCAGGTGTCCTGTGTCTATAAGGGAAATACAGGTCTTGAAATGAGAT
Marmoset        ------AGAAAATACAGGTGTCCTGTGTCTTAAAGAGAAATACAGGTCTTGAAATGAGAC
Squirrel_monkey ------AGAAAACACAGGTATCCTGTGTCTTAAAGAGAAATACAGATCTTGAAATGAGAC
Tarsier         CAACATAGGTG-TTTTGTGTCTAAAGGGAGAAAAGGGAAATTCAGGTCTCTAGATGAGAT
Bushbaby        AGACAAACAGGTGTTGGGTGTCTAACGGAGAAAAGGGAAACAGTGGTCTTAAAATGAGAT
Lemur           ------------------------------------------------------------

Human           AGACTGTGAAAATAATGAATTAGTTAGAATTCATAGGGAAAGAACTTCATTCCTTAACAC
Chimpanzee      AGACTGTGAAAATAATGAATTAGTTAGAATTCACAGGGAAAGAACTTCATTCCTTAACAC
Gorilla         AGACTGTGAAAATAATGAATTAGTTAGAATTCATAGGGAAAGAACTTCATTCCTTAACAC
Orangutan       AGACTGTGAAAATAATGAATTAGTTAGAATTCATAGGGAAAGAACTTCATTCCTTAACAC
Gibbon          AGACTGTGAAAATAATGAATTAGTTAGAATTCATAGGGAAAGAACTTCATTCCTTAACAC
Baboon          AGACTG--GAAATAATGAATTAATTAGAATTCACAGGGAAAGAACTTCATTCCTTAGCAT
Macaque         GGACTG--GAAATAATGAATTAGTTAGAATTCACAGGGAAAGAACTTCATTCCTTAGCAT
Rhesus          AGACTG--GAAATAATGAATTAGTTAGAATTCACATGGAAAGAACTTCATTCCTTAGCAT
Marmoset        AGACT--GAAAATAATGAATTAGTTAGAATTCATAGGGAAGGAACTTCATTCCTCTACAC
Squirrel_monkey AGACTGTGAAAATAATGAATTCGTTAGAATTCATAGGGAAGGAACTTCATTCCTTAACAC
Tarsier         GGACTGTGAAGGTAATAAATTAGTTAGAATGGGTAGAAAAAGAACTTTGCTCCTTAATAC
Bushbaby        AGACTGTGAAGATAATAAATTAGCTAGAACTGACAGGAAAGAACA-CGATGCCTTAACAC
Lemur           ------------------------------------------------------------

Human           TTATCATCATGGTATTGTTTCTGCGGTGTGTTATTACTTCAGTAACTTCTTCCTGTAATG
Chimpanzee      TTATCATCATGGTATTGTTTCTGTGGTGTATTATTACTTCAGTAGCTTCTTCCTGTAATG
Gorilla         TTATCATCATGGTATTGTTTCTGTGGTGTGTTATTACTTCAGTAACTTCTTCCTGTAATG
Orangutan       TTATCATCATGGTATTGTTTCTGTGGTGTATTATTACTTCAGTAACTTCTTCCTGTAATG
Gibbon          TTATCATCATGGTATTGTTTCTGTGGTGTATTATTACTTCAGTAACTTCTTCCTGTAATG
Baboon          TTATCTTCATGGTATTGTTTCTGTGGTGTATTAGTACTTCAGTAACTTATTCCTGTAATG
```

```
Macaque        TTATCTTCATGGTATTGTTTCTGTGGTGTATTAGTACTTCAGTAACTTATTCCTGTAATG
Rhesus         TTATCTTCATGGTATTGTTTCTGTGGTGTATTAGTACTTCAGTAACTTATTCCTGTAATG
Marmoset       TTGTCATCATGGTATTGTTTCTGTGGTATATTATTACTTCAGTAACTTCTTCCTGTAATG
Squirrel_monkey TTGTCATCATGGTATTGTTTCTATGGCATATTATTACTTCAGTAACTTCTTCCTGTAACG
Tarsier        TTTCCACCATGGTATTGTTTGTGTGGTATATTATTACTTCAGGATCCTCTTCCTATAATG
Bushbaby       TCTCCACCATGGTGC-----------------GTCTCTTCAGTATCATCTTCCCATAACC
Lemur          ------------------------------------------------------------

Human          ATCTAGGTTACATGTAGGTATGTATTTATCACAATAATCTGATGGCTGTCTAATAACTAG
Chimpanzee     ATCTAGGTTACATGTAGGTATGTATTTATCACAATAATCTGATGGCTGTCTAATAACTAG
Gorilla        ATCTAGGTTACATGTAGGTATGTATTTATCACAATAATCTGATGGCTGTCTAATAACTAG
Orangutan      ATCTAGGTTATACGTAGGTATGTATTTATCACAATAATCTGATGGCTGTCTAATAACTAG
Gibbon         ATCTAGGTTACATGTAGGTATGTATTTATCACAATAATCTGATGGCTGTCTAATAACTAG
Baboon         GTCTAGGTTACATGTAGGCATGTATTTATCACAATAATCTGATGGCTGTCTAATAACTAG
Macaque        ATCTAGGTTACATGTAGGCATGTATTTATCACAATAATCTGATGGCTGTCTAATAACTAG
Rhesus         ATCTAGGTTACATGTAGGCATGTATTTATCACAATAATCTGATGGCTGTCTAATAACTAG
Marmoset       ATCTAGGTTACATGTAGGTATATATTTATCACAATAATCTGATGGCTGTCTAATAACTAG
Squirrel_monkey AACTAGGTTACATGTAGGTATATATTTATCACAATAATCTGATGGCTGCCTAATAACTAG
Tarsier        ATCTAGGTTAGACGTGGGTATAATTGATTTCAGAAATGTGTTCTTAGTAGATTGTTATA
Bushbaby       ATCTAAGTTCGATGTGGGTATAAATCATTTTCTGGATTA---------------------
Lemur          -----------------------------------------------GTAGATATTCTAT

Human          TCACATACATGACATCTCTACTTCATAATAGTAGGAGCAACTT-ACTCTAATCTGACTAA
Chimpanzee     TCACATACATGACATCTCTACTTCATAATAGTAAGAGAAACTT-ACTCTAATCTGACTAA
Gorilla        TCACCTACATGACATCTCTACTTCATAATAGTAGGAGAAACTT-ACTCTAATCTGACTAA
Orangutan      TCACATACATGACATCTCTACTTCATAATAGTAGGAGAAACTT-ACTCCAATCTGACTAA
Gibbon         TCACATACATGACATCTCTACTTCATAATAGTAGGAGAAACTT-ACTCTAATCTGACTAA
Baboon         TCACATACATGACTAGTAACATACATCTCTACTTCATATAGTAGGAGAAACTTACTCTAA
Macaque        TCACATACATGA-----------CATCTCTACTTCATATAGTAGGAGAAACTTACTCTAA
Rhesus         TCACATACATGA-----------CATCTCTACTTCATATAGTAGGAGAAACTTACTCTAA
Marmoset       TCACATGCATGGCATCTCTACTTCATAATAGTAGGAGAAACTT-ACTCTAATCTGACTAA
Squirrel_monkey TCACGTGCATGACATCTCTACTTCATAATAGTAGGAGAAACTT-ACTCTAATCTGACTAA
Tarsier        TCACAATAAACTGATGG-------------------------------------------
Bushbaby       ------------------------------------------------------------
Lemur          CACAATAAACCAATAAA-------------------------------------------

Human          AGTAAAATATGATACATAAGCCTTTTTAATTCTGCAATAACCCATTCATCTTTATTATTA
Chimpanzee     AGTAAAATATGATACATAAGCCTTTTTAATTCTGCAATAACCCATTCATCT----TATTA
Gorilla        AGTAAAATATGTTACATAAGCCTTTTTAATTCTGCAATAACCCATTCATCTTTATTATTA
Orangutan      ATTAAAATATGATACATAAGCCTTTTTAATTCTGCAATAACCCATTCATCTTTATTATTA
Gibbon         AGTAAAATATGATACATAAGCCTTTTTAATTCTGCAATAACCCATTCATCTTTACTATTA
Baboon         AGTAAAATATGATACATATGCCTTTTTAATTCTGCAATAACCCATTCATCTTTATTAATA
Macaque        AGTAAAATATGATACATATGCCTTTTTAATTCTGCAGTAACCCATTCATCTTTATTAATA
Rhesus         AGTAAAATATGATACATATGCATTTTTAATTCTGCAATAACCCATTCATCTTTATTAATA
Marmoset       AGTAAAATATGATAATA-TGCCTTTTTAATTCTGCAATAACCCATTTATCTTTATTATTA
Squirrel_monkey AGTGAAATATGATACATATGCCTTTTTAATTCTGCAATAACCCATTTATCTTTATTATTA
Tarsier        ------------------------------------------------------------
Bushbaby       ------------------------------------------------------------
Lemur          ------------------------------------------------------------

Human          ATAGATTTTTAAAA----TTACCAGTACGTATTTTTCTGAAATGTGTGCAATCAATCCTT
Chimpanzee     ATAGATTTTTAAAA----TTACCAGTACGTATTTTTCTGAAACGTGTGCAATCAATCCTT
Gorilla        ATAGATTTTTAAAA----TTACCAGTACGTATTTTTCTGAAACGTGTGCAATCAATCCTT
Orangutan      ATAGATTTTTAAAA----TTACCAGTACATATTTTTCTGAAACGTATGCAATCAATCCTT
Gibbon         AAATAGATTTTTAAA-ATTACCAGTACGTATTTTTTCTGAAACGTATGCAATCAATCCTT
Baboon         GATTTTTTAA-------ATTACCAATATGTATTTTTCTGAAATGTATGCAATCAATCCTT
Macaque        GATTTTTAAA-------ATTACCAATATGTATTTTTCTGAAATGTATGCAATCAATCCTT
Rhesus         GATTTTTAAA-AAAAAAATTACCAATATGTATTTTTCTGAAATGTATGCAATCAATCCTT
Marmoset       ATAGTTAGATTTTTAAAGTTACCAACACATATTTTTCTGAAATATATGCAATCAGTACTT
Squirrel_monkey ATAGTTAGATTTTTTTTTTTT---------------------------------------
Tarsier        -----------------CTCT---------------------------------------
Bushbaby       ----------------CGTTCTTAGTAGATATTATATCACAA-----TAAACCAATAGCT
Lemur          -----------------CTCT---------------------------------------

Human          TGTCAATTCTTTACTACTACGATCTGAAGAAAGTTGCAATCCAAAGAGAATAAGGGTTAC
Chimpanzee     TGTCAATTCTTTACTACTATGATCTGAAGAAAGTTGCAATCCACAGAGAATAAGGGTTAC
Gorilla        TGTCAATTCTTTACTACTATGATCTGAAGAAAGTTGCAATCCAAAGAGAATAAGGGTTAC
Orangutan      TGTCAATTCTTTACTACTACGATCTGAAGAAAGTTGCAATCCAAAGACAATAAGGGTTAC
Gibbon         TGTCAATTCTTTACTATG-ATC--TGAAGAAAGTTGCAATCCAAAGAGAGTAAGGGTTAC
Baboon         TGTCAATTCTTTACTACTATGATCTGAAGGAAGTTGCAATTCAAAGAGAGTAAGGTTTAC
Macaque        TGTCAATTCTTTACTACTATGATCTGAAGGAAGTTGCAATTCAGAGAGAGTAAGGTTTAC
Rhesus         TGTCAATTCTTTACTACTATGATCTGAAGGAAGTTGCAATTCAGAGAGAGTAAGGTTTAC
Marmoset       TGTCAGTTCTTTACTACT-ATGATCTAAAAAAGTTGCAATACAAGGAGAATAAGGATTAC
```

```
Squirrel_monkey ------------------------------------------------------------
Tarsier         ------CTAATAACTAGTCACATACATGAC------------------------------
Bushbaby        TT----CTAATTACCAGTCACATGTAGCAGCTCTATTTCTAAATAGAAGAAATGTGTTGT
Lemur           ------CTAATAACTAGTCACATATGTGGC------------------------------

Human           AATTATACTTGAAGATGAGAGTGCTAAAAATCTGTGATATGTTTGAAAACCTA-------
Chimpanzee      AATTATACTTGAAGATGAGAGTACTAAAAATCTGTGATATGTTTGAAAACCTA-------
Gorilla         AATTATGCTTGAAGATGAGAGTACTAAAAATCTGTGATATGTTTGAAAACCTA-------
Orangutan       AATTATACTTGAAGATGAGAGTACTAAAAATCTGTGATATGTTTGAAAACCTA-------
Gibbon          AATTATACTTGAAGATGAGAGTACTAAAAATCTG--TGATGTTTGAAAACCTA-------
Baboon          AATTATACTTGAAGATGAGAGTACTAAACATCTGTGATATATCTGAAAACCTAGCCAATG
Macaque         AATTATACTTGAAGATGAGAGTACTAAACATCTGTGATATATCTGAAAACCTAGCCAATG
Rhesus          AATTTTACTTGAAGATGAGAGTACTAAACATCTGGGATATATCTGAAAACCTAGCCAATG
Marmoset        AATTATACTTGAAGATGAGCATACTAAAAATCTATGATATGTTTGAAAACCTA-------
Squirrel_monkey ------------------------------------------------------------
Tarsier         -----------------------------ATCTCTACTT---------------------
Bushbaby        AATTTGACTTAACTCTGACTCA--------TATGCCTTTTTATTATATAATAATAACAT
Lemur           -----------------------------ATCTCTACTT---------------------

Human           ------------------------------------------------------------
Chimpanzee      ------------------------------------------------------------
Gorilla         ------------------------------------------------------------
Orangutan       ------------------------------------------------------------
Gibbon          ------------------------------------------------------------
Baboon          GGTGACTACTGCTTTCAGTACCTGACTTGCTGCCTAGTAAACTAAACTGGGTGCCTAAAA
Macaque         GGTGACTACTGCTTTCAGTACCTGACTTGCTGCCTAGTAAACTAAACTGGGTGCCTAAAA
Rhesus          GGTGACTACTGCTTTCAGTACCTGACTTGCTGCCTAGTAAACTAAACTGGGTGCCTAAAA
Marmoset        ------------------------------------------------------------
Squirrel_monkey ------------------------------------------------------------
Tarsier         --------------------------------CATATTAGTAAGAGAAACTTGCTCTAAT
Bushbaby        AT----------------------------------------------------------
Lemur           --------------------------------CATAATAGAAGGAGGAGCTTGTCCTAAT

Human           ------------------------------------------------------------
Chimpanzee      ------------------------------------------------------------
Gorilla         ------------------------------------------------------------
Orangutan       ------------------------------------------------------------
Gibbon          ------------------------------------------------------------
Baboon          TTCCCTTTGTTTATTTTCAACTTCAATTATATATAAATTGAAAGATATTGTTTTAATATC
Macaque         TTCCCTTTGTTTATTTTCAACTTCAATTATATATAAATTGAAAGATATTGTTTTAATATC
Rhesus          TTCCCTTTGTTTATTTTCAACTTCAATTATATATAAATTGAAAGATATTGTTTTAATATC
Marmoset        ------------------------------------------------------------
Squirrel_monkey ------------------------------------------------------------
Tarsier         TTG----------------ACTAAA-----GTAAAATATGACACATATGCCTTTTTTAT-
Bushbaby        ------------------------------TCG--T-C-T-T--T-AC--T-T-G-T-T-
Lemur           TTG----------------ACTA----------AATTATGACTCATATGCCTTTTTTAA-

Human           ------------------------------------------------------------
Chimpanzee      ------------------------------------------------------------
Gorilla         ------------------------------------------------------------
Orangutan       ------------------------------------------------------------
Gibbon          ------------------------------------------------------------
Baboon          CTTTCAGCTACTCTAAGAATACATAACTGAATATAGAACAAGCTATATAAAGTACAAGGC
Macaque         CTTTCAGCTACTCTAAGATTACATAACTGAATATAGAACAAGCTATATAAAGTACAAGGC
Rhesus          CTTTCAGCTACTCTAAGATTACATAACTGAATATAGAACAAGCTATATAAAGTACAAGGC
Marmoset        ------------------------------------------------------------
Squirrel_monkey ------------------------------------------------------------
Tarsier         ------TCTAC---AATAACTCATT--------C---------A-TCTTTACTAGTTAGA
Bushbaby        A-G-A-T-T-T-C-C-T-T-T-A-A--T-G-G-C-T-A-A-C-A-C-T-T-T-T-T-T-A
Lemur           ------TGTAT---AATAACATATT--------C---------A-TCTTTACTAGTTAGA

Human           ------------------------------------------------------------
Chimpanzee      ------------------------------------------------------------
Gorilla         ------------------------------------------------------------
Orangutan       ------------------------------------------------------------
Gibbon          ------------------------------------------------------------
Baboon          TTAGTAAAAGAAACATCAGGCTAATACCTGAGTTTTCATACTTAGTACAAAAGTCTAAAA
Macaque         TTAGTAAAAGAAACATCAGGCTAATACCTGCGTTTTCATACTTAGTATAAAAGTCTAAAA
Rhesus          TTAGTAAAAGAAACATCAGGCTAATACCTGAGTTTTCATACTTATGGCTTAAACTTCTCA
Marmoset        ------------------------------------------------------------
Squirrel_monkey ------------------------------------------------------------
Tarsier         TTTTTAAGAA----T-T-----------------------------A-CCAA--CAACAA
Bushbaby        -A-A-C-C-T--A-T-G-C-A-A-T-C-A-A-T-C-C-T-T-T-G-T-C-A-G-T--T-C
```

```
Lemur           TTTTTTAAAC----T-A-----------------------------C-CAACACTTTTGT

Human           ------------------------------------------------------------
Chimpanzee      ------------------------------------------------------------
Gorilla         ------------------------------------------------------------
Orangutan       ------------------------------------------------------------
Gibbon          ------------------------------------------------------------
Baboon          TATTTTTTACCCCCGACCTGAATATGGCTTAAACTTCTCATACAATGTACTACTAGCTTA
Macaque         TATTTTTTACCCCCGACCTGAATATAGCTTAAACTTCTCATACAATGTACTACTAGCTTA
Rhesus          TA----------------------------------------CAATGTACTANNNNNNNN
Marmoset        ------------------------------------------------------------
Squirrel_monkey ------------------------------------------------------------
Tarsier         TTTTTCTAAAATCTGGGC-AATCAACCCTTTGTCAGTTCTTT------ACTAC-------
Bushbaby        -T-T-T-A-G-T-A-G-T-A-T-T-A-T-T-T-G-A-A-T-T--T-A-A-G-A-A-G-C-
Lemur           TTTTTTCCAAATCTATGC-AATCAATCCTTTTTCTGCTCTTT------GCTAG-------

Human           ------------------------------------------------------------
Chimpanzee      ------------------------------------------------------------
Gorilla         ------------------------------------------------------------
Orangutan       ------------------------------------------------------------
Gibbon          ------------------------------------------------------------
Baboon          ATAGTCATTTTAGAATTAATTATTGCAAGTAGTTCATGGAGATAAATGCATTTTTATTCA
Macaque         ATAGTCATTTTAGAATTAATTATTGCAAGTAGTTCATGGAGATAAATGCATTTTTATTCA
Rhesus          NNNNNNNNNNNNNNNNNNNNNNN-------------------------------------
Marmoset        ------------------------------------------------------------
Squirrel_monkey ------------------------------------------------------------
Tarsier         ----TATTATTTGATTTTAAGAAAGT-TGCAATGCAAAGAGA-------ATATAGGT---
Bushbaby        T-G-C-A-A-T-T-C-A-A-A--G-A-G-A-A-T-A-T-A-G-G-T-C-A-C-A-A-T-T
Lemur           ----TATTATTTGAATTTAAGAAAGT-TGCAATTCAAAGAGA-------ATATAGGT---

Human           ------------------------------------------------------------
Chimpanzee      ------------------------------------------------------------
Gorilla         ------------------------------------------------------------
Orangutan       ------------------------------------------------------------
Gibbon          ------------------------------------------------------------
Baboon          ACTTTAAGTAGTTTGTTTCATTGTTTTGACTATAAATAAGCAATCTCATCAATGATCACT
Macaque         ACTTTAAGTAGTTTGATTCATTGTTTTGATTATAAATAAGCAATCTCATCAATGATCACT
Rhesus          -----------------------------------------NNNNNN----------NNN
Marmoset        ------------------------------------------------------------
Squirrel_monkey ------------------------------------------------------------
Tarsier         ----TAC-AATTATATTTG-------------AAGATGAGAGTACTAAAA-A---T----
Bushbaby        -A--T-A-A-T-T-G-A-A-G-G-G-G-A-G-A-G-T-A-G-T-G-A-A--A-A-T-A-T
Lemur           ----AAC-AAAAATAATTG-------------AAGATGAGAATACTAACA-T---T----

Human           ------------------------------------------------------------
Chimpanzee      ------------------------------------------------------------
Gorilla         ------------------------------------------------------------
Orangutan       ------------------------------------------------------------
Gibbon          ------------------------------------------------------------
Baboon          TCCAAGTTACAGTTTAATATCTTAATCATTACTCACAAAGCATTATAGGATTATTAATAA
Macaque         TCCAAGTTACAGTTTAATATCTTAATCATTACTCACAAAGCATTATAGGATTATTAATAA
Rhesus          NNNNN-------------------------------------------------------
Marmoset        ------------------------------------------------------------
Squirrel_monkey ------------------------------------------------------------
Tarsier         ------------------------------------------------------------
Bushbaby        ------------------------------------------------------------
Lemur           ------------------------------------------------------------

Human           ------------------------------------------------------------
Chimpanzee      ------------------------------------------------------------
Gorilla         ------------------------------------------------------------
Orangutan       ------------------------------------------------------------
Gibbon          ------------------------------------------------------------
Baboon          AGCTCCCTAAT----AGTATTATTATTTTCACTCTTTCCAAGGAGTCAGTTGTCAATATA
Macaque         AGCTCCCTAATAGATAATATTATTATTTTCACTCTTTCCAAGGAGTCAGTTGTCAATATA
Rhesus          --------------------------------NNNNNNNNNNNNNNNNNNNNNNNNNNNN
Marmoset        ------------------------------------------------------------
Squirrel_monkey ------------------------------------------------------------
Tarsier         ------------------------------------------------------------
Bushbaby        ------------------------------------------------------------
Lemur           ------------------------------------------------------------

Human           -----------------------------------------GCAAATGGGTGACTCATAC
```

```
Chimpanzee     -----------------------------------------GCAAATGGGTGACTCATAC
Gorilla        -----------------------------------------GCAAATGGGTGACTCATAC
Orangutan      -----------------------------------------GAAAATGGGTGACTCATAC
Gibbon         -----------------------------------------GCAAATGGGTGACTCATAC
Baboon         AAAAATATGTTAATATAAGAATAGTAAGGAGCATTTTATAAATAAACTGTTAAAAGCAAA
Macaque        AAAAATATGTTAATATAAGAATAGTAAGGAGCATTTTATAAATAAACTGTTAAAAGCAAA
Rhesus         NNNNNNNNNNNNNNNTAAGAATAGTAAGGAGCATTTTATAAATAAACTGTTAAAAGCAAA
Marmoset       -----------------------------------------GCAAATGGGTGACTCATAC
Squirrel_monkey ------------------------------------------------------------
Tarsier        ------GTAT-----------TGATACATTAGCAAATCA----------TTGACTCTTAC
Bushbaby       ---------------------ATTGAGCCATTGGAAGACCTAGTGAATGGTTGACTCTTA
Lemur          ------CTAT-----------TGATACACTGGAAAACCTAGCAAATGGTTGACTCATTAG

Human          TTTCATTACCTTACTTGCTGCCTAGTAAACTAAACTGGGTGCCTAAAATTCCCTTTGTTT
Chimpanzee     TTTCATTACCTTACTTGCTGCCTAGTAAACTAAACTGGGTGCCTAAAATTCCCTTTGTTT
Gorilla        TTTCATTACCTTACTTGCTGCCTAGTAAACTAAACTGGGTGCCTAAAATTCCCTTCGTTT
Orangutan      TTTCATTACCTTACTTGCTGCCTAGTAAACTAAACTGAGTGCCTAAAATTCCCTTTGTTT
Gibbon         TTTCATTACCTTACTTGCTGCCTAGTAAACTAAACTGGGTGCCTAAAATTCCCTTT----
Baboon         TTTCATTACCTTACTTGCTGCCTAGTAAACTAAACTGGGTGCCTAAAATTCCCTTTGTT-
Macaque        TTTCATTACCTTACTTGCTGCCTAGTAAACTAAACTGGGTGCCTAAAATTCCCTTT----
Rhesus         TTTCATTACCTTACTTGCTGCCTAGTAAACTAAACTGGGTGCCTAAAATTCCCTTT----
Marmoset       TTTCGTTACTTTACTTGCTGCCTAGTAAACTAAACTGAGTGCCTAAAATTCCCTTTCTGT
Squirrel_monkey ------------------------------------------------------------
Tarsier        TTTCGTTACCTTACTTGATGCCTAGTAAATTAAATTGGGTTCCTAAAACTCCTTTTCTAT
Bushbaby       TTTCATTATCTCACTGGATGCCTAGTGAACTAATCTGGGAATCTAAAATTCCCTTTGTAT
Lemur          TTTCATTATTTTACTTGGTGCCTAATAAACTAAACTGGGTTCCTAAAATTCCCTTTGTAT

Human          --ATTTTCAACTTCAATTATATATGCACAGTATTTTAATTACTTAGCATCTATTGTTCCA
Chimpanzee     --ATTTTCAACTTCAATTATATATGCACAGTATTTTAATTACTTAGCATCTATTGTTCCA
Gorilla        --ATTTTCAACTTCAATTATATATGCACAGTATTTTAATTACTTAGCATCTATTGTTCCA
Orangutan      --GTTTTCAACTTCAATTACATATGCACAGTATTTTAATTACTTAGCCTCTATTGTTCTA
Gibbon         --ATTTTCAACTTCAATTATATATGCACAGTATTTTAATTACTTAGCCTCTATTGTTCTA
Baboon         TATTTTTCAACTTTAATTATATATGCACAGTATTTTAATTACTTAGCCTCTATTGTTCCA
Macaque        -ATTTTTCAACTTTAATTATATATGCACAGTATTTTAATTACTTAGCCTCTATTGTTCCA
Rhesus         -ATTTTTCAACTTTAATTATATATGCACAGTATTTTAATTACTTAGCCTCTATTGTTCCA
Marmoset       TTATTTTCAACTTCTATTATATAGGCACAGTATTTTAATTATTTAGCCTCTATTGTTCTA
Squirrel_monkey ------------------------------------------------------------
Tarsier        TTATTCTCAACTTCAATT----ATACATACTATTTTAATTATCTAGCCTCTATTGTTCTA
Bushbaby       TTATTCTCAAGTTCAAATT---ACATACACTATGTTAATTCTGCAGCCTCTATTGTGCTA
Lemur          TTATTCTCAACTTCAATT----ATATGTACTATTTTAATTATCCAGCCTCTATTATACTA

Human          GGTGCTTAGATTTTTCCACATCAAAATTCTCCAGTTTCCATTGTGTTATTTCCTGTTATA
Chimpanzee     GGTGCTTAGATTT-TCCACATCAAAATTCTCCAGTTTCCATTGTGTTATTTCCTGTTATA
Gorilla        GGTGCTTAGATTTTTCCACATCAAAATTCTCCAGTTTCCATTGTGTTATCTCCTGTTATA
Orangutan      GGTGCTTAGATTTTTCCACATCAAAATTCTCCAGTTTCCATTGTGTTATTTCCTGTTATA
Gibbon         GGTGCTTAGATTTTTCCACATCAAAATTCTCCAGTTTCCATTGTGTTATTTCCTGTTATA
Baboon         GATGCTTAGATTTTTCCACATCAAAATTCTCCAGTTTCCATTGTGTTATTTCCTATTATA
Macaque        GATGCTTAGATTTTTCCATATCAAAATTCTCCAGTTTCCATTGTGTTATTTCCTATTATA
Rhesus         GATGCTTAGATTTTTCCATATCAAAATTCTCCAGTTTCCATTGTGTTATTTCCTATTATA
Marmoset       GGTGCTTATATTTTTCTGCATCACAATTCTCCAGTTTCCATTGTATTATTTCCTGTTTTA
Squirrel_monkey ------------------------------------------------------------
Tarsier        AGTTCTTAAATTTTTCCATATCACAATTCTCCAATTTCCATTATGTTATTTCCTGTGATA
Bushbaby       GTTT-CTCAAATTTTTCCTATCACAATTCCCCAGTTTCCATTCTGTTATGTCCTGTTGTA
Lemur          AGTTCTTAGATTTTTCCACATCACAATTCTCCAGTTTCCATTGTGTTATTTCCTGTTGTG

Human          AGATTATTCAAAGTAGTTTTTTTCTTTATTGTTG---T-TTTTCAAAGGGAGGCAGACTG
Chimpanzee     AGATTATTCAAAGTAATTTTTTTCTTTATTGTTG---T-TTTTCAAAGGGAGGCAGACTG
Gorilla        AGATTATTCAAAGTAGTTTTTTTCTTTATTGTTG---T-TTTTCAAAGGGAGGCAGACTG
Orangutan      AGATTATTCAAAGTAGTTTTTTTCTTTATTGTTG---T-TTTTCAAAGGGAGGCAGACTG
Gibbon         AGATTATTCAAAGTAGTTTTTTTCTTTATTGTTG---T-TTTTCAAAGGGAGGCAGACTG
Baboon         AGATTATTCAAAGTAGTTTTTTTCTTTATTGTTG---C-TTTTCAAAGGGAGGCAGACTG
Macaque        AGATTATTCAAAGTAGTTTTTTTCTTTATTGTTG---T-TTTTCAAAGGGAGGCAAACTG
Rhesus         AGATTATTCAAAGTAGTTTTTTTCTTTATTGTTG---T-TTTTCAAAGGGAGGCAGACTG
Marmoset       AGATTATTCAAAGGAGGTTT--TCTTTATTTTTG---G-TTTTCAAAGGGAGGCAGACTG
Squirrel_monkey ------------------------------------------------------------
Tarsier        AGATTATTCAAAGTAGTTATTTATTTATTTACTGTTGTTTGTCAAA----------GGGA
Bushbaby       AGATTATTCGAAGTAGGGTTTTTTGTTGTTGTTGTTTG-TTTTTCAAAAGGAAGCAGGTG
Lemur          AGATTATTCAAAGTAGTTTTTTTTTTTTTTAATTGTTGTTTTTCAAAGGGAGGCAGGCTG

Human          ACCAGTCGTGTTTGGGGTTATTTCACAATGCTAGTTCCTTCCTGTATACAAATTCACACA
Chimpanzee     ACCAGTCGTGTTTGGGGTTATTTCACAATGCTAGTTCCTTCCTGTATACAAATTCACACA
Gorilla        ACCAGTCGTGTTTGGGGTTATTTCACAATGCTAGTTCCTTCCTGTATACAAATTCACACA
Orangutan      ACCAGTCGTGTTTGGGGTTATTTCACAATACTAATTCCTTCCTGTATACAAATTCACACA
```

```
Gibbon          ACCAGTCGTGTTTGGGGTCATTTCACAATGCTAGTTCCT-TCTGTATACAAATTCACACA
Baboon          ACCAGTTGTGTTTGGGGTTATTTCACAATGCTAGTTCCTTCCTGTATACAAATTCACACA
Macaque         ACCAGTTGTGTTTGGGGTTATTTCACAATGCTAGCTCCTTCCTGTATACAAATTCACACA
Rhesus          ACCAGTTGTGTTTGGGGTTATTTCACAATGCTAGTTCCTTCCTGTATACAAATTCACACA
Marmoset        ACTAGTCGTGCTTGGGGTTATTTCACAAAGCTAGTTTCTTCCTGTATACAAATTCACACA
Squirrel_monkey ------------------------------------------------------------
Tarsier         GATAGTGGTGTTTGGGGTTATTTCACAATGCCAGTTCCTTCCTGTATACACATTCACACA
Bushbaby        ACCAGTGGTGTTTGGGGTTATTTCACAATGCTGGTTCCTTCCTGCACACACATTCACACA
Lemur           ACCAGTTGTGTTTGGAGTTATTTCACAATGCTAGTTCCTTCCTGTATACACATTCACACA

Human           CGCACCCATATTCATACAAAT----GCTGGCCTTGGGTAAAAA-AAATAAGGTTGCCAAA
Chimpanzee      CGCACCCATATTCATACAAAT----GCTGGCCCTAGGTAAAAA-AAATAAGGTTGCCAAA
Gorilla         CGCACCCATATTCATACAAAT----GCTGGCCTTGGGTAAAAA-AAATAAGGTTGCCAAA
Orangutan       CGCACCCATATTCATACAAAT----GCTGGCCCTAGGTAAAAA-AAATAAGGTTGCCAAG
Gibbon          TGCACCCATATTCATACAAAT----GCTGGCCCTAGGTAAAAAAAAATAAGGTTGCCAAG
Baboon          GGCACCCGTATTCATACAAAT----GCTGGCCCTAGGTAAAA--AAATAAGGTTGCCAAG
Macaque         GGCACCCGTATTCATACAAAT----GCTGGCCCTAGGTAAAA--AAATAAGGTTGCCAAG
Rhesus          GGCACCCGTATTCATACAAAT----GCTGGCCCTAGGTAAAA--AAATAAGGTTGCCAAG
Marmoset        AGCACCCATATTCACACAAAA----GCTGGCCGTAGGTAAG--AAAATAAGGTTGCCAAG
Squirrel_monkey ------------------------------------------------------------
Tarsier         CGCTTCTATGCACACACATACACACACTGACCCTAGGTAA--GAAGATAAAATTGCCAAG
Bushbaby        TGCACTCACATACTCACGCAC----ACTGGCCCCAGGGAAGCAGACA---AAGTTACAAG
Lemur           TGCACTCATA----TACTCACGCACACTGGCCCTAGATAA--GAAGACAAAGTTGTCAAG

Human           CATTTGAAGTCTTCAGACCATTCTTTCTTTGTTCCTCCTTTGGGGAGCACCACCTCCCCT
Chimpanzee      CATTTGAAGTCTTCAGACCATTCTTTCTTTGTTCCTCCTTTGGGGAGCACCACCTCCCCT
Gorilla         CATTTGAAGTCTTCAGACCATTCTTTCTTTGTTCCTCCTTTGGGGAGCACCACCTCCCCT
Orangutan       CATTTGAAGTCTTCAGACCATTCTTTCTTTGTTCCTCCTTTGGGGCGCACCACCTCCCCT
Gibbon          CATTTGAAGTCTTCAGACCATTCTTTCTTTGCTCCTCCTTTGGGGAGCACC---------
Baboon          CATTTGAAGTCTTCAGACCATTCTTTCTTTGCTCCTCCTTTGGGGATCACCACCTCCCCT
Macaque         CATTTGAAGTCTTGAGACCATTCTTTCTTTGCTCCTCCTTTGGGGATCACCACCTCCCCT
Rhesus          CATTTGAAGTCTTGAGACCATTCTTTCTTTGCTCCTCCTTTGGGGATCACCACCTCCCCT
Marmoset        CATTTGAAGTCTTCAGACCATTCTTTCTTTGCCCTTCCTTTGGGGAGCACCACTTCCCCT
Squirrel_monkey ------------------------------------------------------------
Tarsier         AATTTGAAGCCTTCAGACCATTCTTTCCTTTCTCCTCCTTTTGGGAACATTGCCTCCCTT
Bushbaby        TATTTGAAATCTCCAGACCATTCTTCC---TTTCCTCTTCTCAGGAACAGGACTTCCCCT
Lemur           TATTTGAAATCTTCAGACCATTCT----TTTCTCCTGTTTTGGGGAGCATCGGCTCCCCT

Human           TCAATTATTCTGCATCCTGCATTTTCTCCTCTTCCTTACAAAGAGCCTGGTATCAGTCAC
Chimpanzee      TCAATTATTCTGCATCCTGCATTTTCTCCTCTTCCTTACAAAGAGCCTGGTATCAGTCAC
Gorilla         TCAATTATTCTGCATCCTGCATTTTCTCCTCTTCCTTACAAAGAGCCTGGTATCAGTCAC
Orangutan       TCAATTATTCTGCATCCTGCATTTTCTCCTCTTCCTTACAAAGAGCCTGGTATCAGTCAC
Gibbon          --------------------------ACCTCTTCCTTACAAAGAGCCTGGTATCAGTCAC
Baboon          TCAATTATTCTGCACTCTCCTTTTTCTCCTCTTCCTTACAAAGAGCCTGGAATCAGTCAC
Macaque         TCAATTATTCTGCATTCTCCTTTTTCTCCTCTTCCTTACAAAGAGCCTGGAATCAGTCAC
Rhesus          TCAATTATTCTGCATTCTCCTTTTTCTCCTCTTCCTTACAAAGAGCCTGGAATCAGTCAC
Marmoset        TCAATTATTCTGCATCCTGTATTTTCTCCTCTTCCTTACAAAGAGCCTGGTATCAGTCAC
Squirrel_monkey ------------------------------------------------------------
Tarsier         TCAATGATCCTGCATCCTGCCTTCTCCCCGCTTCCTTACAAAGAGCCTGGTGTCAGTCAC
Bushbaby        TCAATTATCCTGCATCCTGCATTCTCCTCTCTTCCCTACAAAGAGCCCGGTGTCAGTCAC
Lemur           TCAATTATTCTGCATCCTGCATTCTCCCCTATTCCTTACAAAGAGCCTGGTATCAGTCAC

Human           AGCTCCTGGCACTCCTGA-GTTACATGGCCTACTTCTGAGGCACGCAAAGATTTGGTAGC
Chimpanzee      AGCTCCTGACACTCCTGA-GTTACATGGCCTACTTCTGAGGCACGCATAGATTTGGTAGC
Gorilla         AGCTCCTGGCACTCCTGA-GTTACATGGCCTACTTCTGAGGCACGCATAGATTTGGTAGC
Orangutan       AGCTCCTGACACTCCTGA-GTTACATGGCCTACTTCTGAGGCACGCATAGATTTGGTAGC
Gibbon          AGCTCCTGACACTCCTGA-GTTACATGGCCTACTTCTGAGGCACACATAGATTTGGTAGC
Baboon          AGCTCCTGACACTCCTGA-GTTACATGGCCTACTTCTGAGGCACGCATAGACTTGGTAGC
Macaque         AGCTCCTGACACTCCTGA-GTTACATGGCCTACTTCTGAGGCACGCATAGACTTGGTAGC
Rhesus          AGCTCCTGACACTCCTGA-GTTACATGGCCTACTTCTGAGGCACGCATAGACTTGGTAGC
Marmoset        AGCTCCTGACACTCCTGA-GTTACATGGCCTACTTCTGAAGCACACATAGACTTGGTAGC
Squirrel_monkey ------------------------------------------------------------
Tarsier         AGGTCCCCATGCTCCTGAGTTACAATGGCCTACTTC------------------------
Bushbaby        ACTGCCCCTGCTTCTGAG-TTACAGTGGCCTAGTTCCGAGGCATGCACAGACT-------
Lemur           AGCTCCTCCTCCTG---AGTTACAGTGGCCTGATTCTGAGGCATGCATATACTTGGTAGC

Human           AAAGCAAAGGGAAGGGAAGTGGCAGACCTGTCTCTCAGCTGCCAGCTGCAATCTTTCTCG
Chimpanzee      AAAGCAAAGGGAAGGGAAGTGGCAGACCTGTCTCTCAGCTGCCAGCTGCAATCTTTCTCA
Gorilla         AAAGCAAAGGGGAGGGAAGTGGCAGACCTGTCTCTCAGCTGCCAGCTGCAATCTTTCTCG
Orangutan       AAAGCAAAGGGAAGGGAAGTGGCAGACCTGTCTCTCAGCTGC-AGCTGCAATCTTTCTCA
Gibbon          AAAGCAAAGGGAAGGGAAGTGGCAGACCTGTCTCTCAGCTGCCAGCTGCAATCTTTCTCG
Baboon          AAAGCAAAGGGAAAGGAAGCGACAGACCTGT--CTCAGCCGCCAGCTGCAATCTTTCTCG
Macaque         AAAGCAAAGGGAAAGGAAGCGACAGACCTGT--CTCAGCTGCCAGCTGCAATCTTTCTCA
```

```
Rhesus          AAAGCAAAGGGAAAGGAAGCGACAGACCTGT--CTCAGCTGCCAGCTGCAATCTTTCTCA
Marmoset        AAAGCGAAGGGAAGAAAATCGGCAGACCTGTCTCTCAGCTGGTAGCTGCAATCTTTCTGG
Squirrel_monkey ------------------------------------------------------------
Tarsier         ---------------------------------CTCAATGGGCGGTTGCAACCTTTCTGG
Bushbaby        ----------------CGGTAGCAGACTGGCCACTCAGCTGGCAGCTGTAATCTTCCCAG
Lemur           AAAGC--AAAAGGAAGCGGCAGACTTGCCTCTCAGCTGGCAGCGGCCACAATCTTTCTGG

Human           CACACCAGTGCCTCTCCTTCTTACAAGTTACGTCCTGTCGGTAGACGGACTGGCCCAACA
Chimpanzee      CACACCAGTGCCTCTCCTTCTTACAAGTTACGTCCTGTCGGTAGACGGACTGGCCCAGCA
Gorilla         CACACCAGTGCCTCTCCTTCTTACAAGTTACGTCCTGTCGGTAGACGGACTGGCCCAACA
Orangutan       CACACCAGTGCCTCTCCTTCTTACAAGTTACGTCCTGTCGGTAGACGGACTGGCTCAACA
Gibbon          CACACCAGTGCCTCTCCTTCTTACAAGTTACGTCCTGTCGGTAGACGGACTGGCCCAACA
Baboon          CACACCAGTGCCTCTCCTTCTTACAAGTTACATCCTGTCAGTAGACGGACTGGCCCAACA
Macaque         CACACCAGTGCCTCTCCTTCTTACAAGTTACATCCTGTCAGTAGACGGACTGGCCCAACA
Rhesus          CACACCAGTGCCTCTCCTTCTTACAAGTTACATCCTGTCAGTAGACGGACTGGCCCAACA
Marmoset        CACACCAGTGCCTCTCCTTCTTACAAGTTACATCCTGTCTCTAGACAGACTGGCCCAACA
Squirrel_monkey ------------------------------------------------------------
Tarsier         CATGCCAGTGCTTCTCTTTCTTACAAGTTACATCCTGCCTGAGACAGACAGGTTC--AAC
Bushbaby        CACAGCAGCGGTTCTCTCTCTTACAAGCTACATCCTGTCTGTAGACAGACCGGCCCAACA
Lemur           CACACCAGTGCTTCTCCTTCTTACAAGTTACATCCTGTCTGCAGATAGACTGGCCCAACA

Human           ACAAGGTTATATCTATTGAGGTTAAACTTGCCCCAAACAGTGTTTGTTGGAGCAGAGAGG
Chimpanzee      ACAAGGTTATATCTATTGAGGTTAAACTTGCCCCAAACACTGTTTGTTGGAGCAGAGAGG
Gorilla         ACAAGGTTATATCTATTGAGGTTAAACTTGCCCCAAACAGTGTTTGTTGGAGCAGAGAGG
Orangutan       ACAAGGTTGTATCTATTGAGGTTAAACTTGCCCCAAACAGTGTTTGTCGGAGCAGAGAGG
Gibbon          ACAAGGTTGTATCTATTGAGGTTAAACTTGCCCCAAACAGTGTTTGTTGGAGCACAGAGG
Baboon          ACAAAATTGTATCTATTGAGGTTAAACTTGCCCCAAACAGTGTTTGTTGGAGCAGAGAGG
Macaque         ACAAAATTGTATCTATTGAGGTTAAACTTGCCCCAAACAGTGTTTGTTGGAGCAGAGAGG
Rhesus          ACAAAATTGTATCTATTGAGGTTAAACTTGCCCCAAACAGTGTTTGTTGGAGCAGAGAGG
Marmoset        ACAAGGTTGTATCTATTGAGGTTAAACTTGCCCAAAACGGTGTTTGTTGGAGCAGAGAGG
Squirrel_monkey ------------------------------------------------------------
Tarsier         AACAAGGTTTAGCTATTGAGGTTGAACTCTCCCAAAAGGCTGTTTATTGGGGCAGAGGGG
Bushbaby        ACAAGGTTGTATCTATTGAGGTTAAACTTCCCCCAAAGGGGGTGTGTTGGAGCAGACAGC
Lemur           ACAAGGTTGTATCTATTGAGGTTAAACTTCCCCAAAAGGGTGTTTGTTGGAGCAGATGGG

Human           CAGAAAGCGTACA-------AAATAGGAAGGTGAGTTCTCGAAAGAGTTAAAATCCAGCC
Chimpanzee      CCGAAAGCGTACA-------AAATAGG-AGGTGAGTTGTCGAAAGAGTTAAAATCCAGCC
Gorilla         CAGAAAGCGTACA-------AAATAGGAAGGTGAGTTCTCGAAAGAGTTAAAATCCAGCC
Orangutan       CAGAAAGGGTCCA-------AAATAGGAAGGTGAGTTCTCAAAATAGTTAAAATCCAGCC
Gibbon          CAGAAAGCATACA-------AAATAAGAAGGTGAGTTCTCGAAAGAGTTAAAATCCAGCC
Baboon          AAGAAAGGGTACA-------AAATAGGAAGGTGAGTTCTCGGAAGAGTTAAAATCCAGCC
Macaque         AAGAAAGGGTACA-------AAATAGGAAGGTGAGTTCTCGAAAGAGTTAAAATCCAGCC
Rhesus          AAGAAAGGGTACA-------AAATAGGAAGGTGAGTTCTCGAAAGAGTTAAAATCCAGCC
Marmoset        CAGAAAGAGTGAA-------AAACAGGAAGGTGAGTTCTCAAAAGAGTTAAAATCCACCC
Squirrel_monkey ------------------------------------------------------------
Tarsier         CAGAATGGAGGCTATGTGTGAAACAGGAAAGTGGGTTCTCAAAAGAGTTAAAATACAGTC
Bushbaby        AGAAGTGTGTG---------AAATAGGAAGGTGGTGTCTCCGAGGAGCTGAAATCCAGCC
Lemur           CAGAAGGAAGGCTGCCTGTGAAATAGGAAGGTAGATTCTCAAAAGAGTTAAAATCCAGTC

Human           TTAAAAATTCC-ACATATGCTCAGTCAGGTAAATCATCATGAATTTTT------------
Chimpanzee      TTAAAAATTCC-ACATATGCTCAGTCAGGTAAATCATCATGAATTTTT------------
Gorilla         TTAAAAATTCC-ACATATGCTCAGTCAGGTAAATCATCATGAATTTTT------------
Orangutan       TTAAAAATTCC-ACATATGCTCAGTCAAGTAAATCCTCATGAATTTTT------------
Gibbon          TTAAAAATTCC-ACATATGCTCAGTCAAGTAAATCCTCATGAATTTTT------------
Baboon          TTAAAAATTCC-ACATATGCTCAGTCAAGTAAATCCTCATGAATTTTT------------
Macaque         TTAAAAATTCC-ACATATGCTCAGTCAAGTAAATCCTCATGAATTTTT------------
Rhesus          TTAAAAATTCC-ACATATGCTCAGTCAAGTAAATCCTCATGAATTTTT------------
Marmoset        TTAAAAATTCT-ACATATGCTCAGTCAAGTAAATCCTCATGATTTTTT------------
Squirrel_monkey ------------------------------------------------------------
Tarsier         TGTCAAAATTCTATATAATCTCAGTCAAGTAAATCCTCGTGGTTTTTTAAGCTGATATTT
Bushbaby        TGTCAAAATTC-TACCATCAGTC---AGGA-AGACCCTCGTGATATTT------------
Lemur           TGTCAGAATTCTATATATGCTCAGTCAAGTAAATCCTCATGATGTTTTATTTTAACCTAT

Human           -TACTGATATTTTGTCCTAGAAGATAGTGATATTAACATATTCTAGAAAAGAAAAACCTT
Chimpanzee      -TACTGATATTTTGTCCTAGAAGATAGTGATATTAACATATTCTAGAAAAGAAAAACCTT
Gorilla         -TACTGATATTTTGTCCTAGAAGATAGTGATATTAACATATTCTAGAAAAGAAAAACCTT
Orangutan       -TACTGATATTTTGTCCTAAAAGATAGTGATATTAACATATTCTAGAAAAGAAAAACCTT
Gibbon          -TACTGATATTTTGTCCTAGAAGATAGTGATATTAACATATTCTAGAAAAGAAAAACCTT
Baboon          TTACTGATACTTTGTCCTAGAAGATAGTGATATTAACATATTCTACAAAAGAAAAACCTT
Macaque         TTACTGATATTTTGTCCTAGAAGATAGTGATATTAACATATTCTAGAAAAGAAAAACCTT
Rhesus          TTACTGATATTTTGTCCTAGAAGATAGTGATATTAACATATTCTAGAAAAGAAAAACCTT
Marmoset        TAACTGATATTTTGTCCTAGAAGATAATGATATTAACGTATTCTAGAAAAGAAAAAGCTT
Squirrel_monkey ------------------------------------------------------------
```

```
Tarsier         TTACTGATATGTTGTTCTAGAAGATAGTAATCTTAGCATATTTTGGAAAAACTT------
Bushbaby        TAATCGATATTTTGTTCTGGGAGACAGTAATATTAGCGTATTCTAGAAAGGAAAATGATT
Lemur           ATTTTG---------TTTTGCAGATAGTAATGTTTGCATATTCTAGAAAGCAAA------

Human           GATTCAGTAAAAACCTCCTTAAGTGAGAGATACATGATTAAATGTTGATGACAAAATATA
Chimpanzee      GATTCAGTAAAAACCTCATTAAGTGAGAGATACATGATTAAATGTTGATGACAAAATATA
Gorilla         GATTCAGTAAAAACCTCATTAAGTGAGAGATACATGATTAAATGTTGATGACAAAATATA
Orangutan       GATTCAGTAAAAACCTCATTAAGTGAGAGATACATGATTAAATGTTGATGACAAAATATA
Gibbon          GATTCAGTAAAAACCTCATTAAGTGAGAGATACATGATTAAATGTTGATGACAAAATATA
Baboon          GATTCAGTAACAACCTCACTAAGTGAGAGATACGTGATTAAATGTTGATGACAAAATATA
Macaque         GATTCAGTAACAACCTCACTAAGTGAGAGATACGTGATTAAATGTTGATGACAAAATATA
Rhesus          GATTCAGTAACAACCTCACT----GAGAGATACGTGATTAAATGTTGATGACAAAATATA
Marmoset        GATTCAGCAA-AACCTCATTAAGTGGGAGATATATGACTAAATGTTGATAACAAAACATT
Squirrel_monkey ------------------------------------------------------------
Tarsier         TAATTCAGTAGAACATCACTAAGTAAGAGATATATGATTAAAAGATGTTGACAAAAATTG
Bushbaby        ACTTCAGTAAAACA-TTGTTGTGTGAGAAATGCATGATTAAATGTTGGTAGCAAAAAGTT
Lemur           AACTTTAGTAAAACATTACTGAGTGAGAGATACATGACTAAATGTTGT--CAAAAAATTA

Human           TTT-TGAGGTTATC-------TCAAAGTTTAATGAAGTTCTAAATATATAGAATGATTGA
Chimpanzee      TTT-TGAGGTTATC-------TCAAAGTTTAATGAAGTTCTAAATATATAGAATGATTGA
Gorilla         TTT-TGAGGTTATC-------TCAAAGTTTAATGAAGTTCTACATATATAGAATGATTGA
Orangutan       TTT-TGAGGTTATC-------TCAAAGTTTAATGAAGTTCTAAATATATAGAATGATTGA
Gibbon          TTT-TGAGGTTATC-------TCAAAGTTTAATGAAGTTCTAAATATACAGAATGATTGA
Baboon          TTT-TGAGGTTATCTCA-------AAGTTTAATGAAGTTCTAAATATATAGAATGATTGA
Macaque         TTT-TGAGGTTATCTCA-------AAGTTTAATGAAGTTCTAAATATATAGAATGATTGA
Rhesus          TTT-TGAGGTTATCTCA-------AAGTTTAATGAAGTTCTAAATATATAGAATGATTGA
Marmoset        TTT-TGAGGTTATTTCA--TATACAAGTTTAATGAAGTTCTAAATATATAGAATGACTAA
Squirrel_monkey ---------------------TTTTTTTTTAATGAATTTCTAAATATATAGAATGACTGA
Tarsier         -TTTCGAGGTTATCTCATATACTTAAGTTTAATGAAGTCCTAAATATCTAGAATTATTGC
Bushbaby        ATTTTGATGTTGTCTAATAGACTTAAATTGAATGAAGTGCTAAATATAGAAA-TGACCAC
Lemur           -TTTTGAGGTTATCTAATACACTTAAGTTTAATGAAGTTCTAAATATATAAAATGATTGC

Human           ATATACACT-TTAAGAAAACATGCAATAGGTGACTGGATAGAGCTGAAAAAGAGGCATCC
Chimpanzee      ATATACACT-TTAAGAAAACATGCAATAGGTGACTGGATAGAGCTGAAAAAGAGGCATCC
Gorilla         ATATACACT-TTAAGAAAACATGCAATAGGTGACTGGATAGAGCTGAAAAAGAGGCATCC
Orangutan       ATATACACT-TTAAGAAAACATGCAATAGGTGACTGGATAGAGCTGAAAAAGAGGCATCC
Gibbon          ATATACACT-TTAAGAAAACATGCAATAGGTGACTGGATAGTGCTGAAAAAGAGGCATCC
Baboon          ATATACACTTTTAAGAAAACATGCAATAGGAGACTAGATAGAGCTGAAAAAGAGGCATCC
Macaque         ATATACACTTTTAAGAAAACATGCAATAGGAGACTAGATAGAGCTGAAAAAGAGGCATCC
Rhesus          ATATACACTTTTAAGAAAACATGCAATAGGAGACTAGATAGAGCTGAAAAAGAGGCATCC
Marmoset        ATATACATT-TTAAGAAAACATGAAATAGGTGACTGGGTAGAGCTGAAAAAGAGGCATCC
Squirrel_monkey ATATACATA-TTAAGAAAACATGAAATAGGTGACTGGGTAGAGCTGAAAAAGAAGCATCC
Tarsier         AGACACACT-TTAATAAAACAT-----------------------AAAAAAAGGCATCC
Bushbaby        ATATACACT-TTGAGTAAACATGAAACAGA-TCATGAGTG---GAGTTGAAGAGGCAGCC
Lemur           CTAAGCACT-TTAAGTAAACATGAAATAGGTGACTGGGTGGAGCTGAAAAAGAAGCACCC

Human           ATATATTTTAGATT-----GTTC---ATTTCTTGTATATTTGATTTTATGTATGAGAGTG
Chimpanzee      ATATATTTTAGATT-----GTTC---ATTTCTTATATATTTGATTTTATGTATGAGAGTG
Gorilla         ATATATTTTAGATT-----GTTC---ATTTCTTATATATTTGATTTTATGTATGAGAGTG
Orangutan       ATATATTTTAGATT-----GCTC---ATTTCTTTTATATTTGATTTTATGTATGAGAGTG
Gibbon          ATATATTTTAGATT-----GTTC---ATTTCTTATATATTTGATTTTATGTATGAGAGTG
Baboon          ATAGATTTTAGATT-----GTTC---ATTTCTTATATATTTGATTTTATGTATGAGAGTG
Macaque         ATAGATTTTAGATT-----GTTC---ATTTCTTATATATTTGATTTTATGTATGAGAGTG
Rhesus          ATAGATTTTAGATT-----GTTC---ATTTCTTATATATTTGATTTTATGTATGAGAGTG
Marmoset        AAATATTTTAGATT-----GTTC---ATTTCTTATATACTTGATTTTATGCATGAGAGTT
Squirrel_monkey AAATATTTTAGATT-----GTTC---ATTTCTTATATACTTCATTTTATGTATGAAAGTG
Tarsier         AGAT------ATTTTGAACTATTCATTTCTT--ACATGTTTGCTTTTACATATGAGACTG
Bushbaby        AAGTGTTTTAGATT-----GTTCATTTTCATATATACATTTGATTTTATGTGTAACTGTG
Lemur           AAATATTAATATTTTAGATTGTTCATTTCTTATATATATTTGATTTTATGTGTGAGAGTG

Human           TTTGTTATCCTGATTTAGAATGAAATGACTTTGAGTGGTATTTGTGGATTCAATT-TTTT
Chimpanzee      TTTGTTATCCTGATTCAGAATGAAATGACTTTGAGTGGTATTTGTGGAATCAATTGTTTT
Gorilla         TTTGTTATCCTGATTTAGAATGAAATGGCTTTGAGTGGTATTTGTGGAATCAATT-TTTT
Orangutan       TTTGTTATCCTGATTCAGAATGAAATGACTTTGAGTGGTATTTGTGGAATAAATT-TTTT
Gibbon          TTTGTTATCCTGATTCAGAATGAAATGACTTTGAGTGGTATTTGTGGAATCAATT-TTTT
Baboon          TTTGTTATCCTGATTCAGACTGAAATGACTTTGGATGGTATTTGTGGAATCACTTTTTTT
Macaque         TTTGTTATCCTGATTCAGACTGAAACGACTTTGAATGGTATTTGTGGAATCACTTTTTTT
Rhesus          TTTGTTATCCTGATTCAGACTGAAACGACTTTGAATGGTATTTGTGGAATCACTTTTTTT
Marmoset        TTTGTCATCCTGATTCAGAATGAAATGACTTTGAATGGTATTTGTGGAATCACAT-TGTT
Squirrel_monkey TTTGTCATCCTGATTCAGAATGAAATGACTTTGCATGGTATTTGTGGAATCACAT-TGTT
Tarsier         TTTGTCACCCTGA---TTCAGAAAATGACTTTTAATGGTACTTGTAGCATCGTT--TTTT
Bushbaby        TTCATCATGCTTATTCTGAATAAAATGACCTTTAATGGCTCTTGTAGAATTACT--TTCT
Lemur           TTTGTCATGCTGATTCTGAATGAAATGACTTTTAATGGTACTTGTAGAATTACT--TTTT
```

```
Human          TCTAGCCTGAGGAATAATCTATTGTAGAAAAGTTGAAAAGTTGCTCTAATTCGCTAATAT
Chimpanzee     TCTAGCCTGAGGAATAATCTATTGTGGAAAAGTTGAAAAGTTGCTCTAATTCGCTAATAT
Gorilla        TCTAGCCTGAGGAATAATCTATTGTAGAAAAGTTGAAAAGTTGCTCTAATTCGCTAATAT
Orangutan      TCTAGCCTGAGGAATAATCTATTGTGGAAAAGTTGAAAAGTTGCTCTAATTCGCTAATAT
Gibbon         TCTAGCCTGAGGAATAATCTATTGTGGAAAAGTTGAAAAGTTGCTCTAATTCACTAATAT
Baboon         TCTAGCCTGAGGAATAATTTATTGTGGAAAAGTTGAAAAGTTGCTCTAATTCACTAATAT
Macaque        TCTAGCCTGAGGAATAATTTATTGTGGAAAAGTTGAAAAGTTGCTCTAATTCACTAATAT
Rhesus         CCTAGCCTGAGGAATAATTTATTGTGGAAAAGTTGAAAAGTTGCTCTCATTCACATATAT
Marmoset       TCTAGCCTGAGAAATAGTTCATTGTGAAAAAGTTGAAAAGTTGCTCTTATTTGCTAATAT
Squirrel_monkey TCTAGCCTGAGAAATAATTTATTATGGAAAAGTTGAAAAGTTGCTCTGATTTGCTAATAT
Tarsier        TCTCGCCCGAGAAATAATTTACTGTAGAAGAGTTGAAAAGTTGCTATAATTCACTAATAT
Bushbaby       TTTAGCCTGAGGAATAATTTATTGTGGAAGAGTTGAAAAGTTGCTCCAATTTGCTAACAT
Lemur          TCTAGCCCAAGGATTAATTTATTGTGGAAGAGTTAAAAAGGTACTTTACTTTGCTAATAT

Human          TTCAAAGCATCATTTATTTTCTCAGGGAAAGGA----------CCAGAAAACTCGGATCC
Chimpanzee     TTCAAAGCATCATTTATTTTCTCAGGGAAAGGA----------CCAGAAAAATCGGATCC
Gorilla        TTCAAAGCATCATTTATTTTCTCACGGAAAGGA----------CCAGAAAAATCGGATCC
Orangutan      TTCCAAGTATCATTTATTTTCTCAGGGAAAGGA----------CCAGAAAAATCAGATCC
Gibbon         TTCAAAGCATCATTTATTTTCTCAGGGAAAGGA----------CCAGAAAAATCAGATCC
Baboon         TTCAAAGCATCATTTATTTTCTCAGGGAAAGAA----------CCAGAAAAATTGGATCC
Macaque        TTCAAAGCATCATTTATTTTCTCAGGGAAAGAA----------CCAGAAAAATTGGATCC
Rhesus         TTNNNNNNNNNNNNNNNNNNNNNNNNNNN-----------------------NNNNNNNN
Marmoset       TTCAAAGCATCATTTATTTTTGCAGGGAAAGAA----------CTAGAAAAATCAATTCC
Squirrel_monkey TTCAAAGCATCATTAATTTTTGCAGGGAAAGAA----------CCAGAAAAATCAGTTCC
Tarsier        TTCAAAGCATCATTTATTTTGTTACGGAAATAACAAGAGAGAAGCAGAGAGATTAGTTCC
Bushbaby       TTTAAAGCATCATTTATTCTTACAGAGAAAGAA----------CCAGAAAGAATCAAATG
Lemur          TGTGAAGCATCATTTATTTTCTCAGAGA----------AAGAACCAGAAAGATTAGTTCC

Human          AAACTTAAAATGTCAATACTCATAGTTTAGCATTTC-AAAATGAATAACTACATGGAGTT
Chimpanzee     AAACTTAAAATGTCAATACTCATAGTTTAGCACTTA-AAAATGAATAACTACATGGAGAT
Gorilla        AAACTTAAAATGTCAATACTCATAGTTTAGCATTTC-AAAATGAATAAATACATGGAGTT
Orangutan      AAACTTAAAATAACAATACTCATAGTTTAGCATTTA-AAAATGAATAACTACATGGAGTT
Gibbon         AAACTTAAAATATCAATACTCATAGTTTAGCATTTA-AAAATGAATAACTACATGGAGTT
Baboon         AAACTTAAAATATCAATACTCATAGTTTAGCATTTA-AAAATGAATAACTATGTGGAGTT
Macaque        AAACTTAAAATATCAATATTCATAGTTTAGCATTTA-AAAATGAATAACTATGTGGAGTT
Rhesus         NNNNNNNNNNNNNNNNNNNNNNTAGTTTAGCATTTA-AAAATGAATAACTATGTGGAGTT
Marmoset       AAAATTAAAATATCAATACTCATAGTTTAGCATTTA-AAAATGAATAATTACATGGAGTT
Squirrel_monkey AAACTTAAAATATCAATACTCATAGTTTAGCATTTA-AAAATTAATAACTACATGGAGTT
Tarsier        AAATTTAAAATGTCAACACTGGTGGTTTGGCATTCA-AAAATGAGTAACTACATGGAGTT
Bushbaby       TA-----AAACATCAACACAGGTACTTTAGTGATCAAAAATGAGTAAGTTACGTGCAGTT
Lemur          AAATTTAAAATGTCAACACTAGTACTTTAGCATTCA-AAAATGAGTAACTACATGGAGTT

Human          ACCAGTGATACTTTTAAAATTTATATTTTTGCTATGTGTAC----TGTTTCAACAAAGTC
Chimpanzee     ACCAGTGATACTTTTAAAATTTATATT-TTTCTATGTGTAC----TGTTTCAACAAAGCC
Gorilla        ACCAGTGATACTTTTAAAATTTATATTTTTGCTATGTGTAC----TGTTTCAACAAAGTC
Orangutan      ACCAGTGATACTTTTAAAATTTATATTTTTGCTATGTGTAC----TGTTTCAACAAAGTC
Gibbon         ACCAGTGATACTTTTAAAATTTATATTTTTGCTATGTGTAC----TGTTTCAACAAAGTC
Baboon         ACCAGTGATACTTTTAAAATTTATATTTTTGCTATGTGTAC----TGTTTCAACAAAGTC
Macaque        ACCAGTGATACTTTTAAAATTTATATTTTTGCTATGTGTAC----TGTTTCAACAAAGTC
Rhesus         ACCAGTGATACTTTTAAAATTTATATTTTTGCTATGTGTAC----TGTTTCAACAAAGTC
Marmoset       ACCAGTGATATTTTTAAAATTTATATTTTTGCTATGTGTAC----TGTTTAAACAAAGTC
Squirrel_monkey ACCAGTGATATTTTAAAAATTTACATTTTTGCTATATGCAC----TGTTTAAACAAAGTC
Tarsier        ACCAGTGATATTTTTAAAATTTATATTCTTGTTATGTGTACTGTTTGTT-TTGCAAAATC
Bushbaby       ACCAGTGATACTTTAAGAATTTATCTTCTTGCCATGTGTACAGTTTGTTTTGGCAAAGTC
Lemur          ACCAGTTATACTTTTAAAATTTATATTCTTGCTATATGTATAGTTTTTT-TGGCCAAATC

Human          ATGAGTATACCAAAGCGAGCTAACAGTCTTCATTCCAAAATGTTGCCCCAAAATAATTTC
Chimpanzee     ATGAGTATACCAAAGCGAGCTAACAGTCTTCATTCCAAAATGTTGCCCCAAAATAATTTC
Gorilla        ATGAGTATACCAAAGCGAGCTAACAGTCTTTATTCCAAAATGTTGCCCCAAAATAATTTC
Orangutan      ATGAGTATACCAAAGCGAGCTAACAGTCTTCATTCCAAAATGTTGCCCCAAAATAATTTC
Gibbon         ATGAGTATACCAAAGCGAGCTAAGTCT--TCATTCCAAAATGTTGCCCCAAAATAATTTC
Baboon         ATGAGTATACCAAAGTGAGCTAACAGTCCTCATTCCAAAATGTTGCCCCAAAATAATTTC
Macaque        ATGAGTATACCAAAGTGGGCTAACAGTCCTCATTTCAAAATGTTGCCCCAAAATAATTTC
Rhesus         ATGAGTATACCAAAGTGGGCTAACAGTCCTCATTTCAAAATGTTGCCCCAAAATAATTTC
Marmoset       GTAAGTATACCAAAGTGGGCTAACAGT--CTTC-ATTCCAAAATGTCCCAAAATAATTTC
Squirrel_monkey ATAAGTATACCAAAGTGGGCTAACAGC--CTTC-ATTCCAAAATGTCCCAAAATAATTTC
Tarsier        ATAAGTATTCCAAAGTGAACGAACAGTGTTCATTCCAAAACATTCCCCCAAAATAATTTC
Bushbaby       ATAAGTATACCAAGGTAAGCTAACAGCCTTCATTTCGAAATGTTCCCCTCAAATAATTTC
Lemur          TTAAGTATACCAAGGTGAGCTAACAGTCTTCATTTCAAAATGTTCCCCTCAAATAATTTC

Human          TGAACCACATGACAGATGTTTGATACAGATGACTAAATTTCCTGTCATGTTTGTTTATTG
Chimpanzee     TGAACCACATGACAGATGTTTGATACAGATGACTAAATTTCCTGTCATGTTTGTTTATTG
```

```
Gorilla         TGAACCACATGACAGATGTTTGATACAGATGACTAAATTTCCTGTCATGTTTGTTTATTG
Orangutan       TGAACCATATGACAGATGTTTGATACAGATGACTAAATTTCCTGTCATGTTTGTTTATTG
Gibbon          TGAACCACATGACAGATGTTTGATACAGATGACTAAATTTCCTGTCATGTTTGTTTATTG
Baboon          TGAACCACATGACAGATGTTTGATACAGATGACTAAATTTCTTGTCATGTTTATTGATAC
Macaque         TGAACCACATGACAGATGTTTGATACAGATGACTAAATTT--------------------
Rhesus          TGAACCACATGACAGATGTTTGATACAGATGACTAAATTT--------------------
Marmoset        TGAACCACATGACAGATGTTTGATACAGATAACTAAATTTTCTGTTATGTTTGTTTATAG
Squirrel_monkey TGAACCACATGACAGATGTTTGATACAGATAACTAAATTTT--GTTATGTTTGTTTATAG
Tarsier         TGAACCACATGGCAGAAATTCGATACAGGTGACTAATTTTTTTATTATTATTTATTGTTA
Bushbaby        TGAACCATATGGCATAAATTTGATACAGATGACTAAATTTGCTGTCAGATTTGGTTATGA
Lemur           TGAACTAAATGGCAGAAATCTGATACAGATGACTAAATTTGCTGTCATATTCGGGTATTA

Human           TTAAT----------------------------AGTAAAACCAACAGAGAGTGCTAGACT
Chimpanzee      TTAAT----------------------------AGTAAAACCAACAGAGAGTGCTAGACT
Gorilla         TTAAT----------------------------AGTAAAACCAACAGAGAGTGCTAGACT
Orangutan       TTAAT----------------------------AGTAAAACCAACAGAGAGTGCTAGACT
Gibbon          TTAAT----------------------------AGTAAAACCAAC----AGTGCTAGACT
Baboon          AGATGACTAAATTTCTTGTCATGTTTATTGTTAATAGTAAAACCAACAGAGTGCTAGACT
Macaque         --------------CTTGTCATGTTTATTGTTAATAGTAAAGCCAACAGAGTGCTAGACT
Rhesus          --------------CTTGTCATGTTTATTGTTAATAGTAAAGCCAACAGAGTGCTAGACT
Marmoset        TTAAT----------------------------AGTAAAACCAACAGAGAGTGCTAGGCT
Squirrel_monkey TTAAT----------------------------AGTAAAACCAACAGAGAGTGCTAGACG
Tarsier         ATTGTAA-------------------------------AACCA--ACAAAGTGCTAGAC-
Bushbaby        TTAATAGC----------------------------AAAACCAACAGGGAATGCTAGACT
Lemur           TTAATAGT----------------------------AAAACCAACAGAGAATGCTAGAC-

Human           TTAGACACTAATATCTTCAAAATATGGACTTACATGAAACTAGACAGCAATATCATCTTA
Chimpanzee      TTAGACACTAATATCTTCAAAATATGGACTTACATGAAACTAGACAGTGATATCATCTTA
Gorilla         TTAGACACTAATATCTTCAAAATATGGACTTACATGAAACTAGACAGCGATATCATCTTA
Orangutan       TTAGACACTAATGTCTTCAAAATATGGACTTACATGAAACTAGACAGCAATATCATCTTA
Gibbon          TTAGACACTAATATCTTCAAAATATGGACTTACATGAAACTAGACAGCAATATCATCTTA
Baboon          TCAGACACTAATATCTTCAAAATATGGACTTACATGAAACTAGACAGCGACATCATCTTA
Macaque         TCAGACACTAATATCTTCAAAATATGGACTTACATGAAACTAGACAGCGACATCATCTTA
Rhesus          TCAGACACTAATATCTTCAAAATATGGACTTACATGAAACTAGACAGCAACATCATCTTA
Marmoset        CTAGACAAGCATATCTTAGAAATATAAACTTACGTGAAACTAGACAGAAATATCATCTCA
Squirrel_monkey TTAGACAAACATATCTTAGAAATATAAACTTACATGAAACTAGACAGAAATATCATCTCA
Tarsier         TTAGGAACTAATACATTAGAAATATGGGTTTACATGAAACCAGACAGAGATATCAATTTA
Bushbaby        TTAGATGCTAA-----TACTATTATGAACTTGCACGAAACCATACAGATGTATCTTCTTA
Lemur           TTTAGAACTAATACCT-TAAAATATGAACTTATGTGAAACCATAAGGAGATATCATCTTA

Human           AAGTACTTTTTTCTCTTAAACAAACAAATCGAAAAAAGCAAAGATCAGAGATGAAAGGAA
Chimpanzee      AAGTACTTTTTTCTCTTAAACAAACAAATCGAAAAAAACAAAGATCAGAGATGAAAGGAA
Gorilla         AAGTACTTTTTTCTCTTAAACAAACAAATCGAAAAAAGCAAAGATCAGAGATGAAAGGAA
Orangutan       AAGTACTTTTTTCTCTTAAACAAACAAATCGAAAAAAGCAAAGATCAGAGATGAAAGGAA
Gibbon          AAGTACTTTTTTCTCTTAAACAAACAAATCAAAAAAAGCAAAGATCAGAGATGAAAGGAA
Baboon          AAGTACTTTTTTCTCTTAAACAAACAAATCAAAAAAAGCCAAGATCAGAGTTGAAAGGAA
Macaque         AAGTACTTTTTTCTCTTAAACAAACAAATCAAAAAAAGCCAAGATCAGAGTTGAAAGGAA
Rhesus          AAGTACTTTTTTCTCTTAAACAAACAAATCAAAAAAAGCCAAGATCAGAGTTGAAAGGAA
Marmoset        AAGTACTTTTTTCTCTTAAACGAA----CCAAAAAAAGCAAAGATCAGAGATGAAAGGAA
Squirrel_monkey AAGTACTTTTTTCTCTTAAACAAACAAACCGAAAAAAGCAAAGATCAGAGATGAAAGGAA
Tarsier         AAGTACTTTATCGTTT-AAACAAACAATACAAAACAAGCAAAGTCCAGAAAGGAAGGGCA
Bushbaby        AAGAACTTTTTCTCTTGAGCAAACAAACCAGGGAAAAGCAAAGATCAGTGTTAAAGGGAA
Lemur           AAGTACTTTTTCACTT-GAACAAACAAACCAAAAAAATCAAAGATCAGAGATGGTGGGAA

Human           GAAAAGTATATCGAGTGACACCATGTGTCAGCCAGACACTGGGATAGATGCTTCATATAC
Chimpanzee      GAAAAGTATATCGAGTGACACCATGTGTCAGCCAGACGCTGGGATAGATGCTTCATATAC
Gorilla         GAAAAGTATATCGAGTAACACCATGTGTCAGCCAGACACTGGGTTAGATGCTTCATATAC
Orangutan       GAAAAGTATATCGAGTGACACCATATGTCAGCCAGACACTGGGATAGGTGCTTCATATAC
Gibbon          GAAAAGTATATCGAGTGACACCATATGTCAGCCAGACACTGGGATAGATGCTTCATATAC
Baboon          GAAAAATACATTGAGTGACACCATATGGCAGCCAGACACTGGGATAGATGCTTCATATAC
Macaque         GAAAAATACATTGAGTGACACCATATGGCAGCCAGACACTGAGGTAGATGCTTCATATAC
Rhesus          GAAAAATACATTGAGTGACACCATATGGCAGACAGACACTGAGGTAGATGCTTCATATAC
Marmoset        GAAAAATATATTGAGCGACACTACATGTCAGCTAGACCCTGGGATAGATGCTTTATATAC
Squirrel_monkey GAAAAATACATTGAGCGACACTACATGTCAGCCAGACACTGGGATAGATGCTTTATATAC
Tarsier         GAAAACTATGCTGAGT--GCACTATAGCCAGCCAGACACTGGGATAGACGCTTCATATAA
Bushbaby        TTCACATACGGTTAGTGACGCTCTATGCCAGCCAGGTACTGGGATAGACGCTTTACATAC
Lemur           GAAACATACATTTAGTGATCCTATATGCTATCCAGACACTGGGATAGATACTTTACATAC

Human           CTTAGGTCATTTAACGTTATAGTTAAGTAGAAAATG------------TTTTAAAATTGT
Chimpanzee      CTTAGGTCATTTAACGTTATAGTTAAGTAGAAAATG------------TTTTAAAATTGT
Gorilla         CTTAGGTCATTTAACGTTATAGTTAAGTAGAAAATG------------TTTTAAAATTGT
Orangutan       CTTAGGTCATTTAACATTATAGTTAAGTAGAAAATG------------TTTTAAAGTTGT
Gibbon          CTTAGGTCATTTAACGTTATAGTTAAGTAGAAAATG------------TTTTTAAGTTGT
```

```
Baboon          CTTAGGTCATTTAACGTTATAGTTAAGTAGAAAATG------------TTTTAAAGTTGT
Macaque         CTTAGGTCATTTAACGTTATAGTTAAGTAGAAAATG------------TTTTAAAGTTGT
Rhesus          CTTAGGTCATTAAACGTTATAGTTAAGTAGAAAATG------------TTTTAAAGTTGT
Marmoset        CTTAGGTCATTTAATGTTTTAGTTAAGTAGAAGATG------------TTTTAAAGTTGT
Squirrel_monkey CTTAGGTCATTTAATGTTTTAGTTAAGTAGAAGATG------------TTTTAAAGTTGT
Tarsier         CTGAGGTCACTTAACATTA---------AGTGAAA----CAGGAAATGTTTCAAAGTTGT
Bushbaby        CTTACATCATTGAGAACTGTGGTTAAATAAAGAGCTAAACAGAAAATGTGTCAAGGTTGT
Lemur           CTTAGGTCATTTAACATTATGGTTAAATAGCTAAA----CAGAAAATGTTTTCAACTTGT

Human           GGAGGTGTT-TAACTAGTTTTTCATTTTCTAAGCCAGCACAGCTGTTGACTCACCTGTTC
Chimpanzee      GGAGGTGTT-TAACTAGTTTTTCATTTTCTAAGCCAGCACAGCTTTTGACTCACCTGTTC
Gorilla         GGAGGTGTT-TAACTAGTTTTTCATTTTCTAAGCCAGCACAGCTTTTGACTCACCTGTTC
Orangutan       GGAGGTGTTTTAACTAGTTTTTCATTTTCTAAGCCAGCACAGCTTTTGATTCACCTGTTT
Gibbon          GGAGGTGTT-TAACTAGTTTTTCATTTTCTAAGCCAGCACAGCTTTTGACTCACCTGTTC
Baboon          GGAGGTATT-TAACTAGTTTTTCATTTTCTAAGCCAGCACAGCTTTTGACTCACCTGTTC
Macaque         GGAGGTATT-TAACTAGTTTTTCATTTTCTAAGCCAGCACAGCTTTTGACTCACCTGTTC
Rhesus          GGAGGTATT-TAACTAGTTTTTCATTTTCTAAGCCAGCACAGCTTTTGACTCACCTGTTC
Marmoset        GAAGGTGTT-TAACTAATTTTTCATTTTCTAAGTCAGTACAGCTTTTGACTCACCTGTTC
Squirrel_monkey GAAGGTGTT-TAACTAGTTTTTCATTTTCTAAGTCAGCACAGCTTTTGACTCACCTGTTC
Tarsier         GGAGATGTC-CCACTAGTTTTTCATTTTCTAAGCCAGCACAACCACTGACTCACAGGTTC
Bushbaby        GGAGG-----TGTTTAATTTTTCATCTTCTAAGTGAGCACAATCCTTGACTCACAGGTTC
Lemur           GGAGGTGTT-TAACTAGTTTCTCATTTTCTATGGGAGCATAACCCTTGATTCACATGTTC

Human           ATAATGCAGAGATTATTTGTTCTGCCCTACGTTTCTGTTGCACAACATTTTCCCTCTCTT
Chimpanzee      ATAATGCAGAGATTATTTGTTCTGCCCTACGTTTCTATTGTACAACATTTTCCCTCTTTT
Gorilla         ATAATGCAGAGATTATTTGTTCTGCCCTACGTTTCTGTTGCACAACATTTTCCCTCTCTT
Orangutan       ATAATGCAGAGATTATTTGTTCTGCCCTACGTTTCTGTTGCAC---ATTTTCTCTCTTTT
Gibbon          ATAACGCAGAGATTACTTGTTCTGCCCTACGTTTCTGTTGCAC---ATTTTCTCTCTTTT
Baboon          ATAATGCAGAGATTATTTGTTCTGCCCTGCATTTCTGTTGCACAACATCTTCTCTCTTTT
Macaque         ATAATGCAGAGATTATTTGTTCTGCCCTGCGTTTCTGTTGCACAACATTTTCTCTCTTTT
Rhesus          ATAATGCAGAGATTATTTGTTCTGCCCTGCGTTTCTGTTGCACAACATTTTCTCTCTTTT
Marmoset        C-AATGCAAAGATTATTTGTTCTACCCTATGTTTCTGTTGCACAACATTTTCTCGCTTTT
Squirrel_monkey ACAATGCAAAGATTATTTGTTCTACCCTATGTTTCTGTTACACAACCTTTTCTCGCTTTT
Tarsier         ATACTGCAGAGATCATTTGCACTACCCTTTGTTTCTCTGGCACAAGATCTTCTCTCTTTT
Bushbaby        ATAATGTGAAGATTATTTGCTCTGTCCTG---------------TGCTTTTCTCTTTTAT
Lemur           ATCATGCAAAGATTATTTGCTCTGCCCTATGTTTCTCTTGCACAAGATTTTCTGTCTTTT

Human           AAAAGTCATTTA-----TTGAATCATCATCTGTCTCAGGCTTGATACGGTATAGCCTCTG
Chimpanzee      AAAAGTCATTTA-----TTGAATCATCACCTGTCTCAGGCTTGATACGGTATAGCCTCTG
Gorilla         AAAAGTCATTTA-----TTGAATCATCATCTGTCTCAGGCTTGATACTGTATAGCCTCTG
Orangutan       AAAAGTCATTTA-----TTGAATCATCATCTGTCTCAGGCTTGATACGGTATAGCCTCTG
Gibbon          AAATGTCATTTA-----TTGAATCATCATCTGTCTCAGGCTTGATATGGTATAGCCTCTG
Baboon          AAAAGACATTTA-----TTGAATCATCATCTGCCTCAGGCTTGATATGGCATAGCCTCTG
Macaque         AAAAGACATTTA-----TTGAATCATCATCTGCCTCAGGCTTGATATGGCATAGCCTCTG
Rhesus          AAAAGACATTTA-----TTGAATCATCATCTGCCTCAGGCTTGATATGGCATAGCCTCTG
Marmoset        AAAAGTCATCTA-----TTGAATCATCATCTGCCTCAGGCTTGATATGGTATAGCCTCTA
Squirrel_monkey AAAAGTCATTTA-----TTGAATCATCATCTGCCTCAGGCTTGATATGGTATAGCCTCTA
Tarsier         ATTCAAGAAATCTTATTTTTGAATCATCATCTGTCAGGCTTGAGATGGTGTAGTCTCTG
Bushbaby        AAATCTTGTTTC-----TTGAATCATCGTCTGCCTCAGGCTTGATGTGATGTACTCTCCA
Lemur           ATAAGCCTTCCTTCTTGA---TTCATCATCTGCCTCAGGCTGAATGTGGTGTAGTCTCTG

Human           AAAGACTGGTTTAG-TACTTAAACCCACCTTTCAAAATATTTCC-AGTGCAGGC---CAT
Chimpanzee      AAAGACTGGTTTAG-TACTTCAACCAGCCTTTCAAAATATTTCC-AGGGCAGGC---CAT
Gorilla         AAAGACTGGTTTAG-TACTTCAACCCGCCTTTCAAAATATTTTC-AGTGCAGGC---CAT
Orangutan       AAAGACTGGGTTTAGTACTTCAACTCGCCTTTCAAAATATTTCCAGTTGCAGGC---CAT
Gibbon          AAAGACTGGTT-TAGTACTTCAACTCGCCTTTCAAAATATTTCCAG-TGCAGGC---CAT
Baboon          AAAGACTGGTT-TAGTACTTCAACTCACCTTTCAAAATATTTCCAG-TGCAGGC---CAT
Macaque         AAAGACTGGTT-TAGTACTTCAACTCACCTTTCAAAATATTTCCAG-TGCAGGC---CAT
Rhesus          AAAGACTGGTT-TAGTACTTCAACTCACCTTTCAAAATATTTCCAG-TGCAGGC---CAT
Marmoset        AAAGACTGGTT-TCGTACTTCAGCTAGCCTTTCAAAATATTTCCAG-TGCAGGC----CT
Squirrel_monkey A-AGACTGGTT-TAGTACTTCAGCTCGCCTTTCAAAATATTTCCAG-TGCAGGC----CT
Tarsier         CCAGACTGGT-TTAGTGCATCAACTCACCTTTC-AAATATTTCCAGTGCAGGCCACTCAT
Bushbaby        AGAGACTGCT-TTAGTACTTCAACTCACCTTTCAAAATATTTCCAGTGAGGGCCACTTGT
Lemur           AAAGACTGGT-TTAGAACTTCAACTCACCTTTCAAAATATTTCCAGTGCAGGCCACTCAC

Human           TCAGTGTTCCAGGTACCTAATGAGTCATTTTCTTCTTCC-CAGTCTACCAAGCAGGATCT
Chimpanzee      TCAGTGTTCCAGGTACCTAATGAGTCATTTTCCTCTTCC-CAGTCTACCAAGCAGGATCT
Gorilla         TCAGTGTTCCAGGTACCTAATGAGTCATTTTCTTCTTCC-CAGTCTACCAAGCAGGATCT
Orangutan       TCAATGTTCCAGGTACCTAATGAGTCATTTCCTCTTCCC-AGTCTANNNNNNNNNNNNNN
Gibbon          TCAGTGTTCCAGGTACCTAATGAGTCATTTTCCTCTTCCCAGTCTACCAAGCAGGATCTC
Baboon          TCAGTGTTCCAGGTACCTAATGAGTCATTTTCATCTTCCCAGTCTACCAAGCAGGATCTC
Macaque         TCAGTGTTCCAGGTACCTAATGAGTCATTTTCATCTTCCCAGTCTACCAAGCAGGATCTC
Rhesus          TCAGTGTTCCAGGTACCTAATGAGTCATTTTCATCTTCCCAGTCTACCAAGCAGGATCTC
```

```
Marmoset        TCACTGTTCCAGGTACCTAATGAGTCATTTTCCTCTTCCCAGTCTACCAAGCAGGATCTC
Squirrel_monkey TCAGTGTTCCAGGTACCTAATGAGTCATTTTCCTCTTCCCAGTCTACCAAGCAGGATCTC
Tarsier         TCAGTGTCCCAGGTGCCCACTGAGTCATTTTCCTCTTTCCAGTAAACTGAGCAGGATATC
Bushbaby        TCAGTGTCCCGAGGACCTAATGAGTCATTTTCCTCTTCCCAATTAAATGAACAGGATATC
Lemur           TCAGTATCCCAGGTACCTAATGAGTCATTTTCCTCTTCCCAATCTAGTGAACAGGGTATC

Human           CAAAGCCTCCTTTATCTTTCTGGGTGTATACAGAG-------------------------
Chimpanzee      CAAAGCCTCCTTTATCTTTCTGGGTGTATACAGAG-------------------------
Gorilla         CAAAGCCTCCTTTATCTTTCTGGGTGTATACAGAG-------------------------
Orangutan       NNNNNNNNNNNNNNNNNNNNNNNNNNNNNNNNNNNNNNNNNNNNNNNNNNNNNNNNNNNN
Gibbon          AAAGCCTCCTTTATCTTTCTGGGTGTATA--CAGTCATTAAGCTCAAATATGTCCTTTGC
Baboon          AAAGCCTCCTTTATCTTTCTGGGTGTACACAGAGTCATTAAGTTCAAATATGTCCTTTGC
Macaque         AAAGCCTCCTTTATCTTTCTGGGTGTATACAGAGTCATTAAGCTCAAATATGTCCTTTGC
Rhesus          AAAGCCTCCTTTATCTTTCTGGGTGTACACAGAGTCATTAAGCTCAAATATGTCCTTTGC
Marmoset        AAAGCCTCCTTTATCTTTCTGGGTGGATACAGAGTCATTAAACTTAAATATGTCCTTTGC
Squirrel_monkey AAAGCCTCCTTTATCTTTCTGGGTGGATACAGAGTCATTAAACTTAAATATGTGCTTTGC
Tarsier         AAAGCCTCCTTTATCTTTCTGGGTGGATACAGAGTCATTAAGCTTAAACATGCCCTTTGC
Bushbaby        AAAGCCTCCTTTATCTTTCTGGGTGGGTACCGAGTCATTAAGCTTTGCCAATAGACTTGA
Lemur           AAACCCTCCTTTATCTTTCTGGGTGGATACAGAGTCATTAAGCTTAAATATGCCCTTTGC

Human           ------------------------------------------------------------
Chimpanzee      ------------------------------------------------------------
Gorilla         ------------------------------------------------------------
Orangutan       NNNNNNNNNNNNNNNNNNNNNNNNNNNNNNNNNNNNNNNNNNNNNNNNNNNNNNNNNNNN
Gibbon          CAATAGACTT-GGGGGTTGGGT---TTAGT-TATTAACTTGGTGA-GTCTCCACCCAG-C
Baboon          CAACAGACTTGAGGGTTGGGTTTAGTTATTAACTT-----GGTTGAGTCTCCACCCAGCT
Macaque         CAACAGACTTGAGGGTTGGGTTTAGTTATTAACTT-----G-GTGAGTCTCCACCCAGCT
Rhesus          CAACAGACTTGAGGGTTGGGTTTAGTTATTAACTT-----G-GTGAGTCTCCACCCAGCT
Marmoset        CAATAGACTT-GAGGATTGGGC---TTAGT-TATTAACTTGGTGA-GTCTCCACCCAG-C
Squirrel_monkey CAATAGACTT-GAGGGTTGGGT---TTAGT-TATTAACTTGGTGA-GTCTCCACCCAG-C
Tarsier         CAATAGACTTGAGAGCTGGGTTTAGTTATTAACTTGCTGAGCCT------CCA-CCCAGC
Bushbaby        GGGTTGGGTTTACTTAT----------------TAACTTGGTGAGCCTCC-G-CCCAGC
Lemur           CAATAGACTTGAGGGTTGGGTTTAGTTATTAACTTGGTGAGCCT------CCG-CCCAGC

Human           ------------------------------------------------------------
Chimpanzee      ------------------------------------------------------------
Gorilla         ------------------------------------------------------------
Orangutan       NNNNNNNNNNNNNNNNNNNNNNNNNNNNNNNNNNNNNNNNNNNNNNNNNNNNNNNNNNNN
Gibbon          TAACTATTCT-AGGAAATTAT--------------TTGGTGAGTCAGGCATTCTTGTATT
Baboon          -AACTATTCTAGGAAA---------------TCATTTGGTGAGTCAGGCATTCTTGTATT
Macaque         -AACTATTCTAGGAAA---------------TCATTTGGTGAGTCAGGCATTCTTGTATT
Rhesus          -AACTATTCTAGGAAA---------------TCATTTGGTGAGTCAGGCATTCTTGTATT
Marmoset        TAACTATTCT-AGGAAATCAT--------------CTGGTGAGTCAGGCATTTTTGTATT
Squirrel_monkey TAACTATTCT-AGGAAATCAT--------------TTGGTGAGTCAGGCATTTTTGTATT
Tarsier         TAACTATTCTAGGAAATA---------------ATCTGGTGAGTCAGGTGTTTTCATATT
Bushbaby        TAACTATTCTAGGAAATA---------------ATTTGGTGAGTCAGGTGTTTTCATATT
Lemur           TAACTATTCTAGGAAATA---------------ATTTGGTGAGTCAGGTGTTTTCATATT

Human           ------------------------------------------------------------
Chimpanzee      ------------------------------------------------------------
Gorilla         ------------------------------------------------------------
Orangutan       NNCCTTTCAAAAATATTTGCAGTGCAGGCCATTCAATGTTCCAGGTACTAATGAGTCATT
Gibbon          CACATCGCA---------------------------------------------------
Baboon          CACATTGCA---------------------------------------------------
Macaque         CACATTGCA---------------------------------------------------
Rhesus          CACATTGCA---------------------------------------------------
Marmoset        CACATTGCA---------------------------------------------------
Squirrel_monkey CACATTGCA---------------------------------------------------
Tarsier         CATATTTCAT--------------------------------------------------
Bushbaby        CATATTTCAT--------------------------------------------------
Lemur           CAAGTTTCAT--------------------------------------------------

Human           -----------------------------------------------------------T
Chimpanzee      -----------------------------------------------------------T
Gorilla         -----------------------------------------------------------T
Orangutan       TCCTCTCCAGTCTACAAGCGATCTCAAAGCCTCCTTTATCTTTCTGGGTGTATACAGAGT
Gibbon          ------------------------------------------------------------
Baboon          ------------------------------------------------------------
Macaque         ------------------------------------------------------------
Rhesus          ------------------------------------------------------------
Marmoset        ------------------------------------------------------------
Squirrel_monkey ------------------------------------------------------------
Tarsier         ------------------------------------------------------------
```

```
Bushbaby        ------------------------------------------------------------
Lemur           ------------------------------------------------------------

Human           CATTAAGCTCAAATATGTCCTTTGCCAATGGACTTGAGGGTTGGGTTTAGTTATTAACTT
Chimpanzee      AATTAAGCTCAAATATGA------------GACTTGAGGGTTGGGTTTAGTTATTAACTT
Gorilla         CATTAAGCTCAAATATGTCCTTTGCCAATGGACTTGAGGGTTGGGTTTAGTTATTAACTT
Orangutan       CATTAAGCTCAAATATGTCCTTTGCCAATAGACTTCAGGGTTGGGTTTAGTTATTAACTT
Gibbon          ------------------------------------------------------------
Baboon          ------------------------------------------------------------
Macaque         ------------------------------------------------------------
Rhesus          ------------------------------------------------------------
Marmoset        ------------------------------------------------------------
Squirrel_monkey ------------------------------------------------------------
Tarsier         ------------------------------------------------------------
Bushbaby        ------------------------------------------------------------
Lemur           ------------------------------------------------------------

Human           GGTGAGTCTCCACCCAGCTAACTATTCTAGGAAATCATTTGGTGAGTCAGGCATTCTTGT
Chimpanzee      GCTGAGTCTCCACCCAGCTAACTATTCTAGGAAATCATTTGGTGAGTCAGGCATTCTTGT
Gorilla         GGTGAGTCTCCACCCAGCTAACTATTCTAGGAAATCATTTGGTGAGTCAGGCATTCTTGT
Orangutan       GGTGAGTCTCCACCCAGCTAACTATTCTAGGAAATCATTTGGTGAGTCAGGCATTCTTGT
Gibbon          ------------------------------------------------------------
Baboon          ------------------------------------------------------------
Macaque         ------------------------------------------------------------
Rhesus          ------------------------------------------------------------
Marmoset        ------------------------------------------------------------
Squirrel_monkey ------------------------------------------------------------
Tarsier         ------------------------------------------------------------
Bushbaby        ------------------------------------------------------------
Lemur           ------------------------------------------------------------

Human           ATTCACATTGCA
Chimpanzee      ATTCACATTGCA
Gorilla         ATTCACATTGCA
Orangutan       ATTCACATTGCA
Gibbon          ------------
Baboon          ------------
Macaque         ------------
Rhesus          ------------
Marmoset        ------------
Squirrel_monkey ------------
Tarsier         ------------
Bushbaby        ------------
Lemur           ------------
```
